# A systematic analysis of the global, regional, and national burden of fungal skin diseases from 1990 to 2021

**DOI:** 10.3389/fepid.2024.1489148

**Published:** 2024-12-16

**Authors:** Hongping Wang, Fengjun Sun, Changquan Wang, Jin Ye, Peiyuan Xia, Wanneng Wang, Yaguang Wu

**Affiliations:** ^1^ Department of Pharmacy, School of Pharmacy and Bioengineering, Chongqing University of Technology, Chongqing, China; ^2^ Department of Pharmacy, The First Affiliated Hospital of Army Medical University (Third Military Medical University), Chongqing, China; ^3^ Department of Refractive Surgery, Chongqing Bright Eye Hospital, Chongqing, China; ^4^ Department of Dermatology, The First Affiliated Hospital of Army Medical University (Third Military Medical University), Chongqing, China

**Keywords:** fungal skin diseases, GBD 2021, prevalence, incidence, disability-adjusted life years, percentage change

## Abstract

**Purpose:**

We aimed to assess the burden of Fungal Skin Diseases (FSD) in 2021 and explore the changing trends from 1990 to 2021 across different age groups and time periods.

**Methods:**

This study extracted three key indicators of the burden of FSD from the Global Burden of Disease (GBD) 2021 study: prevalence, incidence, and disability-adjusted life years (DALYs). The results were presented using point estimates and Uncertainty Intervals (UIs), and secondary analysis was conducted on these data to assess the changing trends in the burden of FSD using percentage change.

**Results:**

In 2021, the global cases of prevalence, incidence, and DALYs of FSD were reported at 616.5 million, 1,729.2 million, and 3,429.5 thousand, respectively, an increase of approximately 68% since 1990. The age-standardized rates per 100,000 population for prevalence, incidence, and DALYs were 7,789.6, 21,668.4, and 43.4, respectively. These rates represent percentage increases of 6.21%, 3.74%, and 6.56% since 1990. In terms of age distribution, the ages for FSD-related prevalence, incidence, and DALYs peak cases globally were in the 5–9 age group, with distinct age groups observed in low and low-middle, middle, high-middle and high SDI regions at 5–9 years, 45–49 years, and 70–74 years, respectively.

**Conclusion:**

Over the past 32 years, there has been a significant increase in the global burden of FSD. With improvements in the Socio-Demographic Index (SDI), the age groups for FSD-related peak cases are gradually shifting towards older age groups. This indicates the need to allocate healthcare resources rationally to address the challenges arising from the significant differences in geographic distribution, gender, and among different populations.

## 1 Introduction

Fungal skin diseases (FSD) caused by dermatophytes and non-dermatophytes are among the most prevalent dermatological conditions, primarily affecting the skin, hair, and nails (1). According to the Global Burden of Disease (GBD) 2019, the global prevalence of FSD reached 1.65 billion, with an age-standardized prevalence rate (ASPR) of 21.4% and an age-standardized incidence rate (ASIR) of 21.2%, posing a significant challenge to global public health (2). Dermatophyte infections, predominantly caused by species of Trichophyton, Epidermophyton, Microsporum, and Nannizzia, are the primary pathogens responsible for skin, hair, and nail infections globally, accounting for 60%–70% of all cases (3, 4). The epidemiology of these infections is dynamic, varying by geographic location and specific populations, with incidence rates as high as 36.6%–78.4% in certain regions (5, 6). The prevalence of FSD caused by non-dermatophytes ranges from 0.8% to 65.8% (7, 8). In recent years, the incidence of non-dermatophyte infections has been steadily increasing, with studies in regions such as Thailand, Brazil, and Sri Lanka have reported that non-dermatophytes account for as much as 51.6%–68.2% of FSD (8, 9). Additionally, infections caused by Candida and Malassezia are on the rise, particularly among immunocompromised individuals, such as those with HIV/AIDS (10). The prevalence of FSD varies with age and gender (11–13). The incidence of FSD is highly variable due to factors such as climate, socioeconomic status, urban environment, migration, animal distribution, and cultural habits (1). Additionally, increasing research indicates that the incidence of chronic and persistent FSD has risen. This is not only associated with an increase in the prevalence of drug-resistant pathogens (14, 15) but may also be closely related to factors such as patients’ immune dysfunction (such as HIV, diabetes, systemic use of corticosteroids or immunosuppressants), natural immune deficiencies (such as CARD9 gene mutations), occupational exposure, climate change, and improvements in diagnostic tests and medical care (16).

FSD can significantly reduce patients’ quality of life, with disease severity being directly correlated with quality of life deterioration. This imposes a substantial burden on patients, their families, and national healthcare systems. Notably, skin and subcutaneous diseases are globally recognized as the fourth largest health problem, with FSD contributing the highest burden in most regions (2, 14). FSD can also have a substantial negative impact on patients' psychosocial health by reducing self-esteem, leading to embarrassment and social withdrawal (17). Additionally, the discomfort and inflammation associated with FSD may impair patients' ability to perform daily activities and even lead to disability (18). Severe FSD, if untreated or poorly managed, can result in secondary infections and significant morbidity, such as bacterial cellulitis and ulcers, thereby increasing mortality rates among patients with bacterial skin infections (19). Therefore, it is imperative to assess the burden caused by FSD to initiate targeted intervention campaigns globally.

In previous assessments, FSD was often discussed as part of the broader disease burden of Skin Diseases. Currently, only one study based on the GBD 2017 data has addressed FSD specifically (20). The study included only two indicators: prevalence and DALYs, analyzing the global burden of FSD. It utilized the DALYs metric to explore the current status of FSD across different age groups, genders, and high-burden countries. However, the study did not examine the changes in the burden of FSD over the past few decades. Furthermore, a comprehensive assessment of the burden of FSD based on GBD 2021 data remains largely unreported. Therefore, there is a need for a thorough description and analysis of the current global status of FSD and its trends over time. Based on the latest data from the 2021 GBD study, we evaluated the prevalence, incidence, and disability-adjusted life years (DALYs) for FSD at global, regional, and national levels from 1990 to 2021. Additionally, we analyzed the differences in the burden of FSD across various age groups and genders. By understanding the latest spatial distribution and temporal trends of FSD globally, we aim to inform policy-making, establish more rational and effective resource allocation, and implement disease prevention and control measures. This effort seeks to improve health disparities, enhance patients' quality of life, reduce avoidable healthcare costs, alleviate the disease burden, and potentially prompt action from policymakers.

## 2 Methods

### 2.1 Overview

The GBD study provides a comprehensive health assessment based on globally representative and reliable data sources. The GBD 2021 study estimated the disease burden of 371 diseases and injuries, as well as 88 risk factors, across 204 countries and territories. This dataset provides detailed estimates by gender (female and male) and 25 age groups (from birth to 95 years and older). All data from the GBD 2021 are freely accessible through the Global Health Data Exchange Query Tool (http://ghdx.healthdata.org/GBD-results-tool). These results include deaths, years of life lost (YLLs), years lived with disability (YLDs), DALYs, prevalence, incidence, and rates of change (21).

The Social Demographic Index (SDI) is a composite metric used to reflect the developmental status of a country or region. This index is calculated based on three factors: lag-distributed income per capita, average educational attainment, and the total fertility rate among women under the age of 25. Each country or region has a corresponding SDI value, ranging from 0 to 1, where a higher value indicates a better developmental status. In GBD 2021, the SDI value is multiplied by 100 to range from 0 to 100, and we used the 2021 SDI to categorize 204 countries into one of five SDI regions (21).

### 2.2 Definition of disease

Fungal diseases were included in the GBD 2021 cause group of skin and subcutaneous conditions and consisted of tinea capitis and a residual group of “any” other fungal disease. The ICD codes for FSD are B35–B36.9 in ICD-10 and 110–111.9 in ICD-9. The GBD 2021 classifies causes into four levels, ranging from Level 1 communicable, maternal, neonatal, and nutritional diseases to Level 4 latent tuberculosis infection. FSD is classified as a Level 3 cause in GBD 2021 (21).

### 2.3 Data analysis

Age standardization refers to the adjustment of age-specific disease rates in a study population to match the age distribution of a reference population, typically a standard population such as the World Standard Population (22). The data downloaded from the GBD database has already undergone age standardization and provides 95% UIs. Data cleaning and graph plotting in this study were performed using R software (version 4.3.1), with visualization generated using the ggplot2 package. Final data organization was completed in Excel, and editing was done in Adobe Illustrator software (version CS5).

## 3 Results

### 3.1 Escalating global burden of FSD

**Figure 1:**
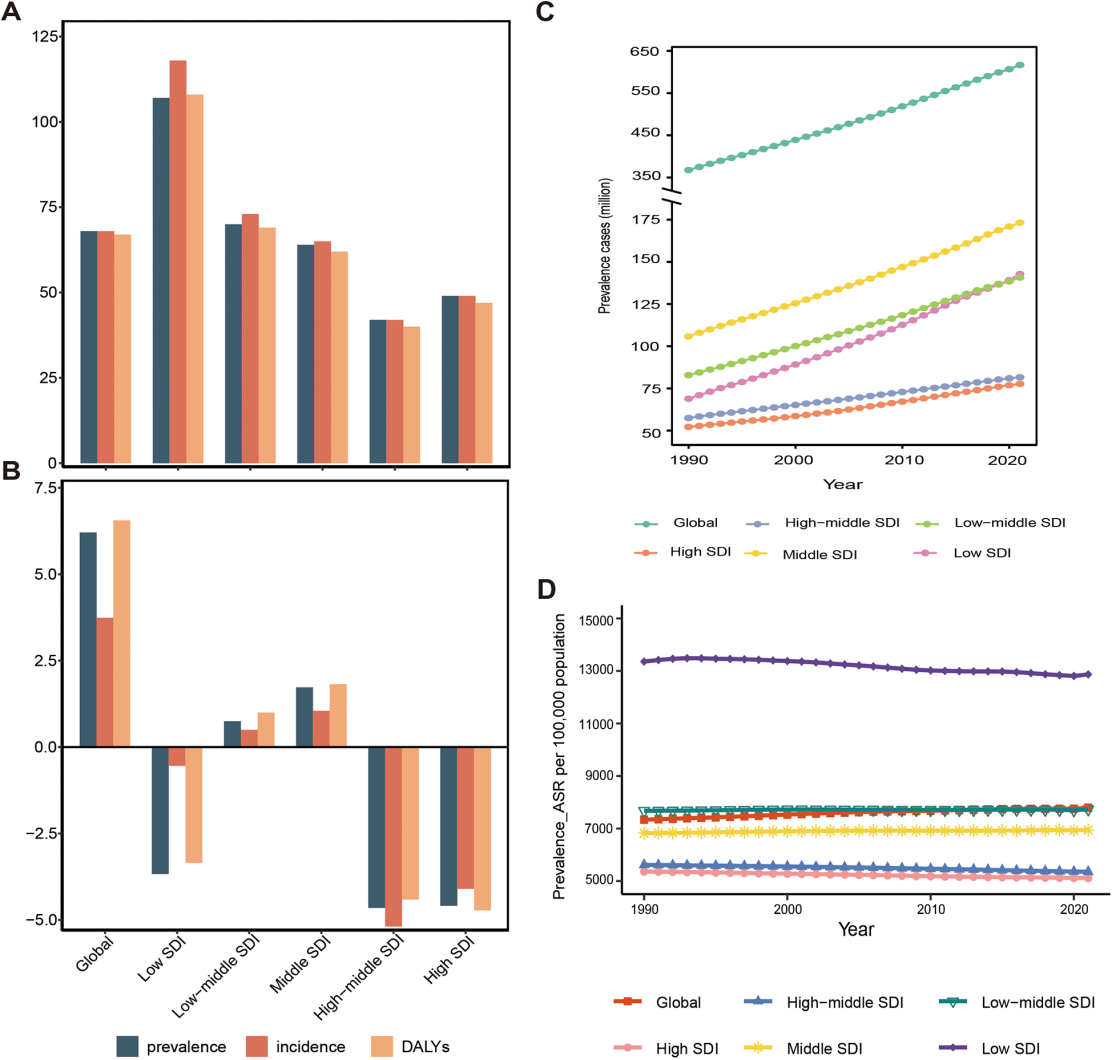
Temporal trend of fungal skin diseases in global and 5 territories from 1990 to 2021. **(A)** The percentage change in cases of prevalent, incident, and DALYs (%). **(B)** The percentage change in age-standardized prevalence rate (ASPR), age-standardized incidence rate (ASIR), and age-standardized DALYs rate (ASDR) (%). **(C)** The prevalent cases. **(D)** The age-standardized prevalence rate per 100,000 population. DALYs, disability-adjusted life years, SDI, social demographic index.

The percentage change in global FSD cases has significantly increased by approximately 68%. The prevalence cases rose from 367.6 million (95% UI: 331.5 to 409.3) in 1990 to 616.5 million (95% UI: 558.7 to 679.3) in 2021; the incidence cases increased from 1029.7 million (95% UI: 927.1 to 1,132.5) in 1990 to 1,729.2 million (95% UI:1,562.7 to 1,894.7) in 2021; and the number of DALYs escalated from 2,056.4 thousand (95% UI:842.5 to 4,249) in 1990 to 3,429.5 thousand (95% UI:1,407.9 to 7,044.3) in 2021. Notably, in 2021, the incidence cases of FSD were approximately three times the prevalence cases, indicating a higher number of new cases. The ASPR of FSD was 7,789.6 cases per 100,000, an increase of 6.21% since 1990. The ASIR was 21,668.4 cases per 100,000, an increase of 3.74% since 1990. The age-standardized DALYs rate(ASDR) was 43.4 cases per 100,000, an increase of 6.56% since 1990 (Table 1; Figure 1; Supplementary Tables S1, S2; Supplementary Figures S1–S7). Therefore, the global burden of FSD has increased over the past 32 years.

**Table 1:** Comparative analysis of prevalent cases and age-standardized rate (ASR) changes in fungal skin diseases by region from 1990 to 2021.

Location	No. (millions)		Percentage change(%)	ASRs per 100,000		Percentage change in the ASRs per 100,000 (%)
	1990 (95%UI)	2021 (95%UI)		1990 (95%UI)	2021 (95%UI)	
Global	367.6 (331.5, 409.3)	616.5 (558.7, 679.3)	68	7334.2 (6652.5, 8063.7)	7,789.6 (7,059.3, 8,583.5)	6.21 (5.34, 7.18)
Low SDI	68.9 (59, 79.5)	142.8 (123.1, 163.3)	107	13,356.5 (11,915.9, 14,866.9)	12,866.9 (11,521.3, 14,298.5)	−3.67 (−4.92, −2.4)
Low-middle SDI	82.9 (72.4, 95.5)	140.7 (125.7, 158)	70	7,673.4 (6,884.3, 8,556.4)	7,731.2 (6,958.7, 8,612.3)	0.75 (−0.19, 1.61)
Middle SDI	105.8 (94.8, 118.2)	173.2 (156, 192.8)	64	6,823.6 (6,184.9, 7,537.8)	6,941.8 (6,276.7, 7,692.3)	1.73 (1.07, 2.37)
High-middle SDI	57.5 (52.1, 63.4)	81.6 (74.4, 90.3)	42	5,615.4 (5,120.4, 6,196.2)	5,354.5 (4,863.2, 5,911.8)	−4.65 (−5.42, −3.78)
High SDI	52.2 (48.1, 56.9)	77.7 (72, 85.2)	49	5,363.6 (4,940.2, 5,831.1)	5,117.3 (4,725.7, 5,561.4)	−4.59 (−5.19, −3.99)
High-income Asia Pacific	11 (9.9, 12.2)	18.1 (16.4, 20.3)	65	6,018.4 (5,452.1, 6,661.8)	5,921.2 (5,359.8, 6,562.5)	−1.62 (−2.23, −1.03)
High-income North America	7.9 (7.5, 8.2)	12.2 (11.8, 12.8)	54	2,507.1 (2,396.6, 2,619.9)	2,506.9 (2,407, 2,606.1)	−0.01 (−1.11, 0.98)
Western Europe	34.9 (31.8, 38.7)	47.6 (43.3, 53)	36	7,671.9 (6,968.8, 8,455.5)	7,634.4 (6,923.3, 8,402.4)	−0.49 (−0.93, −0.13)
Australasia	1.9 (1.8, 2)	3.6 (3.4, 3.7)	89	8,724.9 (8,310.8, 9,178.8)	8,674.3 (8,253.1, 9,133)	−0.58 (−0.83, −0.37)
Andean Latin America	4 (3.5, 4.5)	7.8 (7, 8.7)	95	12,382.3 (11,123.2, 13,682.8)	12,185.3 (10,928.5, 13,484.3)	−1.59 (−2.08, −1.15)
Tropical Latin America	11.3 (10.2, 12.7)	20.4 (18.5, 22.6)	81	8,710.4 (7,904.8, 9,581.8)	8,597.1 (7,778.7, 9,470.5)	−1.3 (−1.72, −0.96)
Central Latin America	7.4 (6.7, 8.2)	14.3 (13, 15.8)	93	5,414.1 (4,938.4, 5,985.3)	5,684.2 (5,172.9, 6,293.5)	4.99 (2.69, 7.25)
Southern Latin America	2.8 (2.6, 3.1)	4.5 (4, 4.9)	61	5,936.3 (5,383.2, 6,543.3)	5,844.9 (5,296.9, 6,447.1)	−1.54 (−2.05, −1.12)
Caribbean	2.7 (2.4, 3)	4.2 (3.8, 4.7)	56	8,515.7 (7,718.7, 9,449.9)	8,490.9 (7,688.7, 9,420.8)	−0.29 (−0.57, −0.06)
Central Europe	7.4 (6.7, 8.2)	8.8 (8, 9.9)	19	5,550.4 (5,049.3, 6,168.3)	5,547.7 (5,046.8, 6,164.8)	−0.05 (−0.22, 0.11)
Eastern Europe	13.7 (12.5, 15.3)	14.9 (13.6, 16.8)	9	5,641.7 (5,135.6, 6,281.9)	5,640.6 (5,132.7, 6,279.9)	−0.02 (−0.2, 0.15)
Central Asia	3.2 (2.9, 3.6)	4.8 (4.4, 5.4)	50	5,504.3 (4,997.2, 6,131.3)	5,502.5 (4,989.4, 6,130.4)	−0.03 (−0.23, 0.17)
North Africa and Middle East	8.8 (8.1, 9.7)	17.9 (16.2, 19.7)	103	3,458.5 (3,165.9, 3,824.7)	3,311.4 (3,029, 3,657.6)	−4.25 (−5.04, −3.57)
South Asia	70.6 (60.2, 82.6)	112.1 (99, 126.4)	59	6,758.7 (5,942,7, 613.9)	6,425.7 (5,692.9, 7,177.1)	−4.93 (−6.86, −3.31)
Southeast Asia	46.1 (40.6, 51.8)	77 (68.1, 86.6)	67	10,927.3 (9,742.3, 12,188.3)	10,853.5 (9,663.9, 12,128.5)	−0.68 (−0.98, −0.41)
East Asia	51.9 (46.3, 58.2)	71.8 (64.8, 79.8)	38	4,557.2 (4,102.1, 5,060)	4,346.9 (3,907.3, 4,830.3)	−4.61 (−5.86, −3.59)
Oceania	0.3 (0.3, 0.4)	0.7 (0.7, 0.8)	133	5,974.6 (5,337.9 ,6,667.7)	5,973 (5,353.8, 6,666)	−0.03 (−0.59, 0.54)
Western Sub-Saharan Africa	32.8 (28.4, 37.5)	81.8 (71.3, 93.1)	149	17,742.6 (15,939.6, 19,747.1)	17,516.4 (15,769.2, 19,474.2)	−1.27 (−2.02, −0.65)
Eastern Sub-Saharan Africa	35.5 (30.3, 41)	68.9 (59.6, 79)	94	17,634.1 (15,740.2, 19,731.7)	16,407.6 (14,685.9, 18,312.7)	−6.95 (−8.69, −5.22)
Central Sub-Saharan Africa	8.6 (6.6, 10.9)	17.7 (13.8, 21.9)	106	13,775.2 (11,384.5, 16,448.7)	12,157.2 (10,215.6, 14,241.1)	−11.75 (−15.93, −7.24)
Southern Sub-Saharan Africa	4.7 (4.1, 5.3)	7.5 (6.6, 8.4)	60	10,199.1 (9,121.7, 11,313.6)	10,149 (9,087.1, 11,254)	−0.49 (−1.17, 0.04)

ASRs, age-standardized rates.

### 3.2 Substantial discrepancies in FSD between developing and developed regions

The burden of FSD and its trends show significant variations across the 5 SDI regions. Overall, in high and high-middle SDI, both the cases and the percentage increase of FSD cases are significantly lower compared to middle, low-middle, and low SDI regions. For example, in 2021, the prevalence numbers of FSD from high SDI to low SDI were 77.7 million, 81.6 million, 173.2 million, 140.7 million, and 142.8 million, respectively. The percentage increases since 1990 in these regions were 49%, 42%, 64%, 70%, and 107%, respectively. In 2021, with the improvement of SDI, the ASPR, ASIR, and ASDR of FSD gradually decreased. For example, the ASPR per 100,000 decreased from 12,866.9 (95% UI: 11,521.3–14,298.5) in low SDI to 5,117.3 (95% UI: 4,725.7 to 5,561.4) in high SDI. Over the past 32 years, the middle and low-middle SDI has experienced an increasing trend in the percentage change of ASPR, ASIR, and ASDR of FSD. In contrast, low, high-middle, and high-SDI regions have shown a declining trend, with low-SDI regions exhibiting a slower decline compared to high-middle and high-SDI regions. For example, the percentage change in ASDR has decreased by 3.35%, 4.41%, and 4.72% in these three regions, respectively (Table 1; Figure 1; Supplementary Tables S1, S2; Supplementary Figures S1–S7). In summary, the disease burden of FSD is higher in developing regions than in developed regions, and this gap may further widen in the future. Notably, in 2021, the middle SDI had the highest absolute cases of FSD among the 5 SDI regions, with prevalence, incidence, and DALY cases of 173.2 million, 503.3 million, and 963.1 thousand, respectively. These cases accounted for approximately 30% of the global total. Additionally, the middle SDI experienced the highest percentage increases in ASPR, ASIR, and ASDR, with 1.73%, 1.05%, and 1.82%, respectively. As a result, the middle SDI region bears the heaviest burden of FSD (Table 1; Figure 1; Supplementary Tables S1, S2; Supplementary Figures S1–S7).

Among the 21 regions, the percentage increase in FSD cases has varied between 8% and 150% since 1990. In 2021, the highest cases of prevalence, incidence, and DALYs of FSD were observed in South Asia, with 112.1 million, 340.9 million, and 624.8 thousand, respectively. The highest ASPR, ASIR, and ASDR of FSD were reported in Western Sub-Saharan Africa, with 17,516.4 (95% UI: 15,769.2 to 19,474.2), 39,705.2 (95% UI: 35,574.6 to 43,841), 97.7 (95% UI: 40.1 to 202.8) per 100,000, respectively. Notably, this region also showed relatively high FSD prevalence, incidence, and DALYs cases, with 81.8 million, 179.4 million, and 462.3 thousand, representing percentage increases of 149%, 151%, and 150% compared to 1990, highlighting the heavy burden of FSD in this region. Over the past 32 years, most regions have shown a downward trend in the percentage change of ASPR, ASIR, and ASDR related to FSD, with ranges of ASPR (−11.75% to 4.99%), ASIR (−3.79% to 4.3%), and ASDR (−11.59% to 5%). Only a few regions showed an upward trend (Table 1; Supplementary Tables S1, S2; Supplementary Figures S8–S12).

**Figure 2:**
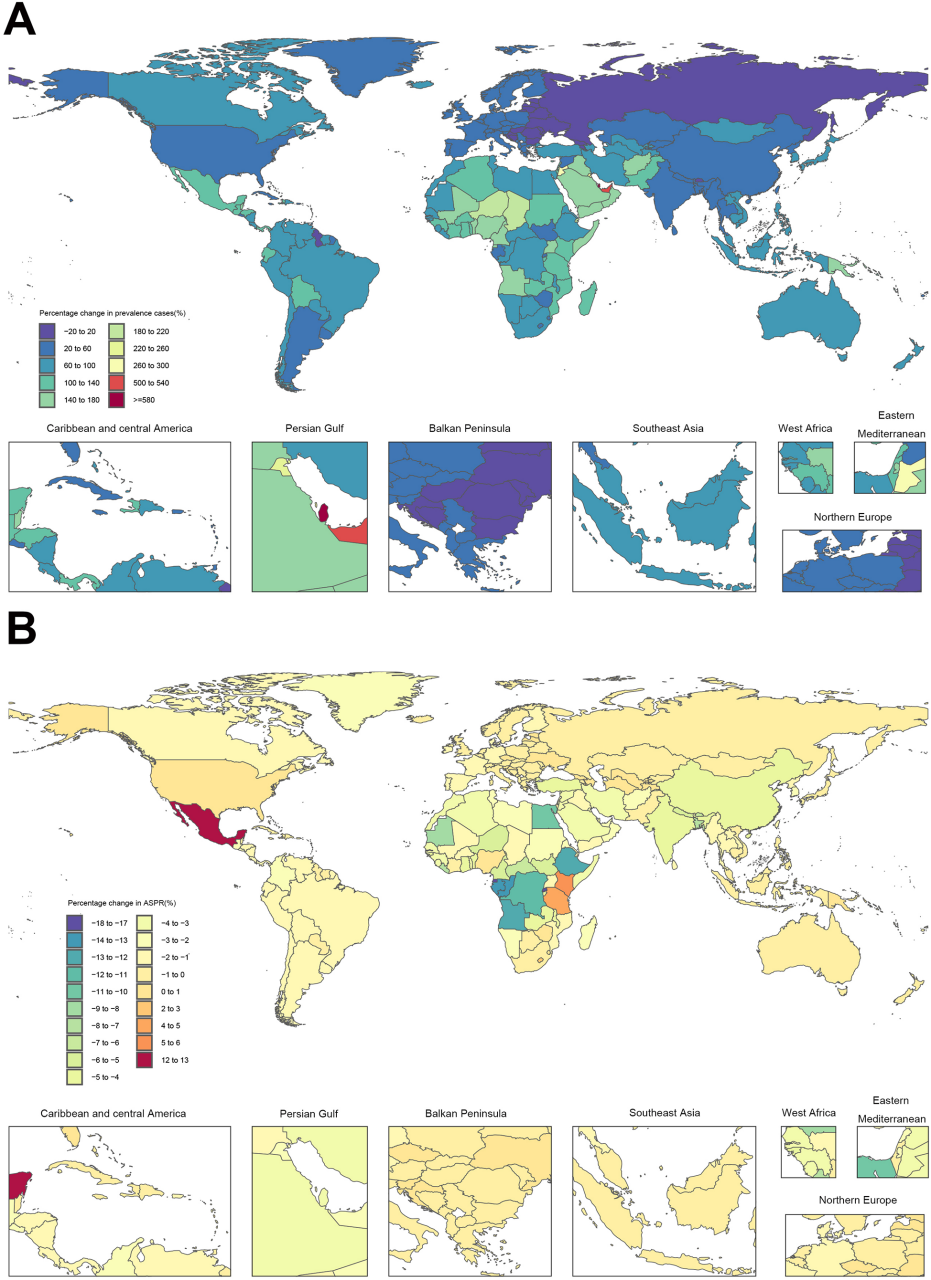
Temporal trend of fungal skin diseases globally from 1990 to 2021. **(A)** The percentage change in prevalent cases across 204 countries. **(B)** The percentage change in age-standardized prevalence rate (ASPR) across 204 countries.

At the national level, from 1990 to 2021, the majority of countries globally exhibited a percentage increased trend in the cases of FSD, with only a few countries showing a downward trend. The percentage range spanned from −23% to 626%. The countries with the highest percentage increases of FSD-related cases were Qatar and the United Arab Emirates, with increases ranging from approximately 500% to 630%, while Georgia saw the largest decrease, which was around 20%. In 2021, the countries with the highest cases of FSD were predominantly developing nations, specifically India and China. For example, the prevalence cases of FSD were 87,230,762 and 69,601,875, respectively. However, the percentage increase in the cases was relatively low at 56% and 38%, respectively. Both India and China showed a downward trend in the percentage change of FSD-related ASPR, ASIR, and ASDR since 1990. For example, the percentage change in ASDR was −4.8% (95% UI: −6.9 to −3.1) for India and −4.8% (95% UI: −6.1 to −3.7) for China. Over the past 32 years, approximately 88% of countries worldwide have experienced a decline in age-standardized rates (ASRs) of FSD. This decline is most notable in developing countries, with Rwanda showing the largest percentage decrease in ASPR at −17.5% (95% UI: −20.4, −13.4). This suggests significant improvements in healthcare and health awareness in these countries. In contrast, Mexico presents the highest percentage increases in ASPR, ASIR, and ASDR, rising by 12.2%, 10.4%, and 12.3%, respectively, highlighting the growing burden of FSD in the country (Figure 2; Supplementary Tables S3–S5; Supplementary Figures S13–S16).

### 3.3 Global male FSD burden higher, especially in developing nations

**Figure 3:**
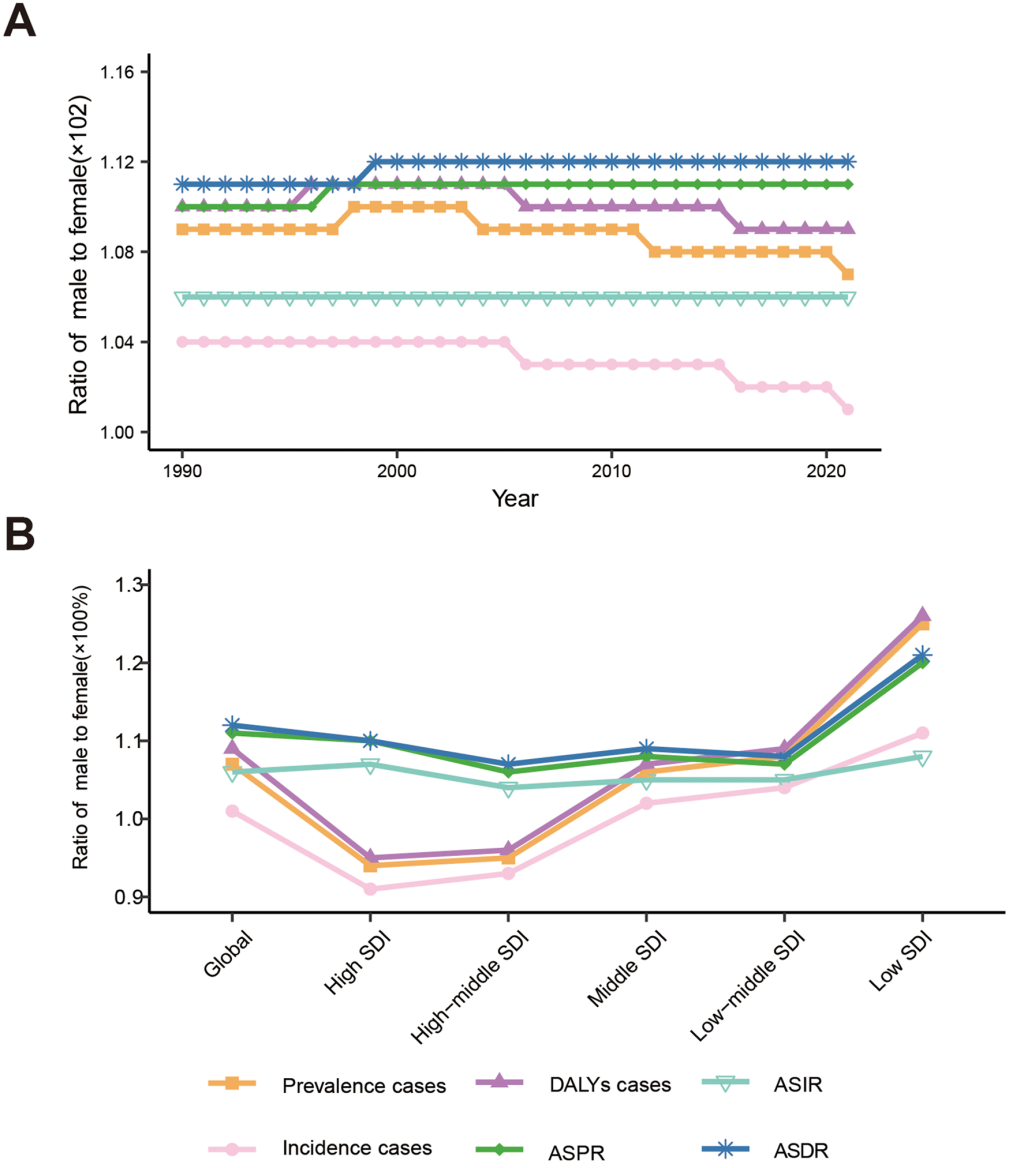
Male-to-female ratios of fungal skin diseases: case number, age-standardized prevalence rate (ASPR), age-standardized incidence rate (ASIR), and age-standardized DALYs rate (ASDR). **(A)** Global from 1990 to 2021. **(B)** Global and 5 SDI territories in 2021. DALYs, disability-adjusted life years, SDI, social demographic index.

From 1990 to 2021, the global male-to-female ratios for FSD prevalence, incidence, and DALY cases consistently exceeded 1, approximately at 1.09, 1.03, and 1.10, respectively. Similarly, the male-to-female ratios of ASPR, ASIR, and ASDR for FSD were also greater than 1, at approximately 1.11, 1.06, and 1.11, respectively. These findings indicate that the global burden of FSD is higher in males than females and has remained relatively stable over time. This suggests that the gender disparities in FSD burden are unlikely to worsen in the future (Figure 3A; Supplementary Table S6).

In 2021, across the 5 SDI regions, there was a gradual decrease in the male-to-female ratios for FSD cases of prevalence, incidence, and DALYs as the SDI improved. For instance, the male-to-female ratio for prevalence cases dropped from 1.25 in Low SDI to 0.94 in High SDI. In low, low-middle, and middle SDI regions, these ratios were greater than 1, indicating higher cases in males compared to females. Conversely, in the high-middle and high SDI regions, the ratios were less than 1, signifying a higher case in females than males. However, in 2021, the male-to-female ratios for FSD ASPR, ASIR, and ASDR were all greater than 1, indicating higher ASR in males compared to females. Notably, these ratios were significantly higher in the Low SDI compared to the high SDI regions. For instance, the global male-to-female ratio for FSD-related ASDR was 1.21 in the low SDI, while it was 1.10 in the High SDI. In low-middle, middle, and high-middle SDI regions, the ratio was relatively closer, at approximately 1.08. In conclusion, the burden of FSD is higher in males than females in developing regions, and this disparity is more significant in developing countries compared to developed countries (Figure 3B; Supplementary Table S7).

### 3.4 Gender disparity in FSD cases reverses in elderly age groups, with females bearing higher burdens

**Figure 4:**
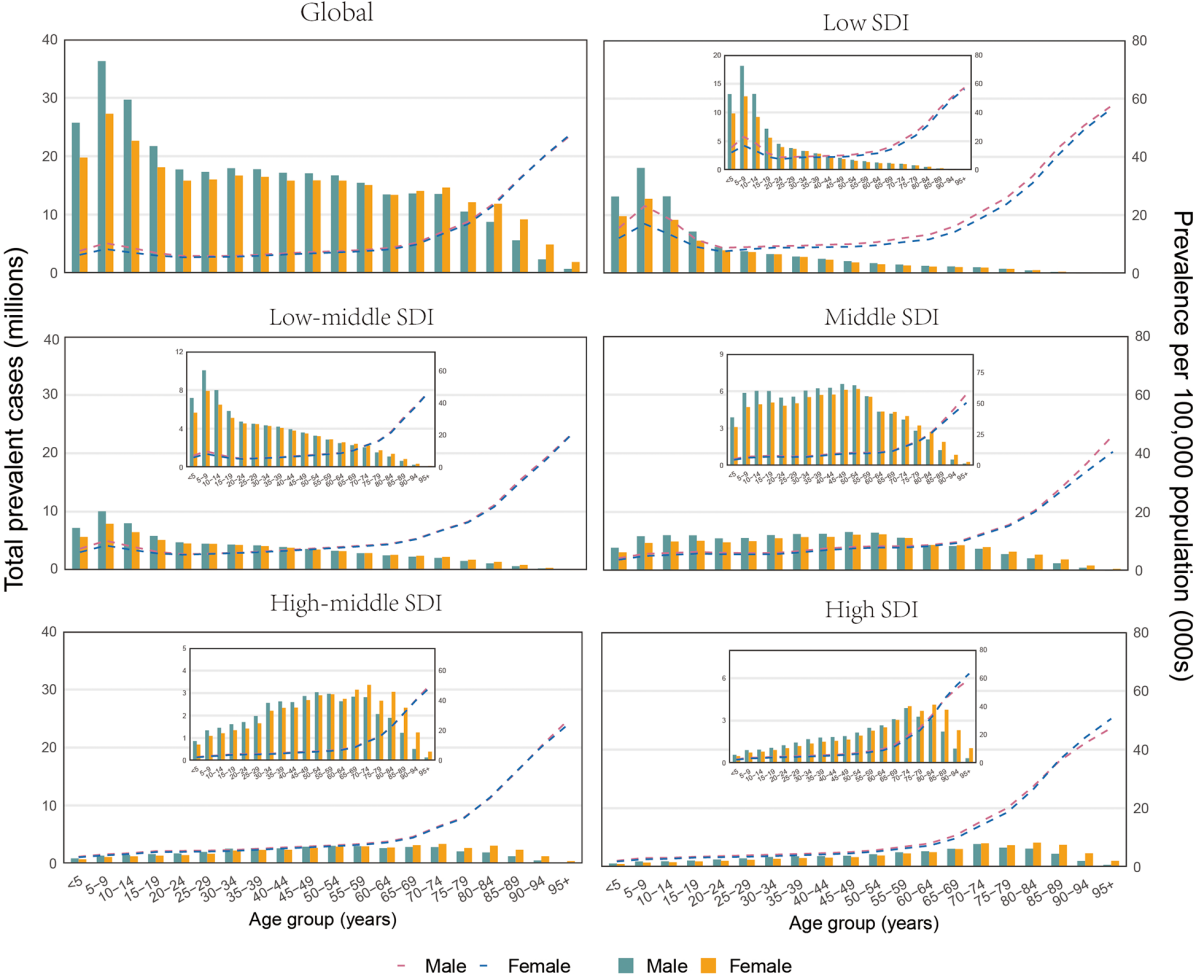
Global and 5 SDI territories: the number of prevalent cases and prevalence of fungal skin diseases per 100,000 population by age and sex in 2021. The Bar Chart Represents the number of prevalent cases, While the dashed line chart represents the age-standardized prevalence rate. SDI, social demographic index.

In 2021, the highest global FSD cases were observed in the 5–9 age group. For example, The prevalence of cases reached as high as 63,559,849.13. Across the 5 SDI regions, as the SDI improved, the age group at which FSD-related cases peaked progressively shifted towards older ages. For example, in low and low-middle SDI regions, the peak of FSD prevalence cases was observed in the 5–9 age group, with 30,901,170.87 and 17,959,743.78 cases, respectively. In the middle SDI, the peak occurred in the 45–49 age group, with 12,680,038.76 cases. In high-middle and high SDI regions, the peak cases were in the 70–74 age group, with 6,178,684.812 and 7,900,182.859 cases, respectively. In contrast, the trends for FSD-related ASPR, ASIR, and ASDR globally and across the 5 SDI regions were nearly identical, remaining relatively stable before the age of 60, after which there was a sharp increase starting in the 60–64 age group (Figure 4; Supplementary Figures S17–S28).

In 2021, a consistent trend was observed in FSD cases of prevalence, incidence, and DALYs globally and across the 5 SDI regions. Initially, the cases of FSD in males exceeded that in females, but after a certain age group, the cases in females surpassed that of males. For instance, globally, the FSD cases in males were higher than in females until the age of 65, at which point the female cases began to exceed those of males in the 65–69 age group. Across the 5 SDI regions, the age at which this reversal occurred varied with improvements in SDI. Across the Low SDI to High SDI regions, the age at which the reversal in FSD-related prevalence cases occurred was 80–84 years, 55–59 years, 60–64 years, 60–64 years, and 70–74 years, respectively. This indicates that a reversal of FSD-related cases between males and females occurs in the elderly age groups, with females bearing a relatively higher burden in the older age groups(Figure 4; Supplementary Figures S17–S28).

### 3.5 Correlation between FSD disease burden and SDI

**Figure 5:**
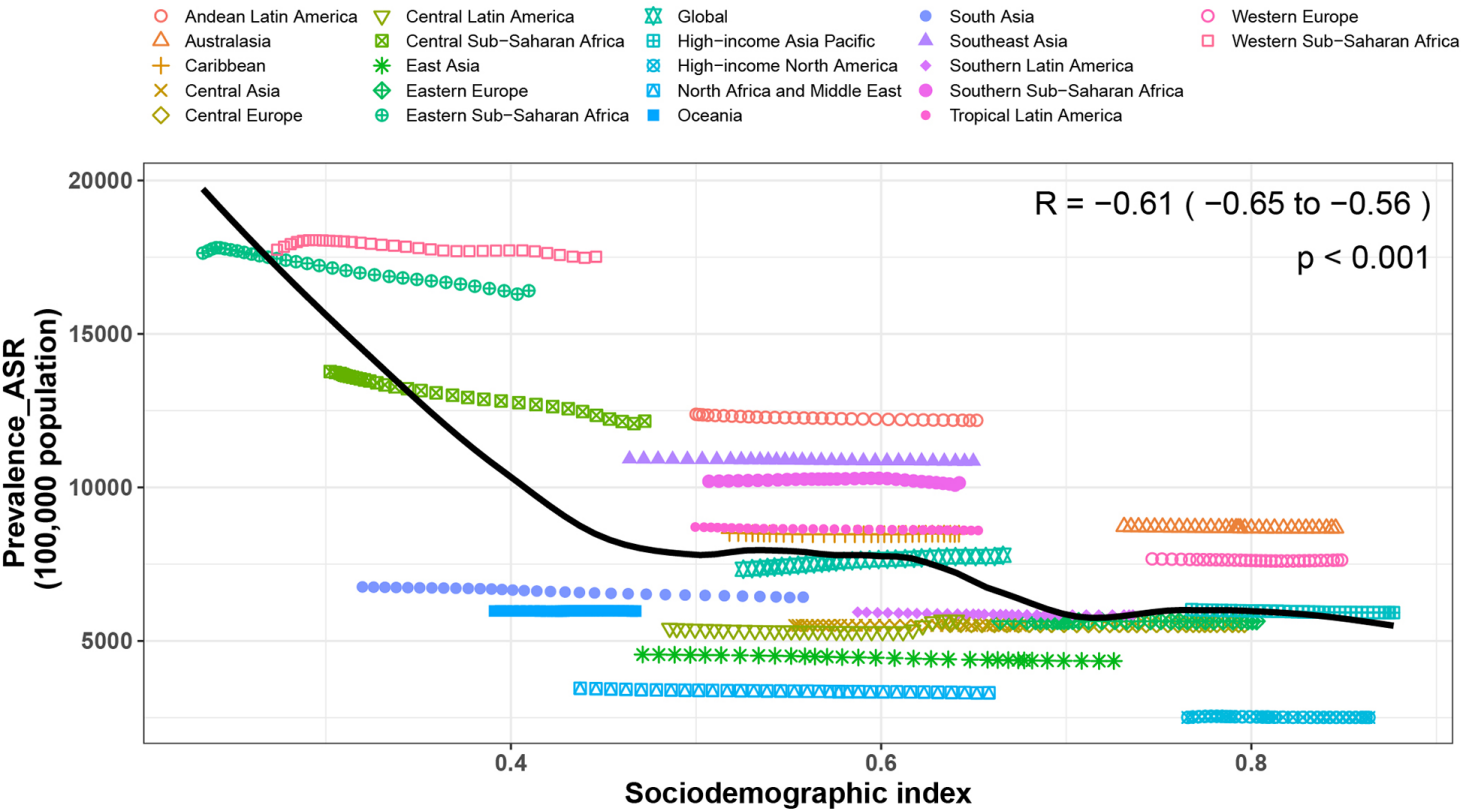
Associations between the SDI and prevalence rates per 100,000 population of fungal skin diseases across 21 GBD regions and globally. SDI, social demographic index, GBD, global burden of disease.

In 2021, there was a negative correlation between FSD-related ASPR, ASIR, ASDR, and SDI. As economic conditions improve, the health burden of FSD shows a declining trend, indicating a reduction in the FSD burden with socioeconomic progress. Globally, the FSD burden was generally in line with expected levels. Among the 21 global regions, when the SDI was less than 0.45, the ASPR, ASIR, and ASDR for FSD showed a linear decline with increasing SDI. This trend remained relatively stable when the SDI was approximately 0.45–0.6 and subsequently exhibited a gradual decline. Regions such as Andean Latin America and Western Sub-Saharan Africa experienced burdens exceeding expectations, whereas regions like South Asia and East Asia had lower-than-expected burdens. On a national level, the burden of FSD in countries such as Ethiopia and Nigeria far exceeds expectations, while in countries like Afghanistan and China, it was significantly lower than expected (Figure 5; Supplementary Figures S29–S33).

## 4 Discussion

Our research findings indicate that over the past 32 years, the global cases of prevalence, incidence, and DALYs of FSD have significantly increased by approximately 68%. This growth may be associated with a 45% increase in the global population, as well as improvements in public health systems and enhanced information reporting capabilities (23). Concurrently, the ASPR, ASIR, and ASDR of FSD are also showing an upward trend, indicating a worsening burden of FSD-related diseases globally. This trend may be attributed to the limited variety of antifungal drugs available (24) and the increasing resistance of various dermatophytes to these treatments (25), leading to a significant rise in globally treatment-resistant FSD. Notably, in 2021, the number of incidents of FSD is approximately three times higher than the number of prevalent cases, indicating a surge in new cases. We hypothesize that this increase may be linked to the global COVID-19 pandemic. Studies have shown that COVID-19 is a new risk factor for fungal infections, as its impact on the human immune system and treatments for COVID-19 can weaken the body's defences against fungi (26).

The study results indicate that in 2021, the number of FSD-related cases, ASPR, ASIR, and ASDR in developing regions was higher than those in developed regions. Research has shown that developed regions have a greater number of social determinants of health (SDOH), which are associated with an increased risk of FSD (27). Further analysis revealed that the middle SDI regions had the highest cases of FSD, accounting for 30% of the global. Over the past 32 years, the increase in the number of FSD-related cases in developing regions has exceeded that in developed regions. Overall, the burden of FSD is higher in developing regions, particularly in middle SDI regions, and this disparity is likely to widen in the future. Several factors may contribute to this phenomenon: Firstly, developing regions have large population bases and often face poorer socioeconomic conditions, which create favourable conditions for the transmission of FSD pathogens. Crowded living conditions facilitate close and prolonged contact between individuals, increased exposure to animal vectors, suboptimal hygiene, poor environmental sanitation, and a lack of public awareness about FSD pathogens, all of which can elevate infection risk (28, 29). Secondly, dermatophytes thrive in warm and humid environments, making them more common in tropical and subtropical regions (30), where many developing countries are located. Thirdly, in high SDI, robust healthcare systems are typically in place. Conversely, healthcare infrastructure in developing countries is often inadequate, and access to medical care is limited, which can result in misdiagnosis or delayed diagnosis of infections (31). Moreover, with changes in the socioeconomic conditions of these regions, there is an increase in reported triggering factors and disease diagnoses of FSD. The ongoing development of tourism and immigration hotspots also contributes to the epidemiological shift of FSD, further complicating its treatment (32, 33). Prolonged wearing of enclosed clothing can exacerbate dermatophyte infections, and clothing as a vector for dermatophytes poses a significant health risk. For example, a study found that FSD pathogens were isolated from clothing in the thriving second-hand clothing markets in Kenya, and as the markets become increasingly popular in certain African countries, potentially contributing to the rise of FSD in these regions (34). Therefore, it is necessary to devise country-specific strategies to reduce the burden of FSD. These strategies may include enhancing public health education, strengthening epidemiological surveillance, improving healthcare systems, advancing diagnostic technologies, and promoting interdisciplinary research to develop more effective prevention and treatment methods.

In 2021, the absolute highest number of FSD-related cases was reported in South Asia, while the highest ASPR was observed in Western Sub-Saharan Africa. Simultaneously, this region also had relatively high cases and showed a relatively high percentage change, indicating a heavy burden of FSD in these regions. This aligns with previous research (20), which attributes the high burden to the persistent prevalence of FSD in these areas, as well as to factors such as medical service levels, economic conditions, climate, and cultural background. Furthermore, studies have indicated that global warming may increase the incidence and transmission of skin fungal infections (35, 36). These findings underscore the urgency for governments in these regions to implement measures such as enhancing health education, providing accessible medical services, and promoting healthy lifestyles to mitigate this adverse trend. Our study reveals significant differences in the percentage change in FSD-related cases over the past 32 years across different countries. Countries in high SDI regions especially exhibit a marked polarization, with disparities up to 33-fold. India and China, the world's two most populous countries, had the highest number of FSD-related cases in 2021, accounting for 18% of the global population each. Although a decrease in ASPR, ASIR, and ASDR has been shown in India and China since 1990, these countries still account for the highest absolute FSD cases. Recent studies have reported a significant increase in the prevalence of dermatophytosis in India over the past decade, with prevalence rates ranging from 6.0% to 61.5%. Among these cases, 5%–10% of new cases of dermatophytosis have been identified as chronic and recurrent (3, 5). Additionally, research indicates an increasing number of reports of disseminated trichosporonosis worldwide, including in India (37). Furthermore, a notable epidemiological shift from T. rubrum to Trichophyton (T.) indotineae has been observed in India (38). Tinea capitis, one of the most common FSDs worldwide, primarily affects preadolescent children, especially boys. In 2021, China had 250 million children under the age of 14 (12, 39). Conversely, the declining trend in a few countries may reflect the positive impact of healthcare and public health management efforts in raising public health awareness, enhancing preventive measures, and improving disease outcomes (33). Ongoing monitoring of trends in these countries could help inform global strategies to alleviate the FSD burden.

Consistent with previous reports (20), the global burden of FSD, especially in developing countries, is higher among males than females and remains relatively stable, particularly in younger age groups. However, this trend reverses in older age groups (>65 years). Among the 5 SDI regions, the number of FSD-related cases is higher in males than females in low, low-middle, and middle SDI regions, whereas it is higher in females in high-middle and high SDI regions. In terms of age, consistent with previous research findings, the number of FSD-related prevalence, incidence, and DALY cases peak at ages 5–9 globally, while the ASR of these indicators shows a sharp increase in the 60–64 age group. The age groups at which FSD peaks cases in low SDI and low-middle SDI, middle SDI, high-middle SDI, and high SDI regions are 5–9 years, 45–49 years, and 70–74 years, respectively. This phenomenon is primarily due to poor personal hygiene, the use of local barbers, and close contact with playmates and siblings in younger age groups, increasing the risk of disease transmission. Elderly individuals are more susceptible to fungal infections due to factors such as declining physical function, decreased immune function, and multiple chronic comorbidities (40, 41). Furthermore, males in developing regions, as the primary household labour force, are more prone to occupational exposure. However, with age, the risk of infection decreases as men are less likely to engage in fungus-prone occupations (42). Conversely, women are more likely to keep pets, increasing the risk of zoonotic dermatophyte infections (43). With the improvement of SDI, the peak age for FSD-related cases has gradually shifted toward older age groups. This shift may also be related to females having a longer life expectancy than males and the ageing population in developed countries. There is a significant negative correlation between the ASR of FSD and SDI. In countries with lower SDI, the ASR of FSD has declined more markedly, possibly attributed to the increasing attention to the diagnosis, prevention, and treatment of FSD in these regions.

FSD not only occurs as a primary disease but also serves as a source of infection, transmitted directly or indirectly to close contacts through contaminated domestic or public environments, thereby further increasing the disease burden. In recent years, the increasing number of immunocompromised patients and the globalization of travel have led to rising incidence rates of FSD outside endemic regions. Concurrently, the global rise and spread of antifungal resistance, particularly among dermatophytes showing resistance to terbinafine, have caused significant healthcare challenges worldwide (44). On the other hand, the high failure rate of antifungal therapy poses greater challenges for the treatment of non-dermatophyte fungal infections (45). Furthermore, the ongoing COVID-19 pandemic has further exacerbated the incidence and treatment challenges of FSD. Therefore, enhanced monitoring and diagnosis of FSD, as well as increased public awareness and education on FSD and antifungal resistance, are essential. Public health intervention measures should promptly adapt to changes in factors and patterns of disease analysis and promote research and innovation in FSD prevention, diagnosis, treatment, and intervention measures. Efforts should also be made to promote research and development of novel antifungal drugs, and based on the latest distribution characteristics in various countries and regions, formulate targeted and effective comprehensive control strategies for FSD, striving to reduce the significant burden of FSD.

This study has several limitations. Firstly, the data primarily originate from disease reports and healthcare institutions, which may result in the inclusion of cases predominantly focusing on specific types of skin fungal infections. Additionally, the lack of more detailed clinical information may lead to incomplete or inaccurate data, potentially introducing bias in the burden assessment. Secondly, the data obtained from the GBD study heavily relies on statistical modelling methods, particularly in countries lacking original data, which may influence the accuracy of the results. Lastly, the assessment of FSD burden is complex, and the definition of disability weights remains quite superficial, making reliable “measurability” of disabilities very challenging. Additionally, the lack of mandatory reporting for fungal diseases, poor diagnostic test performance, and limited diagnostic capabilities of clinicians in certain regions may impact the reported global incidence of FSD. Therefore, when analyzing the burden of FSD using the GBD database, it is crucial to interpret the results with caution. It is recommended that other data sources and field surveys be integrated for a more accurate and comprehensive assessment.

## 5 Conclusions

Overall, the global burden of FSD has significantly increased from 1990 to 2021. The burden of FSD is negatively correlated with the SDI across regions and specific countries. Developing countries, particularly in middle SDI regions, exhibited a significantly higher burden of FSD compared to developed countries. While most countries experienced a declining trend in disease burden, a few countries, such as Mexico, are experiencing an upward trend in burden. The global burden of FSD is higher in males than in females, but this trend reverses in older age groups (>65 years). Additionally, as SDI improved, the age group at which FSD-related cases peak is gradually shifting towards older age groups. These findings highlight the necessity of developing targeted prevention and treatment strategies and rationally allocating healthcare resources to address the considerable variations in FSD burden across different geographic locations, genders, and populations.

## Data Availability

The original contributions presented in the study are included in the article/Supplementary Material, further inquiries can be directed to the corresponding authors.
