# Supplementary material for: A systematic analysis of the global, regional, and national burden of fungal skin diseases from 1990 to 2021
Source: Front Epidemiol. 2024 Dec 16;4:1489148. doi: 10.3389/fepid.2024.1489148 (PMC11686433; doi:10.3389/fepid.2024.1489148)
Supplement: Supplementary file 1 [file Datasheet1.zip › FSD supplementary material-20241205-v2/Online supplementary figure.docx]

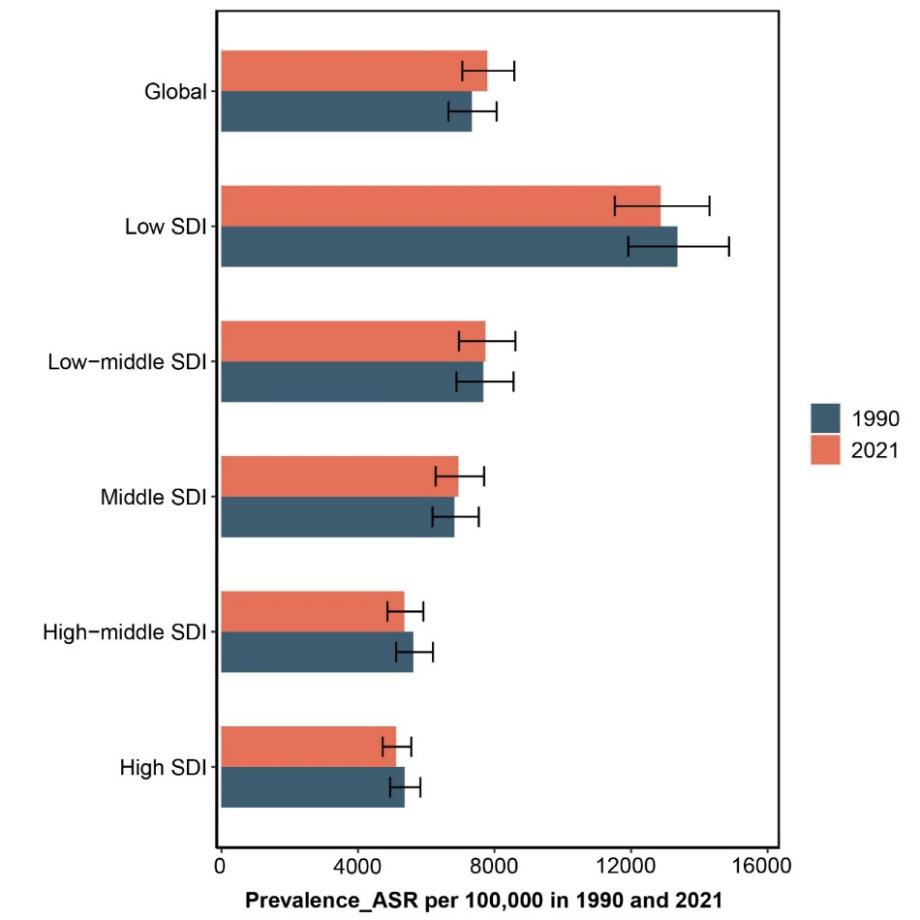


**Figure S1.** Age-standardized prevalence rates of fungal skin diseases in 1990 and 2021 for the global and 5 SDI territories. SDI-socio demographic index.


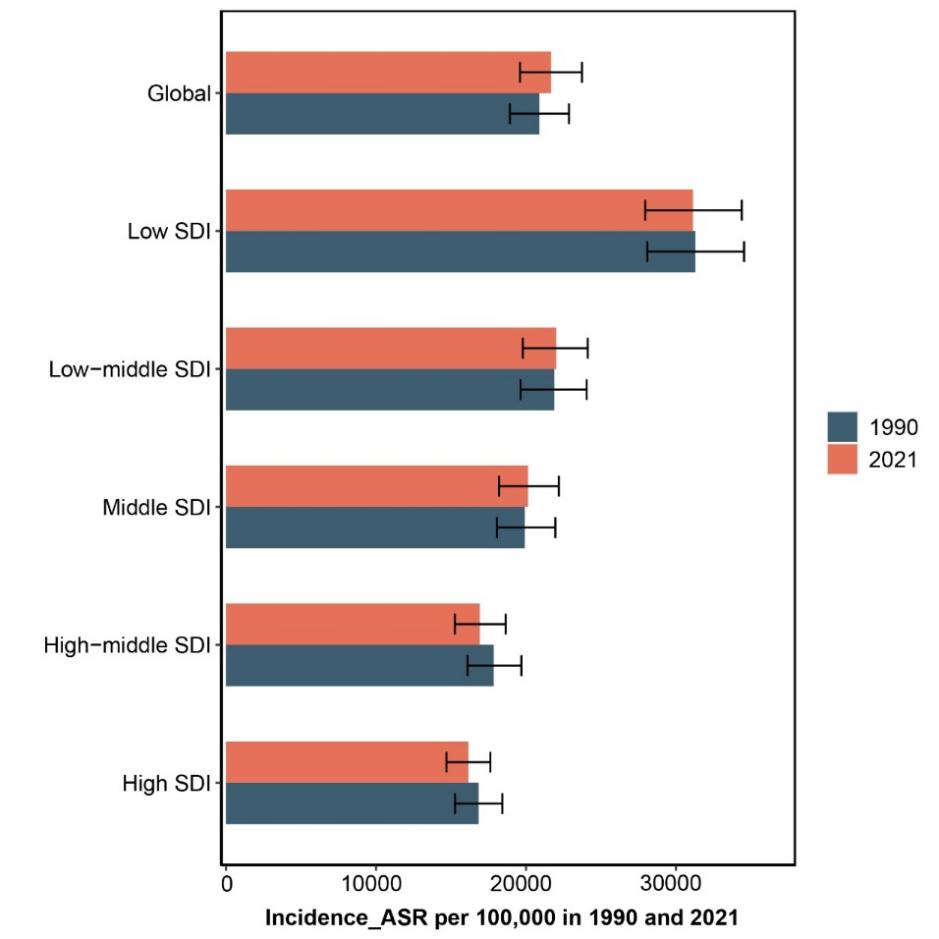


**Figure S2.** Age-standardized incidence rates of fungal skin diseases in 1990 and 2021 for the global and 5 SDI territories. SDI-socio demographic index.


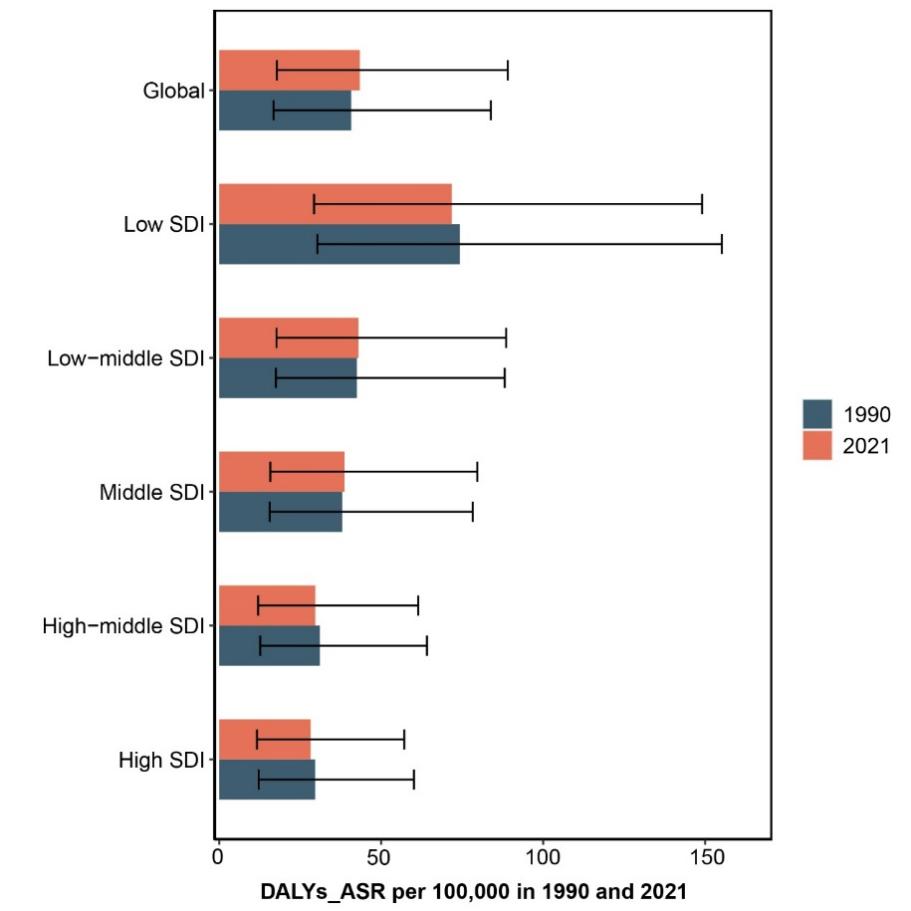


**Figure S3.** Age-standardized DALYs rates of fungal skin diseases in 1990 and 2021 for the global and 5 SDI territories. DALYs-disability-adjusted life years, SDI-social demographic index.


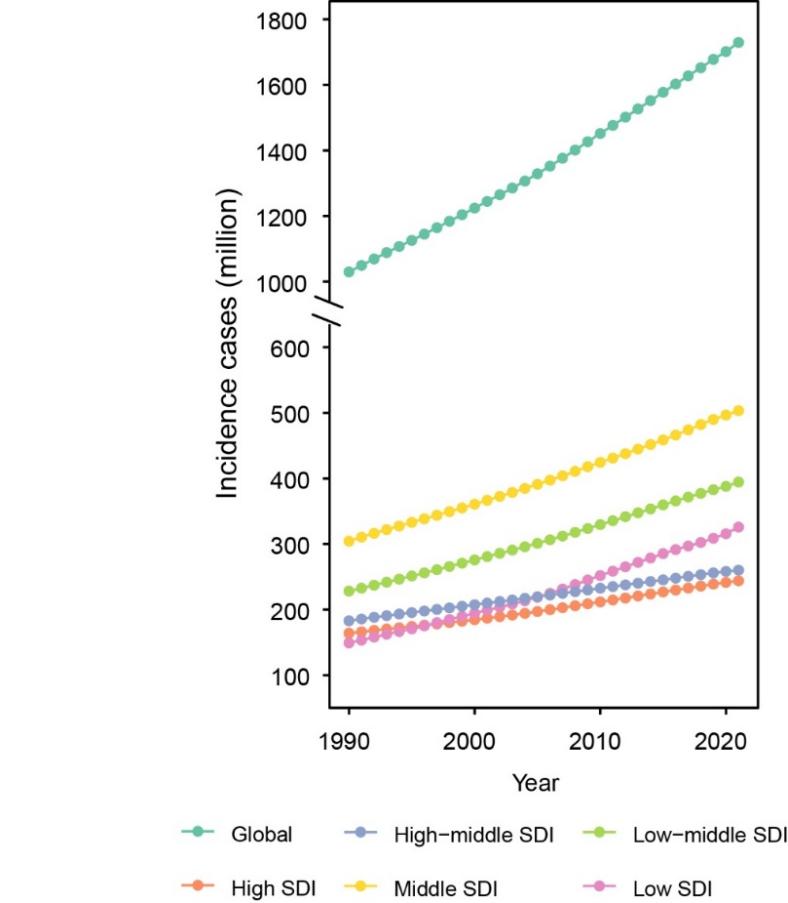


**Figure S4.** The incidents of fungal skin diseases occurred from 1990 to 2021 in the global and 5 SDI territories. SDI-social demographic index.


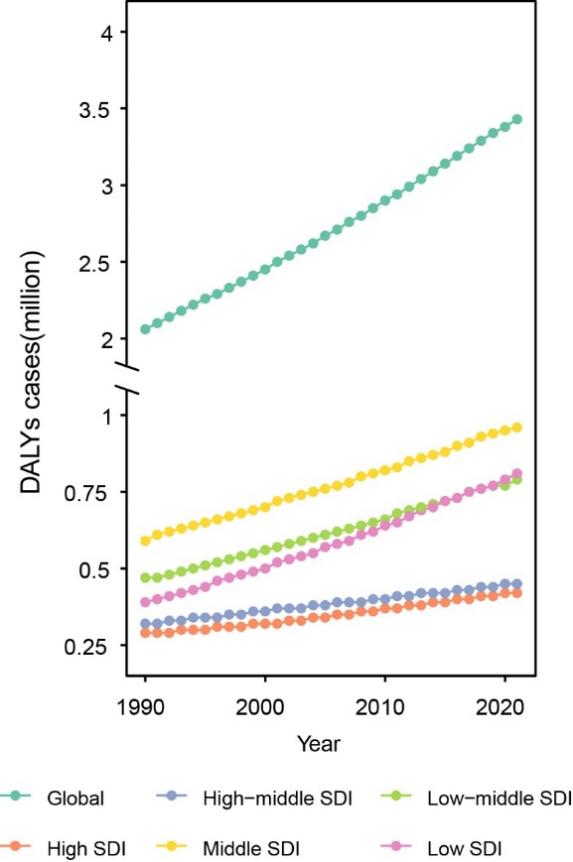


**Figure S5.** The DALYs cases of fungal skin diseases from 1990 to 2021 for the global and 5 SDI territories. DALYs-disability-adjusted life years, SDI-social demographic index.


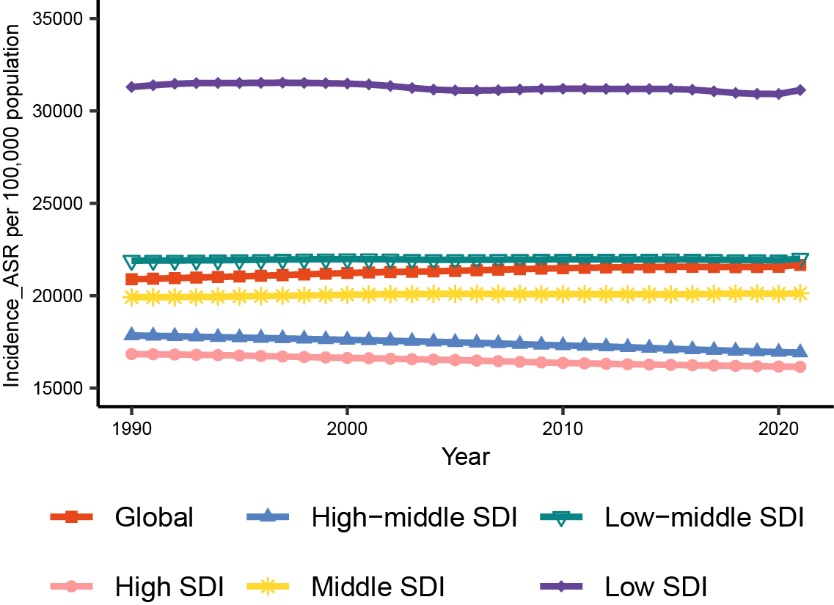


**Figure S6.** Age-standardized incidence rates of fungal skin diseases from 1990 to 2021 for the global and 5 SDI territories. SDI-social demographic index.


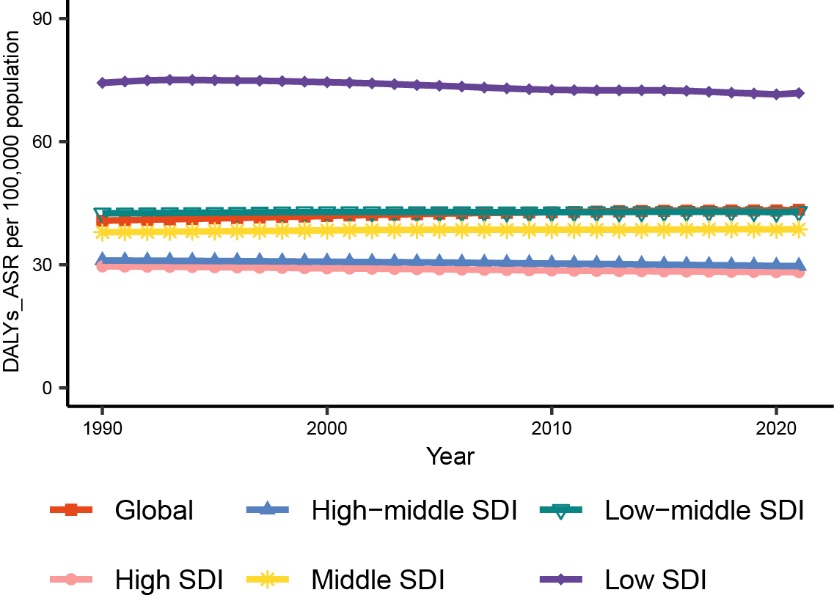


**Figure S7.** Age-standardized DALYs rates of fungal skin diseases from 1990 to 2021 for the global and 5 SDI territories. DALYs-disability-adjusted life years, SDI-social demographic index.


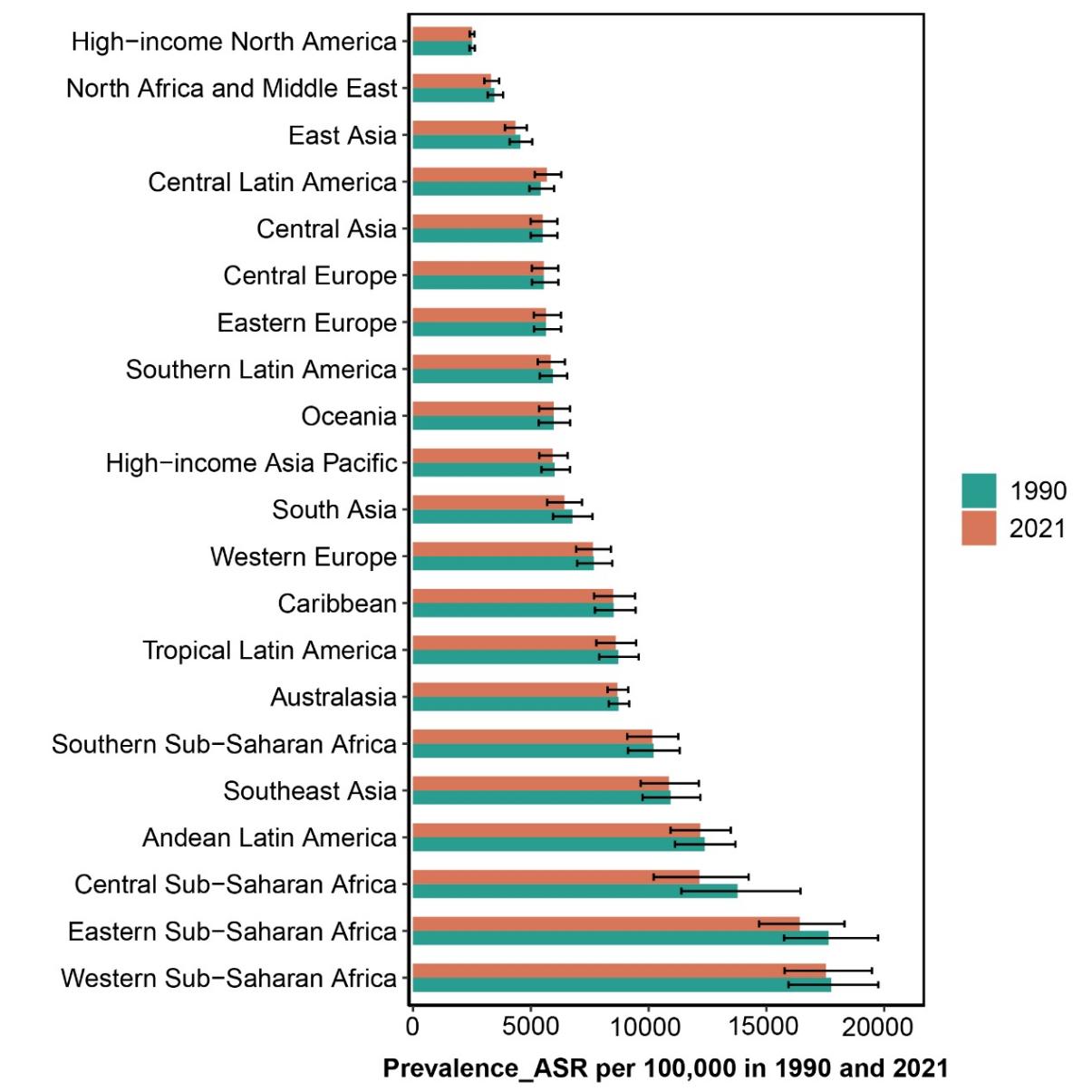


**Figure S8.** Age-standardized prevalence rates of fungal skin diseases in 1990 and 2021 across 21 territories.


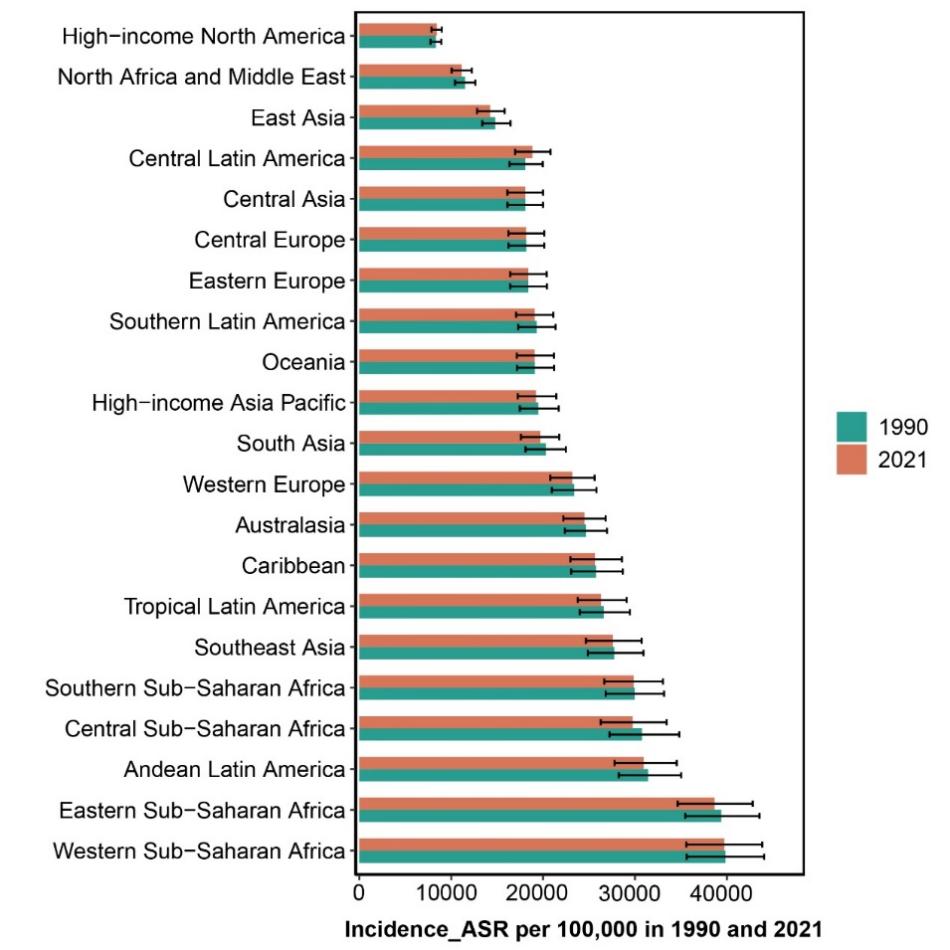


**Figure S9.** Age-standardized incidence rates of fungal skin diseases in 1990 and 2021 across 21 territories.


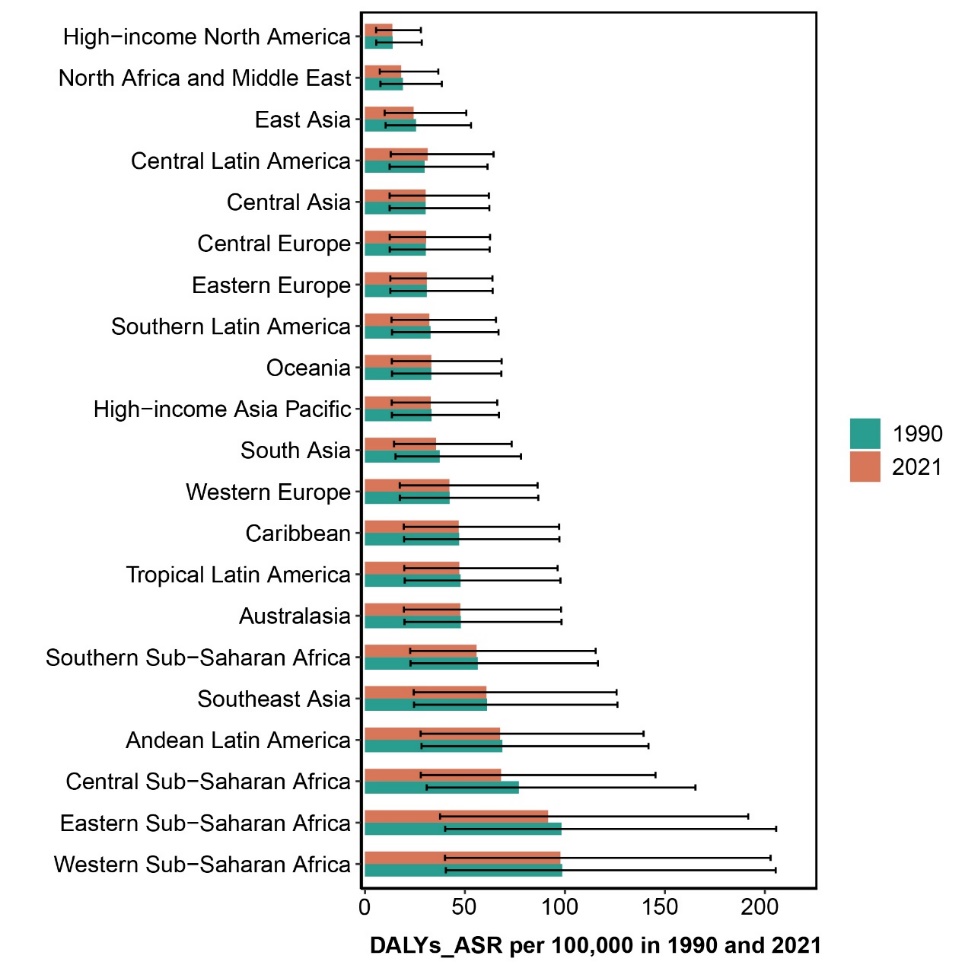


**Figure S10.** Age-standardized DALYs rates of fungal skin diseases in 1990 and 2021 across 21 territories. DALYs-disability-adjusted life years.


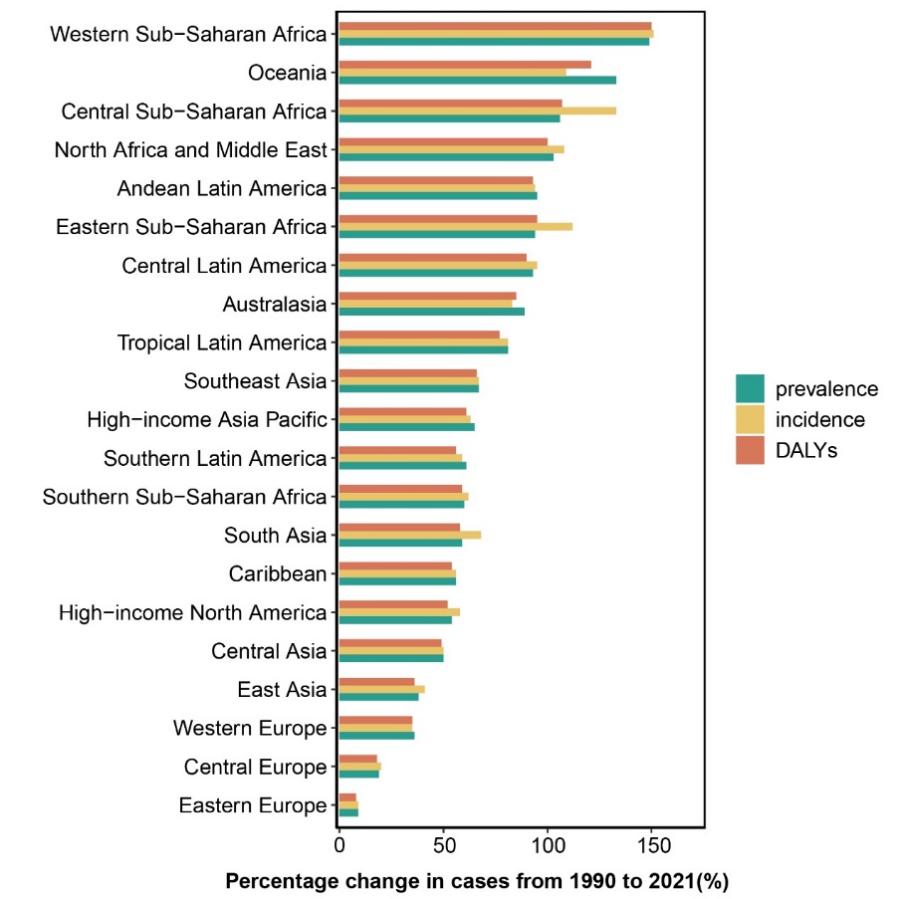


**Figure S11.** Percentage change in prevalence, incidence, and DALY cases for fungal skin diseases from 1990 to 2021 across 21 territories. DALYs-disability-adjusted life years.


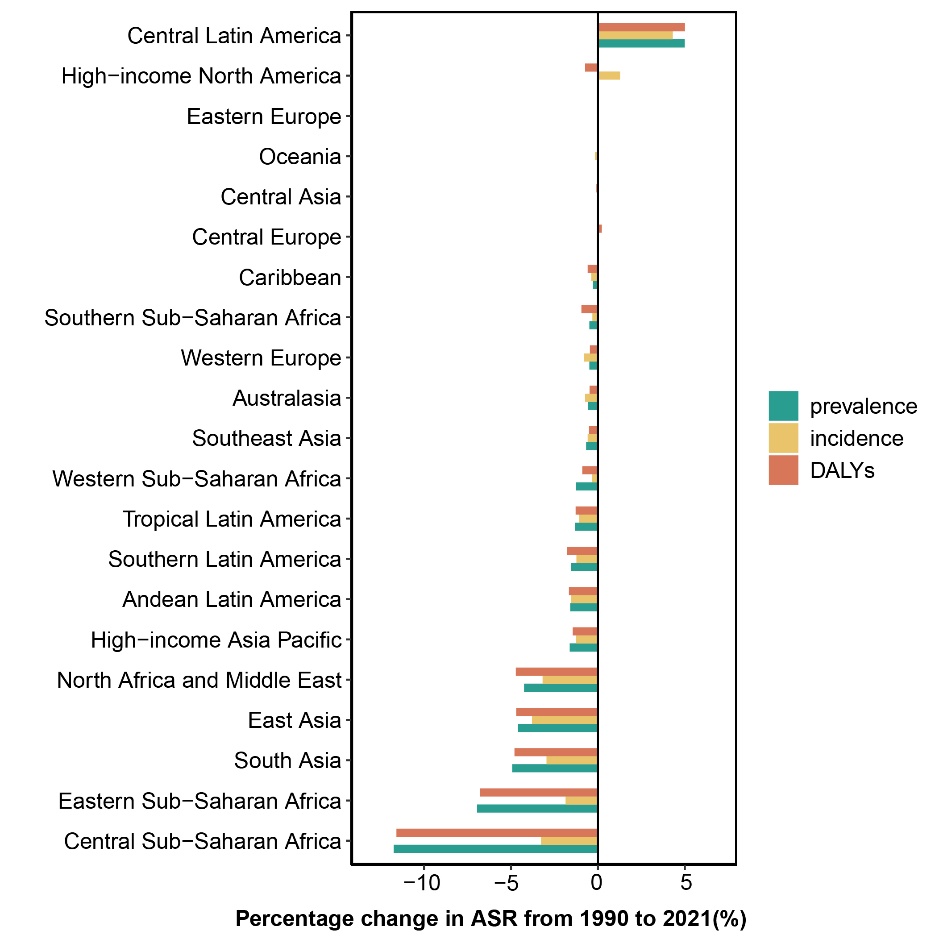


**Figure S12.** Percentage change in age-standardized rate (ASR) of prevalence, incidence, and DALYs for fungal skin diseases from 1990 to 2021 across 21 territories. DALYs-disability-adjusted life years.


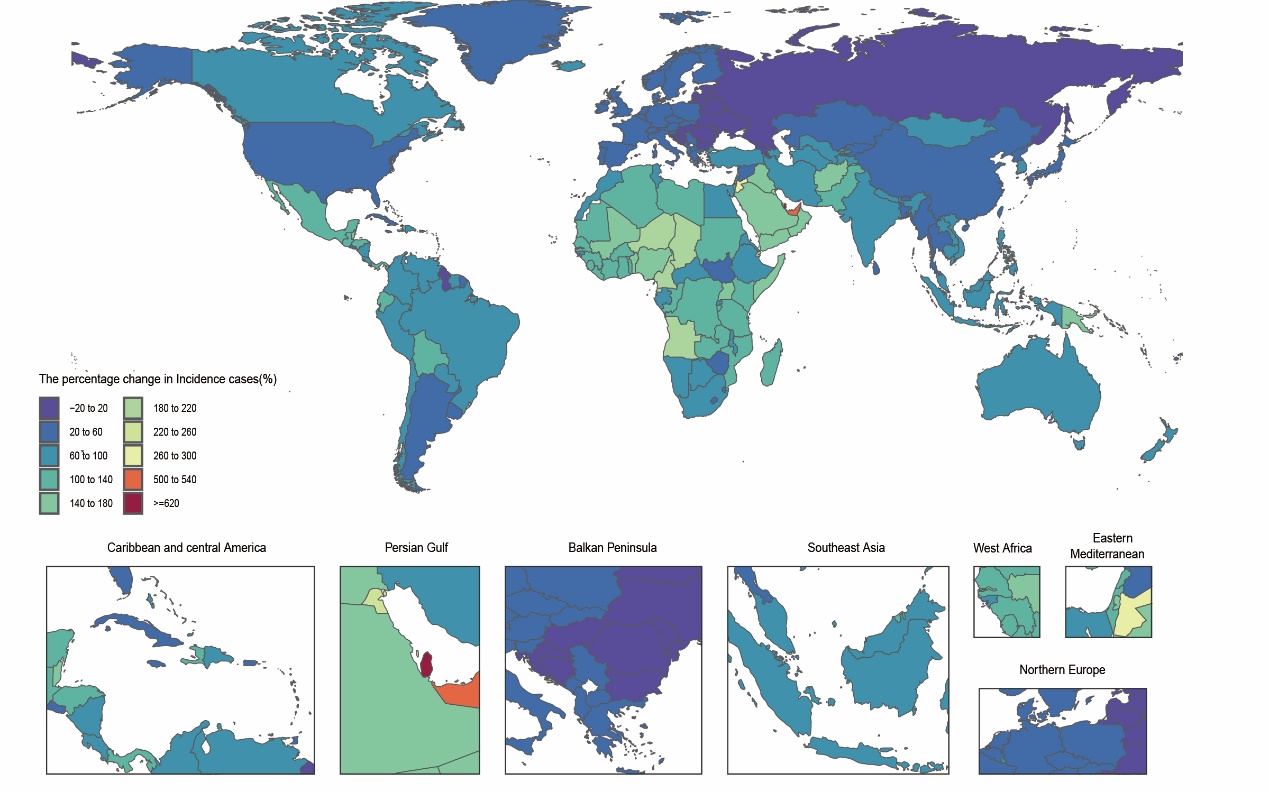


**Figure S13.** Percentage change in incidence cases for fungal skin diseases globally from 1990 to 2021.


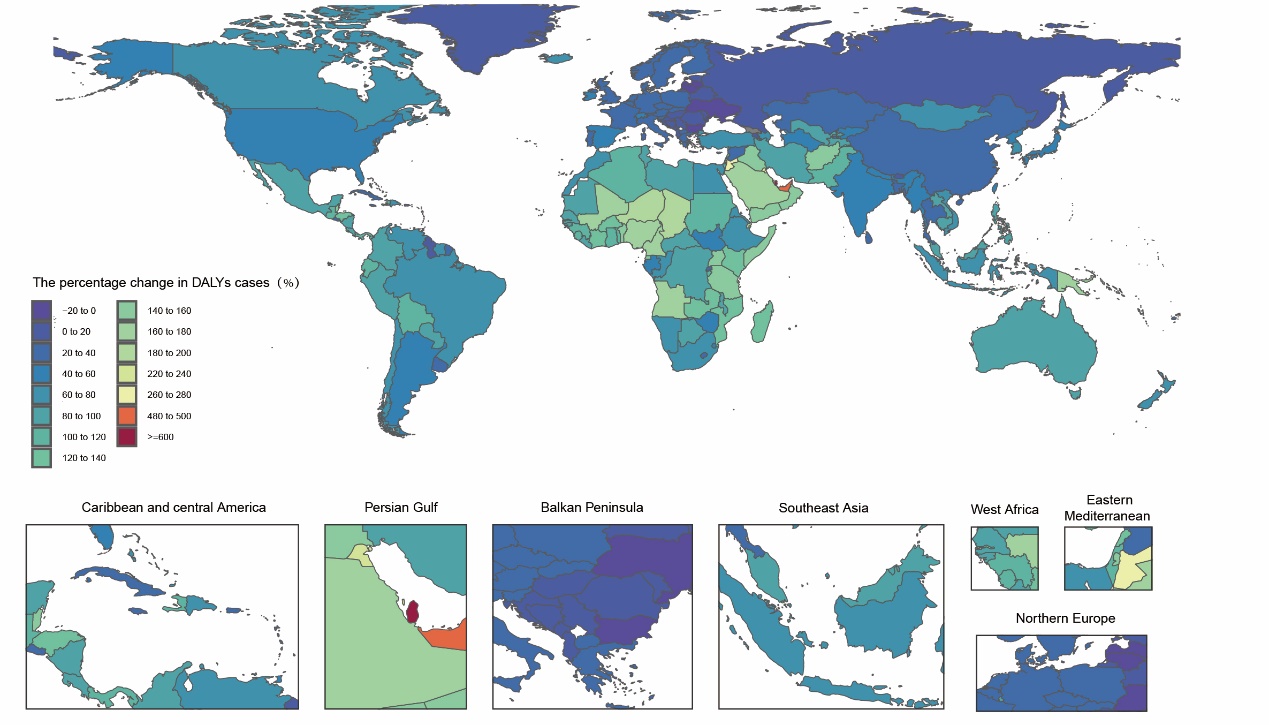


**Figure S14.** Percentage change in DALYs cases for fungal skin diseases globally from 1990 to 2021. DALYs-disability-adjusted life years.


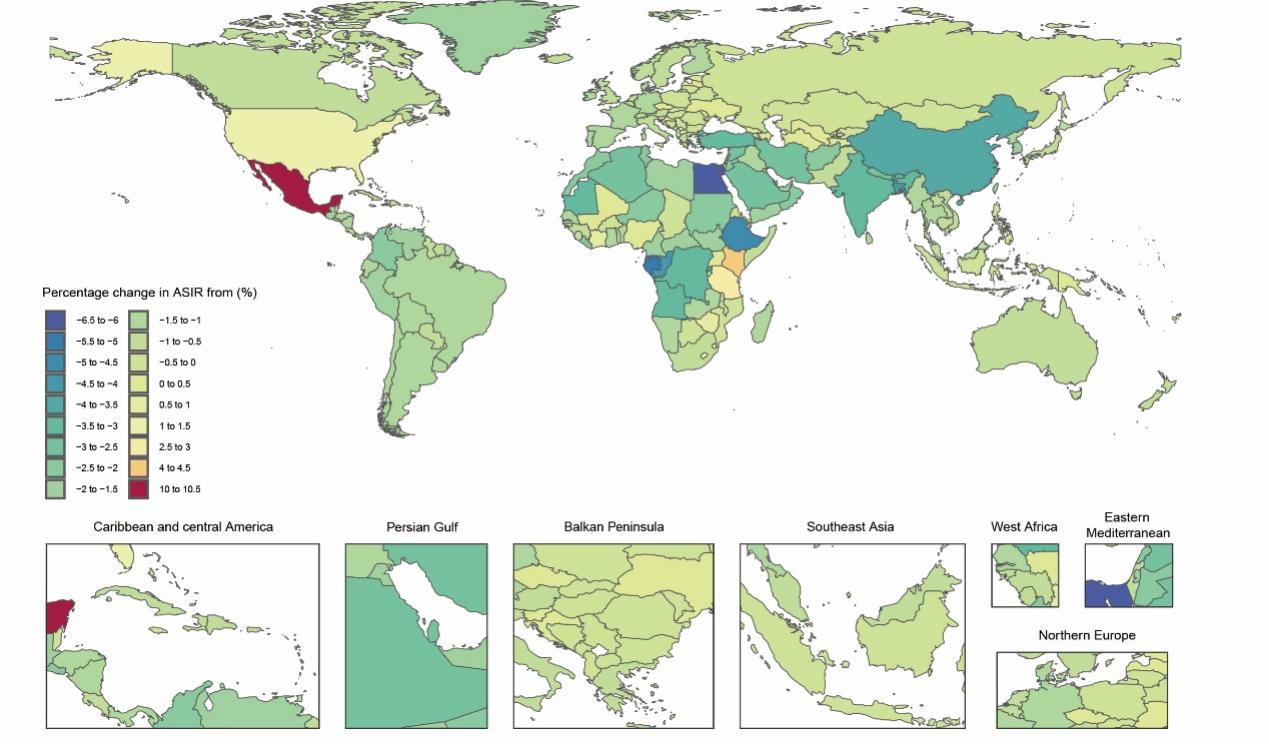


**Figure S15.** Percentage change in age-standardized incidence rate for fungal skin diseases globally from 1990 to 2021.


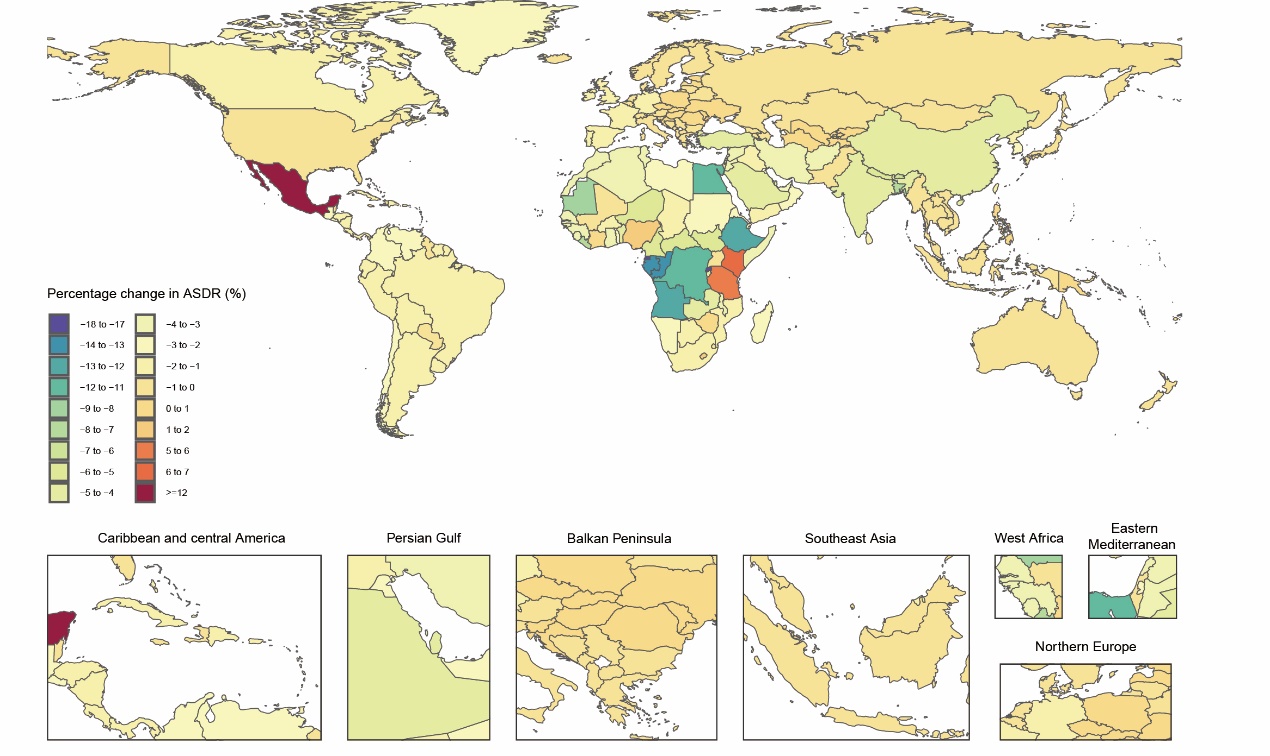


**Figure S16.** Percentage change in age-standardized DALYs rate for fungal skin diseases globally from 1990 to 2021. DALYs-disability-adjusted life years.


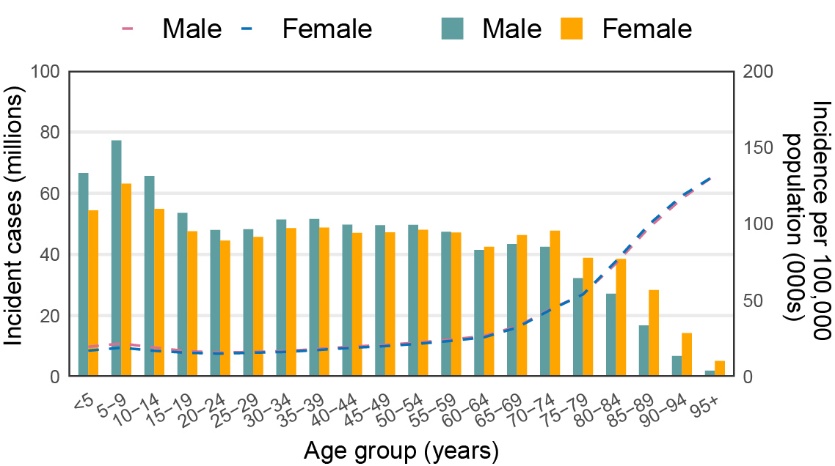


**Figure S17.** Global incident cases and age-standardized incidence rate of fungal skin diseases per 100,000 population by age and sex in 2021.


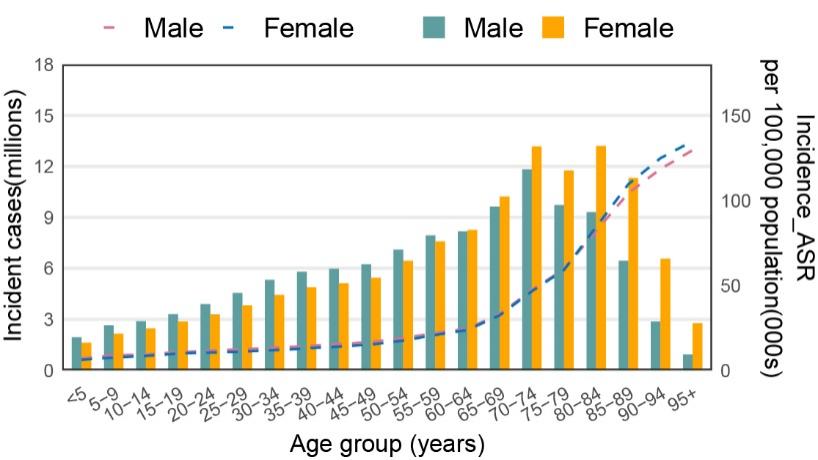


**Figure S18.** Incident cases and age-standardized incidence rate of fungal skin diseases per 100,000 population by age and sex in high SDI regions in 2021. SDI-social demographic index.


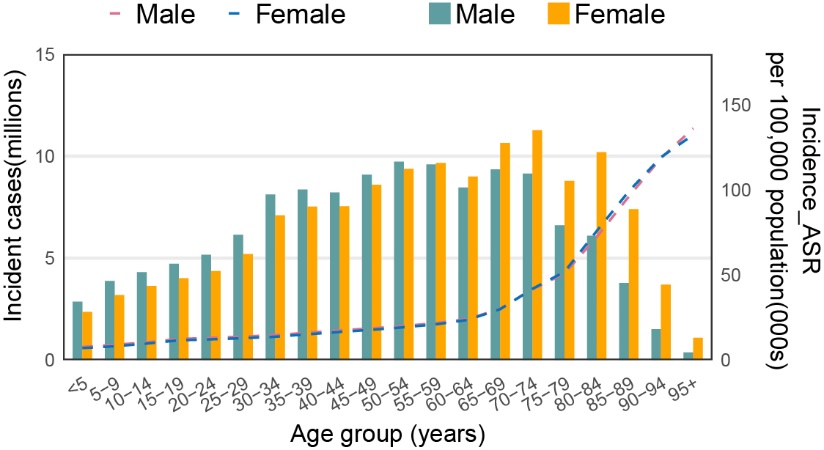


**Figure S19.** Incident cases and age-standardized incidence rate of fungal skin diseases per 100,000 population by age and sex in high-middle SDI regions in 2021. SDI-social demographic index.


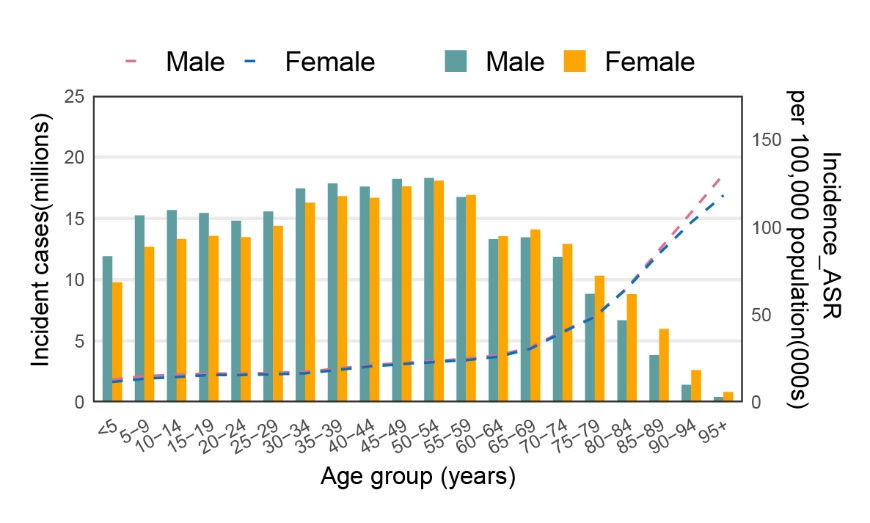


**Figure S20.** Incident cases and age-standardized incidence rate of fungal skin diseases per 100,000 population by age and sex in middle SDI regions in 2021. SDI-social demographic index.


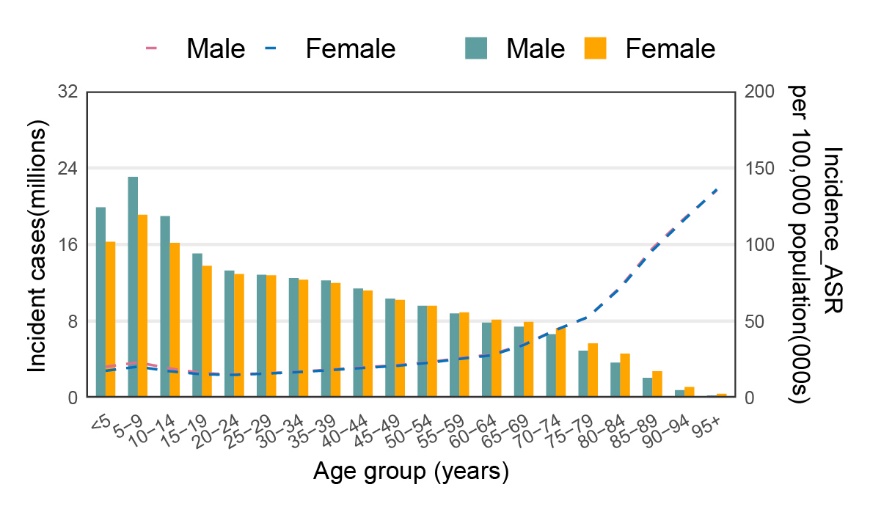


**Figure S21.** Incident cases and age-standardized incidence rate of fungal skin diseases per 100,000 population by age and sex in low-middle SDI regions in 2021. SDI-social demographic index.


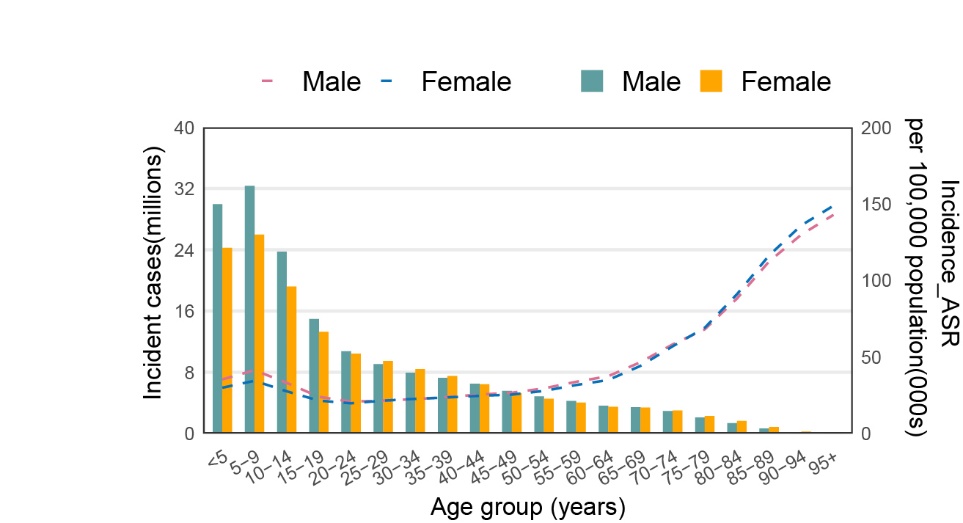


**Figure S22.** Incident cases and age-standardized incidence rate of fungal skin diseases per 100,000 population by age and sex in low SDI regions in 2021. SDI-social demographic index.


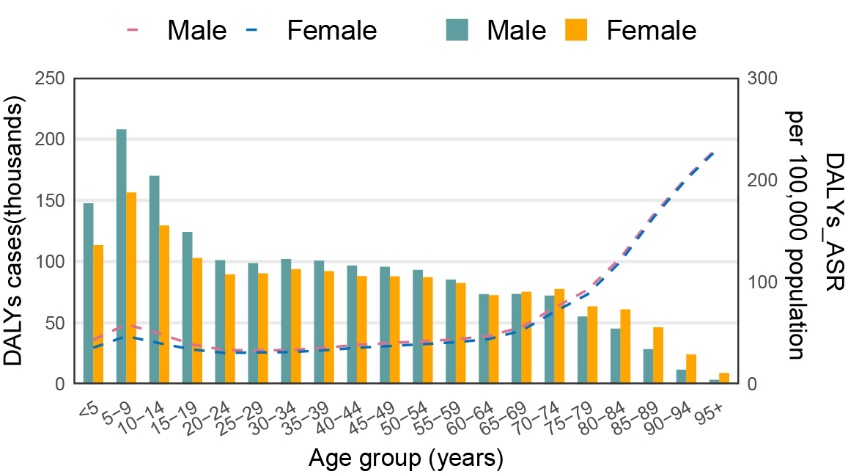


**Figure S23.** Global DALYs cases and age-standardized DALYs rate of fungal skin diseases per 100,000 population by age and sex in 2021. DALYs-disability-adjusted life years, SDI-social demographic index.


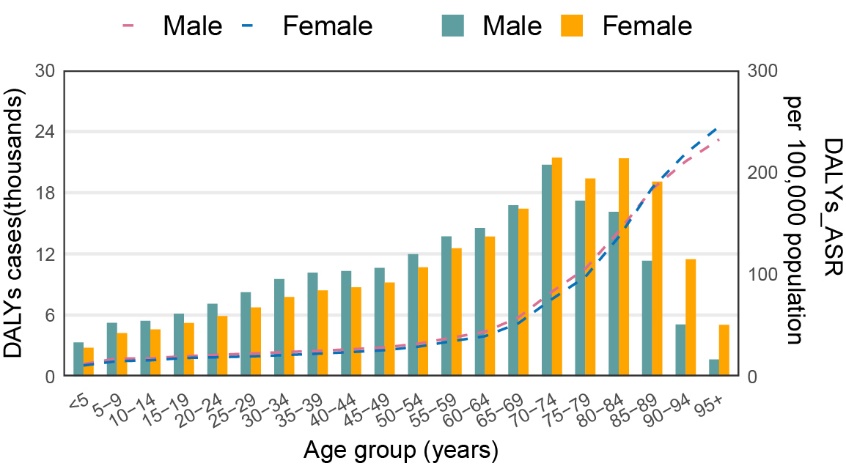


**Figure S24.** DALYs cases and age-standardized DALYs rate of fungal skin diseases per 100,000 population by age and sex in high SDI regions in 2021. DALYs-disability-adjusted life years, SDI-social demographic index.


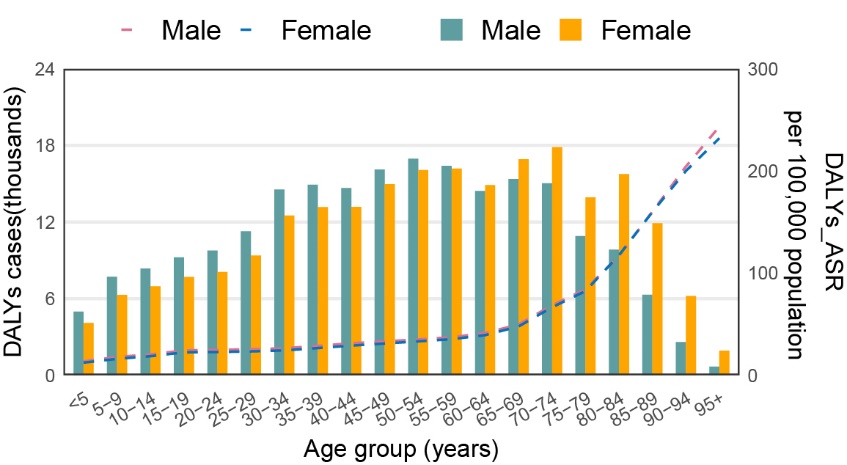


**Figure S25.** DALYs cases and age-standardized DALYs rate of fungal skin diseases per 100,000 population by age and sex in high-middle SDI regions in 2021. DALYs-disability-adjusted life years, SDI-social demographic index.


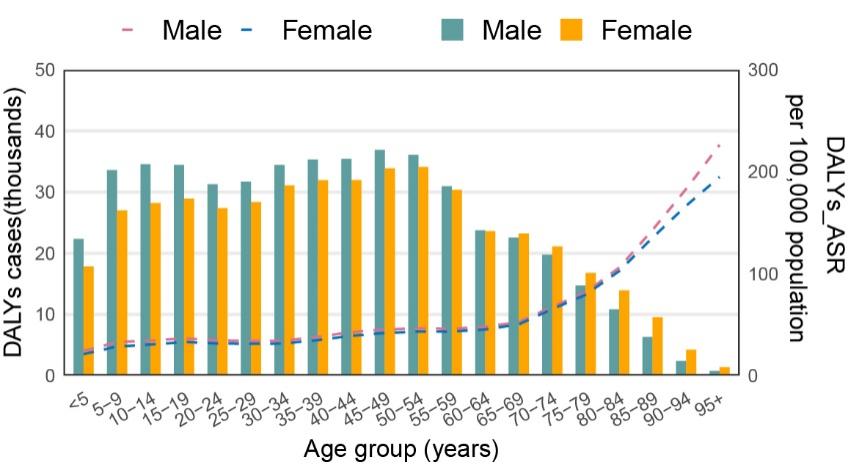


**Figure S26.** DALYs cases and age-standardized DALYs rate of fungal skin diseases per 100,000 population by age and sex in middle SDI regions in 2021. DALYs-disability-adjusted life years, SDI-social demographic index.


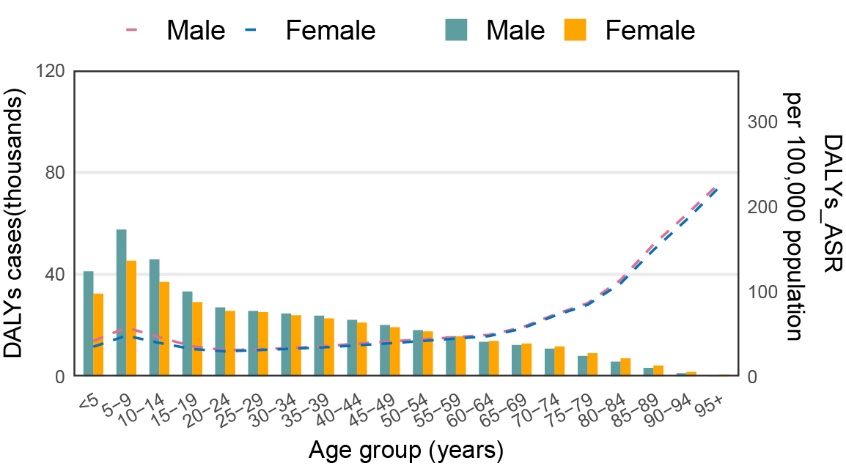


**Figure S27.** DALYs cases and age-standardized DALYs rate of fungal skin diseases per 100,000 population by age and sex in low-middle SDI regions in 2021. DALYs-disability-adjusted life years, SDI-social demographic index.


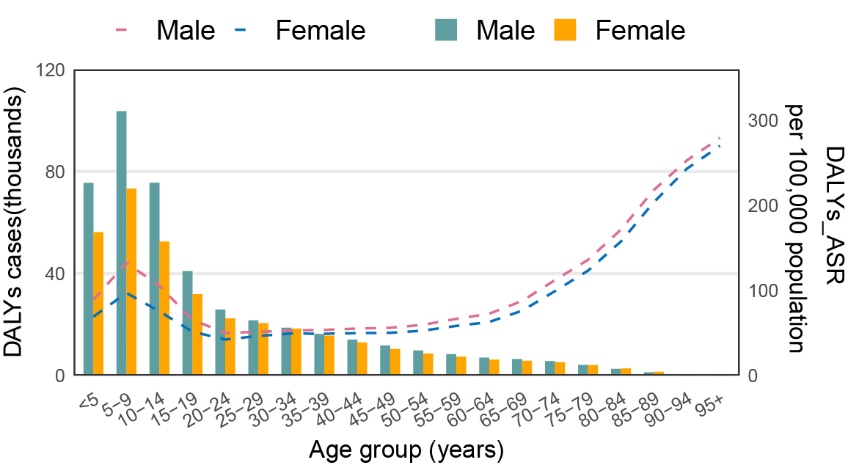


**Figure S28.** DALYs cases and age-standardized DALYs rate of fungal skin diseases per 100,000 population by age and sex in low SDI regions in 2021. DALYs-disability-adjusted life years, SDI-social demographic index.


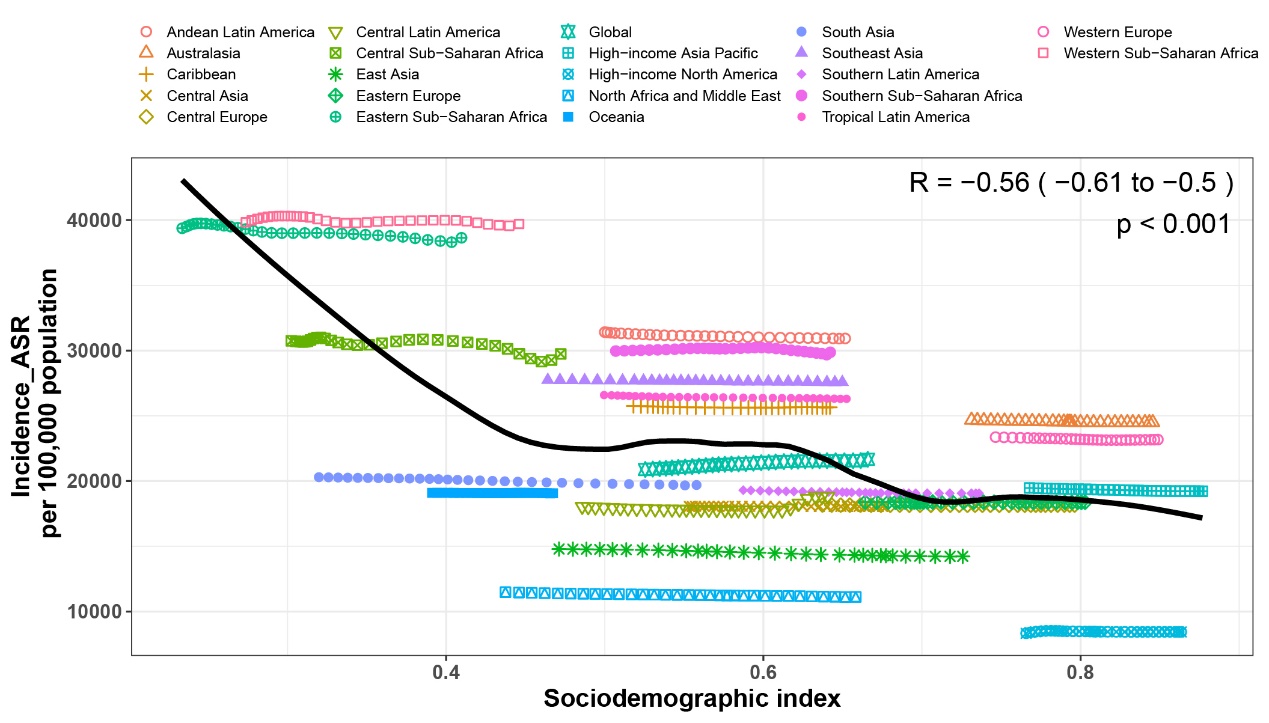


**Figure S29.** Associations between the SDI and age-standardized incidence rates per 100,000 population of fungal skin diseases across 21 GBD regions and globally. SDI-social demographic index, GBD-global burden of disease.


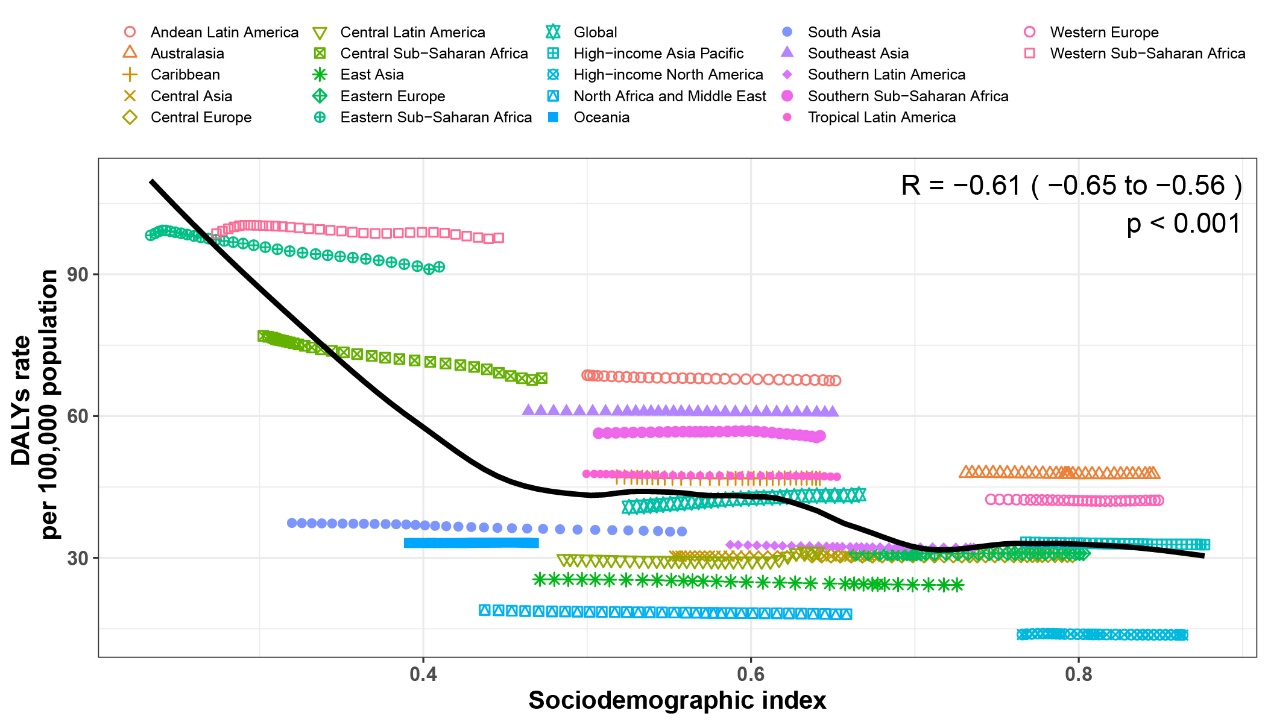


**Figure S30.** Associations between the SDI and age-standardized DALY rates per 100,000 population of fungal skin diseases across 21 GBD regions and globally. SDI-social demographic index, DALYs-disability-adjusted life years, GBD-global burden of disease.


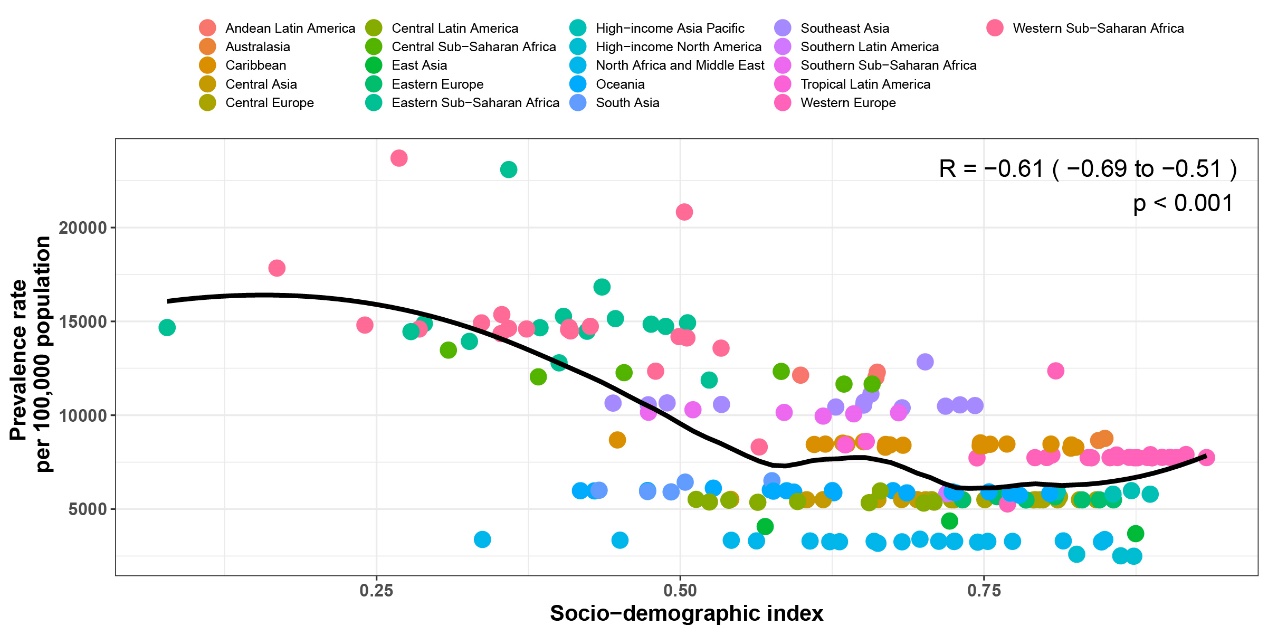


**Figure S31.** Associations between the SDI and age-standardized prevalence rates per 100,000 population of fungal skin diseases across 204 nations. SDI-social demographic index.


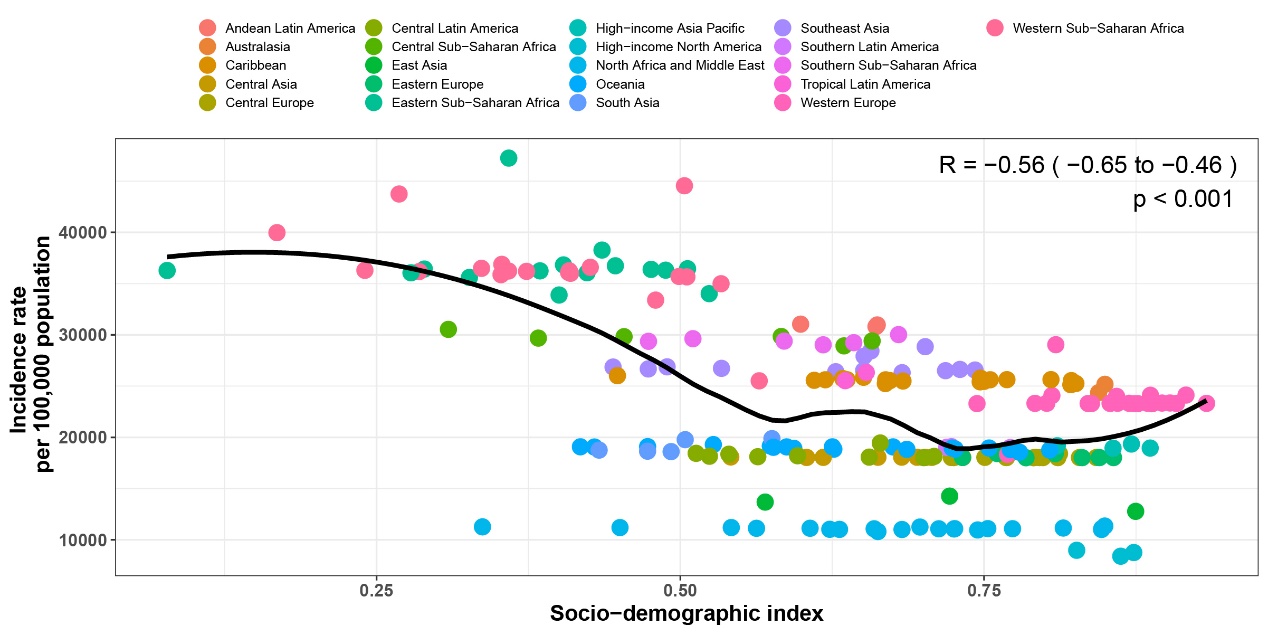


**Figure S32.** Associations between the SDI and age-standardized incidence rates per 100,000 population of fungal skin diseases across 204 nations. SDI-social demographic index.


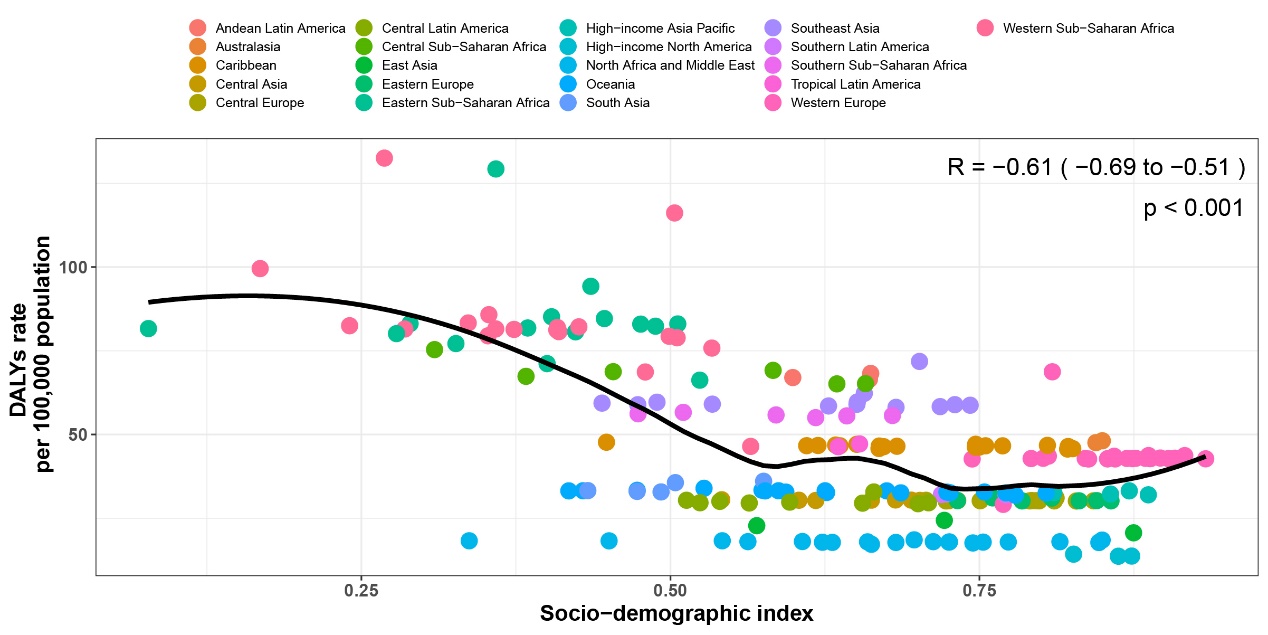


**Figure S33.** Associations between the SDI and age-standardized DALY rates per 100,000 population of fungal skin diseases across 204 nations. DALYs-disability-adjusted life years, SDI-social demographic index.

| **Table S1.** Comparative analysis of incident cases and age-standardized rate (ASR) changes in fungal skin diseases by region from 1990 to 2021 | | | | | | |
| --- | --- | --- | --- | --- | --- | --- |
| **Location** | **NO. （millions）** | | **percentage change(%)** | **ASRs per 100,000** | | **Percentage change in the ASRs per 100,000(%)** |
|  | **1990 (95%UI)** | **2021 (95%UI)** |  | **1990 (95%UI)** | **2021 (95%UI)** |  |
| Global | 1029.7 (927.1,1132.5) | 1729.2 (1562.7,1894.7) | 68 | 20886.9 (18919.7,22868.8) | 21668.4 (19601.2,23729.2) | 3.74 (2.99,4.68) |
| Low SDI | 149.3 (131.1,169.9) | 325.8 (288,368.4) | 118 | 31292.3 (28086.6,34534.5) | 31124.8 (27943.9,34383.1) | -0.54 (-1.37,0.35) |
| Low-middle SDI | 228.2 (201.6,256) | 394.5 (353.7,434.6) | 73 | 21894.6 (19644.5,24042.1) | 22004.6 (19797.4,24106.7) | 0.5 (-0.18,1.14) |
| Middle SDI | 304.3 (273.2,336.4) | 503.3 (454,554.7) | 65 | 19915.8 (18062.6,21958.4) | 20125.7 (18207,22198.1) | 1.05 (0.48,1.56) |
| High-middle SDI | 182.9 (164.6,202.7) | 260.2 (234.2,287.2) | 42 | 17846.8 (16107.9,19680.6) | 16920.1 (15266.5,18644.1) | -5.19 (-5.91,-4.5) |
| High SDI | 164 (148.7,180) | 243.9 (220.3,269.3) | 49 | 16838.1 (15269.8,18422.4) | 16147.9 (14687.3,17628.2) | -4.1 (-4.64,-3.51) |
| High-income Asia Pacific | 35.8 (32,39.9) | 58.5 (51.4,66) | 63 | 19469.5 (17465.8,21702.2) | 19223.1 (17249.7,21450.5) | -1.27 (-1.86,-0.8) |
| High-income North America | 26.2 (24.2,28) | 41.3 (38.1,44.3) | 58 | 8333.2 (7752.4,8915) | 8438.7 (7866.3,8989.7) | 1.27 (-0.21,2.9) |
| Western Europe | 106.5 (95.2,118.5) | 143.4 (127.5,160.7) | 35 | 23364.9 (20946.3,25800.2) | 23177.1 (20787.7,25596.1) | -0.8 (-1.19,-0.49) |
| Australasia | 5.3 (4.9,5.9) | 9.7 (8.8,10.7) | 83 | 24673.7 (22362.4,26952.6) | 24492 (22211.1,26796.3) | -0.74 (-1.04,-0.48) |
| Andean Latin America | 10.2 (9.1,11.4) | 19.8 (17.8,22) | 94 | 31413.7 (28239.9,35011.5) | 30930.2 (27772.9,34538) | -1.54 (-2.06,-1.13) |
| Tropical Latin America | 34.6 (31.1,38.4) | 62.7 (56.4,69.6) | 81 | 26587.8 (23989,29433.9) | 26302.5 (23768.2,29078.3) | -1.07 (-1.48,-0.77) |
| Central Latin America | 24.2 (21.7,26.8) | 47.2 (42.5,52.2) | 95 | 18047.9 (16335.8,19955.6) | 18823.5 (16934,20811.2) | 4.3 (2.1,6.19) |
| Southern Latin America | 9.2 (8.2,10.2) | 14.6 (13,16.2) | 59 | 19304.5 (17294.9,21354.4) | 19065.4 (17057,21113) | -1.24 (-1.63,-0.94) |
| Caribbean | 8.2 (7.4,9.1) | 12.8 (11.4,14.2) | 56 | 25752.8 (23051.3,28670.6) | 25655 (22966.3,28573.7) | -0.38 (-0.62,-0.2) |
| Central Europe | 24.2 (21.5,26.8) | 29 (25.7,32.2) | 20 | 18162.5 (16240,20130.2) | 18152.7 (16232.2,20130) | -0.05 (-0.19,0.08) |
| Eastern Europe | 44.8 (39.9,49.9) | 49 (43.3,54.8) | 9 | 18381.2 (16428.7,20413.9) | 18375 (16424.4,20390.2) | -0.03 (-0.23,0.13) |
| Central Asia | 10.6 (9.4,11.8) | 15.9 (14.3,17.6) | 50 | 18055.7 (16126.7,19996.8) | 18048.1 (16114.3,19997.6) | -0.04 (-0.27,0.13) |
| North Africa and Middle East | 28.8 (26.1,31.7) | 59.8 (53.9,65.7) | 108 | 11487.5 (10402,12614.1) | 11122.5 (10049.4,12248.8) | -3.18 (-4.03,-2.49) |
| South Asia | 203.3 (176.7,230.3) | 340.9 (303.8,377.4) | 68 | 20297.5 (18084.2,22480.1) | 19696.9 (17580.3,21752.5) | -2.96 (-4.18,-1.94) |
| Southeast Asia | 115.4 (102.1,130.4) | 193.1 (173,216) | 67 | 27743.2 (24856.6,30910.5) | 27580 (24668.3,30727) | -0.59 (-0.87,-0.35) |
| East Asia | 167.6 (150,188.2) | 237 (213,263.1) | 41 | 14788.1 (13376.3,16446.4) | 14227.8 (12819.4,15818.2) | -3.79 (-4.9,-2.88) |
| Oceania | 1.1 (0.9,1.2) | 2.3 (2.1,2.6) | 109 | 19096.6 (17161.9,21205.1) | 19063.3 (17141.2,21170.1) | -0.17 (-0.53,0.21) |
| Western Sub-Saharan Africa | 71.6 (63.2,80.6) | 179.4 (158.4,202.5) | 151 | 39838.8 (35618.2,44047.3) | 39705.2 (35574.6,43841) | -0.34 (-0.87,0.23) |
| Eastern Sub-Saharan Africa | 72.3 (63.5,82.8) | 153.1 (134.8,174.2) | 112 | 39374.2 (35470.3,43535.9) | 38643.8 (34630.2,42795.3) | -1.86 (-2.93,-0.65) |
| Central Sub-Saharan Africa | 16.3 (13.8,19.7) | 37.9 (32.1,44.9) | 133 | 30749.7 (27222.4,34818) | 29742.7 (26266,33437.9) | -3.27 (-5.81,0.04) |
| Southern Sub-Saharan Africa | 13.5 (12,15.1) | 21.9 (19.5,24.4) | 62 | 29964.4 (26793.6,33143) | 29868.5 (26669.8,33040.4) | -0.32 (-0.84,0.08) |

| **Table S2.** Comparative analysis of DALYs cases and age-standardized rate (ASR) changes in fungal skin diseases by region from 1990 to 2021 | | | | | | |
| --- | --- | --- | --- | --- | --- | --- |
| **Location** | **NO. （thousands）** | | **percentage change(%)** | **ASRs per 100,000** | | **Percentage change in the ASRs per 100,000** |
|  | **1990 (95%UI)** | **2021 (95%UI)** |  | **1990 (95%UI)** | **2021 (95%UI)** |  |
| Global | 2056.4 (842.5,4249) | 3429.5 (1407.9,7044.3) | 67 | 40.7 (16.8,83.8) | 43.4 (17.8,89.1) | 6.56 (5.61,7.56) |
| Low SDI | 388.5 (157.5,815.8) | 807.8 (331.7,1696.2) | 108 | 74.3 (30.3,155.2) | 71.8 (29.3,149.1) | -3.35 (-4.59,-2.04) |
| Low-middle SDI | 465.6 (190.1,970.2) | 787.3 (322.6,1623.8) | 69 | 42.5 (17.5,88.1) | 42.9 (17.7,88.6) | 1 (0.02,1.84) |
| Middle SDI | 594.7 (241.7,1243.2) | 963.1 (393.6,1990.4) | 62 | 37.9 (15.6,78.2) | 38.6 (15.8,79.7) | 1.82 (1.14,2.5) |
| High-middle SDI | 319 (129.3,667.1) | 448.1 (183.3,921.1) | 40 | 31 (12.6,64.1) | 29.7 (12,61.4) | -4.41 (-5.25,-3.55) |
| High SDI | 286.6 (118.6,583.4) | 420.2 (173.8,852.1) | 47 | 29.6 (12.2,60.1) | 28.2 (11.6,57.1) | -4.72 (-5.37,-4.09) |
| High-income Asia Pacific | 60.9 (24.6,122.9) | 98 (40.3,198) | 61 | 33.3 (13.5,67) | 32.8 (13.4,66.2) | -1.45 (-2.21,-0.76) |
| High-income North America | 43 (17.6,88.4) | 65.5 (27.2,132.6) | 52 | 13.8 (5.6,28.4) | 13.7 (5.6,28) | -0.74 (-2,0.35) |
| Western Europe | 191.3 (79.2,395) | 257.3 (106.6,533.6) | 35 | 42.4 (17.5,86.7) | 42.2 (17.4,86.4) | -0.46 (-1.01,0.05) |
| Australasia | 10.4 (4.3,21.2) | 19.2 (7.9,39) | 85 | 47.9 (19.7,98.3) | 47.7 (19.5,98.1) | -0.48 (-1.31,0.47) |
| Andean Latin America | 22.5 (9.2,46.3) | 43.4 (17.8,89.4) | 93 | 68.7 (28.3,141.8) | 67.5 (27.8,139.3) | -1.67 (-2.48,-0.91) |
| Tropical Latin America | 63.1 (26.1,128.8) | 111.7 (46.3,230.1) | 77 | 47.8 (19.9,97.7) | 47.2 (19.6,96.4) | -1.28 (-1.85,-0.8) |
| Central Latin America | 41.4 (17,85.1) | 78.6 (32.3,162.4) | 90 | 29.8 (12.3,61.2) | 31.3 (12.9,64.3) | 5 (2.73,7.32) |
| Southern Latin America | 15.6 (6.4,31.9) | 24.4 (10.1,49.8) | 56 | 32.8 (13.5,66.8) | 32.2 (13.2,65.5) | -1.78 (-2.87,-0.68) |
| Caribbean | 15.1 (6.3,31.4) | 23.3 (9.6,48.1) | 54 | 47.1 (19.6,97.3) | 46.8 (19.4,97.1) | -0.59 (-1.14,-0.05) |
| Central Europe | 40.3 (16.3,82.4) | 47.6 (19.4,96) | 18 | 30.5 (12.4,62.3) | 30.5 (12.4,62.6) | 0.22 (-0.22,0.61) |
| Eastern Europe | 74.9 (30.5,154.5) | 80.8 (32.8,164) | 8 | 31 (12.7,63.9) | 30.9 (12.7,63.8) | -0.01 (-0.42,0.38) |
| Central Asia | 18 (7.3,37.2) | 26.9 (10.8,55.5) | 49 | 30.4 (12.4,62.2) | 30.4 (12.3,62) | -0.09 (-0.71,0.52) |
| North Africa and Middle East | 49.4 (20.2,101.3) | 98.6 (40.3,201.2) | 100 | 19 (7.8,38.5) | 18.1 (7.4,36.7) | -4.72 (-5.63,-3.81) |
| South Asia | 396.3 (160.2,848.4) | 624.8 (254,1282.7) | 58 | 37.4 (15.3,78) | 35.6 (14.5,73.3) | -4.8 (-6.81,-3.17) |
| Southeast Asia | 259.7 (104.3,544.7) | 431.5 (172.6,897.3) | 66 | 61 (24.5,126.2) | 60.7 (24.4,125.8) | -0.51 (-0.9,-0.15) |
| East Asia | 292.5 (117.5,612.1) | 398.7 (162.2,830.6) | 36 | 25.5 (10.3,53) | 24.3 (9.8,50.7) | -4.7 (-5.93,-3.6) |
| Oceania | 1.9 (0.8,3.9) | 4.2 (1.7,8.6) | 121 | 33.2 (13.5,68.2) | 33.2 (13.5,68.3) | 0.03 (-1.05,1.12) |
| Western Sub-Saharan Africa | 184.6 (75.5,388.6) | 462.3 (188.7,975.3) | 150 | 98.6 (40.4,205.4) | 97.7 (40.1,202.8) | -0.9 (-1.66,-0.23) |
| Eastern Sub-Saharan Africa | 200.5 (81.6,414.7) | 390.3 (162,812) | 95 | 98.2 (40.1,205.6) | 91.6 (37.5,191.6) | -6.78 (-8.52,-4.95) |
| Central Sub-Saharan Africa | 48.8 (19.6,105.7) | 100.8 (41.1,218.4) | 107 | 77 (30.9,165.2) | 68 (27.9,145.3) | -11.59 (-15.75,-6.94) |
| Southern Sub-Saharan Africa | 26.1 (10.6,54.5) | 41.6 (16.8,86.1) | 59 | 56.4 (22.8,116.5) | 55.8 (22.6,115.3) | -0.95 (-1.69,-0.31) |

| **Table S3.**  Comparative analysis of prevalent cases and age-standardized rate (ASR) changes in fungal skin diseases across 204 nations from 1990 to 2021 | | | | | | |
| --- | --- | --- | --- | --- | --- | --- |
| **Location** | **NO.** | | **percentage change(%)** | **ASRs per 100,000** | | **Percentage change in the ASRs per 100,000(%)** |
|  | **1990 (95%UI)** | **2021 (95%UI)** |  | **1990 (95%UI)** | **2021 (95%UI)** |  |
| China | 50395632 (44888051,56527047) | 69601875 (62751479,77349821) | 38 | 4579.5 (4122.4,5088) | 4363.6 (3920.7,4851.3) | -4.7 (-6,-3.7) |
| North Korea | 802351 (720376,894822) | 1150253 (1038835,1277204) | 43 | 4126.2 (3728.6,4581.4) | 4069 (3677.4,4509.9) | -1.4 (-2.4,-0.5) |
| Taiwan (province of China) | 727575 (653842,811763) | 1057684 (961568,1177445) | 45 | 3763.7 (3409,4155.4) | 3691.5 (3334.6,4072.5) | -1.9 (-2.8,-1.2) |
| Cambodia | 927146 (812753,1047648) | 1716842 (1515147,1944545) | 85 | 10661.2 (9506.5,12006) | 10554.9 (9409.1,11879.2) | -1 (-1.7,-0.4) |
| Indonesia | 18771931 (16513231,21188640) | 31456817 (27591739,35597049) | 68 | 11172.4 (9960.6,12510.9) | 11127.1 (9908.6,12470.5) | -0.4 (-0.7,-0.2) |
| Laos | 386686 (341628,437104) | 739664 (651566,838370) | 91 | 10773.5 (9605.4,12120.5) | 10655.2 (9516.5,12023.7) | -1.1 (-1.8,-0.5) |
| Malaysia | 1688535 (1484651,1911257) | 3362209 (2970690,3815705) | 99 | 10613.9 (9463.7,11954.4) | 10515.5 (9375.4,11873.8) | -0.9 (-1.5,-0.5) |
| Maldives | 19487 (17013,22046) | 55762 (48738,64402) | 186 | 10635.9 (9487.8,12007.3) | 10541.3 (9392.2,11932) | -0.9 (-1.9,0.1) |
| Myanmar | 3912657 (3441208,4431325) | 5902709 (5228813,6693432) | 51 | 10708 (9556.1,12073) | 10573.6 (9439,11917.8) | -1.3 (-1.8,-0.7) |
| Philippines | 5927486 (5195973,6760959) | 11640996 (10252970,13255974) | 96 | 10717.9 (9532.7,12116.2) | 10699.9 (9509.6,12098) | -0.2 (-0.3,0) |
| Sri Lanka | 2159426 (1955112,2348104) | 2972158 (2735634,3200909) | 38 | 12995.1 (11887.1,14019) | 12842.2 (11727.1,13868.7) | -1.2 (-1.6,-0.8) |
| Thailand | 5673412 (4987267,6447186) | 8015282 (7093269,9053766) | 41 | 10516.2 (9368.4,11857.1) | 10394.2 (9243.2,11738.2) | -1.2 (-1.6,-0.8) |
| Timor-Leste | 70962 (62217,80390) | 134610 (118297,152394) | 90 | 10714.4 (9569.8,12069.2) | 10646.6 (9501.6,12002.2) | -0.6 (-1.1,-0.2) |
| Viet Nam | 6361647 (5593988,7194766) | 10692313 (9426060,12167692) | 68 | 10523.9 (9383.6,11858.3) | 10438.3 (9293.8,11786.2) | -0.8 (-1.4,-0.3) |
| Fiji | 39980 (35114,45100) | 52917 (46933,59306) | 32 | 5984.3 (5345.2,6687) | 5979.5 (5346.3,6684.3) | -0.1 (-0.9,0.7) |
| Kiribati | 4021 (3537,4533) | 6728 (5941,7579) | 67 | 6172.7 (5526,6862.6) | 6112.9 (5461.5,6816.3) | -1 (-2.1,0) |
| Marshall Islands | 2296 (2023,2603) | 3120 (2757,3524) | 36 | 6051.9 (5415.4,6761.9) | 6022.1 (5383.4,6743.2) | -0.5 (-1.3,0.3) |
| Federated States of Micronesia | 5438 (4791,6122) | 5748 (5100,6479) | 6 | 6083.3 (5443.4,6804.9) | 5976.7 (5337.7,6680.4) | -1.8 (-2.7,-0.9) |
| Papua New Guinea | 208465 (184490,235279) | 548237 (483739,618810) | 163 | 5967.5 (5326.4,6655.6) | 5977.3 (5356.3,6667.3) | 0.2 (-0.6,1) |
| Samoa | 8737 (7732,9852) | 11456 (10180,12834) | 31 | 5979.5 (5346.3,6679.3) | 5907.7 (5269.3,6597.9) | -1.2 (-2,-0.5) |
| Solomon Islands | 17061 (15044,19261) | 35988 (31704,40605) | 111 | 6022.7 (5391.4,6723.7) | 5970.8 (5346.9,6661.7) | -0.9 (-1.7,-0.1) |
| Tonga | 5089 (4515,5718) | 5800 (5163,6459) | 14 | 5908.5 (5267.2,6597.6) | 5873.5 (5245.2,6548.6) | -0.6 (-1.3,0) |
| Vanuatu | 7667 (6777,8648) | 16695 (14755,18776) | 118 | 6000.9 (5372.5,6707.7) | 5990.5 (5362.6,6682.9) | -0.2 (-0.9,0.5) |
| Armenia | 168897 (151924,189057) | 194628 (176349,217897) | 15 | 5502.7 (4990.4,6132.4) | 5497.3 (4983.2,6128.3) | -0.1 (-0.2,0) |
| Azerbaijan | 343852 (309756,385341) | 560978 (503736,626735) | 63 | 5504.8 (4996.5,6135) | 5505.2 (4993.7,6133.3) | 0 (-0.3,0.3) |
| Georgia | 314169 (284921,350473) | 253432 (230433,283605) | -19 | 5497.1 (4987.6,6127) | 5498.4 (4983.8,6126.9) | 0 (-0.1,0.2) |
| Kazakhstan | 800639 (723624,894806) | 986963 (889430,1107823) | 23 | 5498.8 (4994.5,6127.2) | 5491.7 (4980.7,6121.2) | -0.1 (-0.3,0) |
| Kyrgyzstan | 206693 (186673,231393) | 323686 (291583,362673) | 57 | 5503.5 (4997.5,6129.7) | 5497.6 (4983.5,6126.7) | -0.1 (-0.3,0.1) |
| Mongolia | 91405 (81819,103320) | 155946 (140405,174702) | 71 | 5525.6 (5015.7,6152.4) | 5501.8 (4989.9,6128.3) | -0.4 (-0.6,-0.3) |
| Tajikistan | 228357 (204842,257533) | 450238 (403466,506094) | 97 | 5515.6 (5007.2,6138.7) | 5518.8 (5017.3,6144.6) | 0.1 (-0.3,0.4) |
| Turkmenistan | 157952 (141327,178107) | 253122 (228368,283110) | 60 | 5510.2 (5002.9,6136.2) | 5508.8 (4998.7,6130.2) | 0 (-0.3,0.2) |
| Uzbekistan | 919825 (827867,1035727) | 1661582 (1495467,1857508) | 81 | 5507.1 (4999.9,6130.6) | 5507 (4997.6,6135.3) | 0 (-0.2,0.2) |
| Albania | 151982 (136776,170633) | 182953 (165169,205796) | 20 | 5511.1 (5006,6135.4) | 5501.6 (4996.6,6129.6) | -0.2 (-0.4,0.1) |
| Bosnia and Herzegovina | 233741 (210084,261321) | 244858 (221218,274995) | 5 | 5499.9 (4990.5,6123.7) | 5495.3 (4985.3,6120.5) | -0.1 (-0.2,0) |
| Bulgaria | 539165 (486713,606674) | 538370 (485520,608374) | 0 | 5505.3 (4998.7,6131.8) | 5495.2 (4982.6,6119.7) | -0.2 (-0.4,0) |
| Croatia | 288702 (261242,321773) | 337426 (305079,378805) | 17 | 5490.3 (4981.3,6114.9) | 5491.9 (4980.1,6118.4) | 0 (-0.2,0.2) |
| Czechia | 639504 (581438,711940) | 826178 (746969,930752) | 29 | 5494 (4981.5,6120.9) | 5494.4 (4984.8,6120.1) | 0 (-0.2,0.2) |
| Hungary | 658660 (597805,737262) | 751867 (680377,843599) | 14 | 5495.3 (4981.2,6123.3) | 5488.8 (4975,6115.5) | -0.1 (-0.2,0) |
| North Macedonia | 105364 (95108,117892) | 141246 (127257,158710) | 34 | 5510.8 (5009.4,6136.8) | 5501.8 (4995.8,6126.9) | -0.2 (-0.3,0) |
| Montenegro | 34131 (30872,38142) | 41109 (37146,46250) | 20 | 5497.3 (4986.1,6122.7) | 5494.2 (4982.2,6122) | -0.1 (-0.1,0) |
| Poland | 2265705 (2062491,2527892) | 2934423 (2665122,3309186) | 30 | 5662.1 (5148.6,6294.9) | 5653.1 (5139,6285.8) | -0.2 (-0.3,0) |
| Romania | 1350148 (1224104,1509620) | 1456287 (1318969,1632391) | 8 | 5505.2 (4996.6,6130.6) | 5493.7 (4982,6118.4) | -0.2 (-0.3,-0.1) |
| Serbia | 547835 (493816,610878) | 659632 (597132,741983) | 20 | 5503 (4992.4,6129.2) | 5498.5 (4990.1,6123) | -0.1 (-0.2,0) |
| Slovakia | 303810 (276109,339313) | 387191 (350446,433702) | 27 | 5497.2 (4983.9,6124.3) | 5491.5 (4977.6,6117.8) | -0.1 (-0.2,0) |
| Slovenia | 119463 (108563,133224) | 168351 (152466,188819) | 41 | 5491.3 (4984.3,6114) | 5495.7 (4987.1,6120.9) | 0.1 (-0.2,0.4) |
| Belarus | 622483 (565924,694625) | 659596 (598292,740295) | 6 | 5491.5 (4986.6,6118) | 5486 (4975.2,6114.8) | -0.1 (-0.2,0) |
| Estonia | 95973 (87570,106853) | 104603 (94871,117520) | 9 | 5490.7 (4983.9,6120.5) | 5491.1 (4980,6116.6) | 0 (-0.2,0.2) |
| Latvia | 165394 (150940,184184) | 151562 (137493,170409) | -8 | 5491.8 (4985.6,6120.1) | 5490 (4979.4,6117.5) | 0 (-0.2,0.1) |
| Lithuania | 220317 (200815,245541) | 220830 (200276,248085) | 0 | 5495.4 (4986,6123.1) | 5491.5 (4980,6118.8) | -0.1 (-0.2,0) |
| Moldova | 235679 (212719,263538) | 251457 (228211,281442) | 7 | 5500.2 (4988.5,6129.9) | 5495 (4983.2,6120.4) | -0.1 (-0.2,0) |
| Russia | 9015265 (8197553,10071712) | 10306776 (9381267,11588538) | 14 | 5658.1 (5146,6292.5) | 5655.1 (5141.8,6288) | -0.1 (-0.3,0.2) |
| Ukraine | 3339406 (3042327,3741098) | 3230991 (2932119,3642320) | -3 | 5658.4 (5144.6,6293.3) | 5658.7 (5144.7,6292.8) | 0 (-0.2,0.2) |
| Brunei | 12070 (10712,13610) | 24054 (21574,26894) | 99 | 6029.7 (5460.5,6642.8) | 5918 (5369.9,6518.7) | -1.9 (-2.6,-1.3) |
| Japan | 8569807 (7745078,9539194) | 13757388 (12363740,15526047) | 61 | 6040 (5453.6,6692.1) | 5981.3 (5397.7,6631.8) | -1 (-1.3,-0.7) |
| South Korea | 2266780 (2028373,2543205) | 3956153 (3557922,4398608) | 75 | 5939.8 (5379.8,6550.6) | 5794.7 (5242.8,6402.4) | -2.4 (-3.4,-1.7) |
| Singapore | 160643 (143516,179546) | 399519 (360182,442924) | 149 | 5884 (5332,6479.5) | 5795 (5241.7,6397.9) | -1.5 (-2.1,-1) |
| Australia | 1573279 (1503873,1645281) | 2975614 (2855101,3088616) | 89 | 8704.6 (8306.2,9144.5) | 8654.9 (8262.1,9096.4) | -0.6 (-0.8,-0.4) |
| New Zealand | 321366 (289885,355981) | 575559 (522492,637437) | 79 | 8828.8 (8023,9751.1) | 8764.2 (7946.3,9695.2) | -0.7 (-1.1,-0.5) |
| Andorra | 4384 (3923,4884) | 8694 (7904,9731) | 98 | 7841.1 (7069.2,8689.2) | 7754.5 (6986.1,8591.2) | -1.1 (-1.5,-0.8) |
| Austria | 723338 (654927,801412) | 964289 (876314,1075611) | 33 | 7807 (7051.4,8650.5) | 7740.2 (6972.4,8581.7) | -0.9 (-1.4,-0.4) |
| Belgium | 931343 (844290,1034702) | 1235016 (1126796,1376649) | 33 | 7809.5 (7054.5,8654.5) | 7743.1 (6972.3,8585.3) | -0.8 (-1.3,-0.5) |
| Cyprus | 61187 (55002,67779) | 125707 (113794,140103) | 105 | 7849.2 (7086.8,8690.7) | 7741.2 (6973.4,8579.4) | -1.4 (-2,-1) |
| Denmark | 490345 (444230,543225) | 623325 (565961,697009) | 27 | 7844.1 (7085,8690.1) | 7756.7 (6989.7,8599) | -1.1 (-1.6,-0.7) |
| Finland | 452374 (409915,501167) | 632587 (572210,707678) | 40 | 7826.8 (7067.1,8671.9) | 7744.3 (6978.2,8586.4) | -1.1 (-1.7,-0.6) |
| France | 5266321 (4779499,5839686) | 7297650 (6645461,8137054) | 39 | 7822.1 (7062,8661.8) | 7731.5 (6963.2,8574.2) | -1.2 (-1.7,-0.8) |
| Germany | 7598595 (6886109,8428405) | 9772584 (8852733,10909113) | 29 | 7839.4 (7077.1,8685.3) | 7756 (6987.3,8606) | -1.1 (-1.7,-0.5) |
| Greece | 939412 (850491,1048360) | 1198928 (1087688,1336738) | 28 | 7781.3 (7013.2,8625.3) | 7739.3 (6972.6,8582.9) | -0.5 (-0.8,-0.3) |
| Iceland | 20891 (18843,23132) | 33821 (30746,37712) | 62 | 7793 (7025.5,8638) | 7735.6 (6967.9,8583.5) | -0.7 (-1.1,-0.4) |
| Ireland | 294387 (265853,326990) | 466833 (424304,520666) | 59 | 7821 (7055.3,8661.2) | 7732 (6963.9,8574.6) | -1.1 (-1.7,-0.8) |
| Israel | 606086 (570384,649941) | 1288332 (1214671,1376649) | 113 | 12454.1 (11753.6,13279) | 12362.6 (11679.2,13199) | -0.7 (-1,-0.5) |
| Italy | 5421464 (4888851,6064852) | 7236858 (6514598,8116550) | 33 | 7956.6 (7174.8,8837.5) | 7880.5 (7096.8,8746.6) | -1 (-1.3,-0.7) |
| Luxembourg | 34515 (31239,38268) | 62401 (56772,69635) | 81 | 7838.5 (7086.5,8680.8) | 7750.4 (6988.6,8587.3) | -1.1 (-1.7,-0.6) |
| Malta | 30380 (27457,33589) | 49337 (44727,55162) | 62 | 7813.6 (7044.4,8658.9) | 7746.7 (6976.9,8588.8) | -0.9 (-1.3,-0.5) |
| Netherlands | 1317409 (1193937,1459167) | 1834115 (1666890,2054140) | 39 | 7799.9 (7038,8647) | 7737.5 (6969.7,8578.3) | -0.8 (-1.2,-0.4) |
| Norway | 414497 (374327,462145) | 562208 (507493,627040) | 36 | 7988 (7199.9,8869.2) | 7904.4 (7114.1,8770.1) | -1 (-1.5,-0.7) |
| Portugal | 891482 (804942,994855) | 1228917 (1114468,1373063) | 38 | 7834.3 (7073.1,8673.7) | 7725.9 (6955.4,8567.7) | -1.4 (-2,-0.9) |
| Spain | 2408325 (2188293,2674335) | 3634573 (3285234,4076606) | 51 | 5359.2 (4890.2,5875.5) | 5276 (4818.2,5795.5) | -1.6 (-2,-1.2) |
| Sweden | 870782 (786931,974509) | 1142833 (1034072,1279870) | 31 | 7962.6 (7175.5,8834.5) | 7897.4 (7110,8760.9) | -0.8 (-1.3,-0.5) |
| Switzerland | 643640 (584659,711497) | 961299 (873126,1071486) | 49 | 7808.6 (7037.6,8654.4) | 7742.5 (6973.8,8584.2) | -0.8 (-1.3,-0.5) |
| UK | 5492616 (4977249,6131930) | 7139899 (6467656,7988990) | 30 | 7953.9 (7182.9,8830.4) | 7881.2 (7102.4,8757.1) | -0.9 (-1.3,-0.6) |
| Argentina | 1915768 (1735249,2118156) | 2943700 (2667023,3250461) | 54 | 5944.3 (5392.6,6553.9) | 5857.2 (5305.6,6458.9) | -1.5 (-2,-1) |
| Chile | 705379 (634492,786863) | 1262592 (1144727,1397616) | 79 | 5923.2 (5370.8,6526.8) | 5815.9 (5276.3,6419.6) | -1.8 (-2.5,-1.3) |
| Uruguay | 201404 (182378,222616) | 253690 (229734,280960) | 26 | 5903.8 (5351.3,6502.8) | 5825.1 (5278.4,6424.9) | -1.3 (-1.8,-0.9) |
| Canada | 741289 (667273,827821) | 1290901 (1163522,1457562) | 74 | 2517.3 (2268.4,2807.4) | 2490 (2245.3,2781.6) | -1.1 (-1.6,-0.6) |
| USA | 7133690 (6835528,7422895) | 10947152 (10532222,11390673) | 53 | 2505.8 (2399.7,2611.5) | 2509 (2416.8,2590.1) | 0.1 (-1.1,1.2) |
| Antigua and Barbuda | 4946 (4464,5509) | 7939 (7167,8845) | 61 | 8426.3 (7634.3,9368.4) | 8385.5 (7599.6,9306.8) | -0.5 (-0.9,-0.1) |
| The Bahamas | 18833 (16697,21221) | 33077 (29943,36921) | 76 | 8526.9 (7725.9,9462.8) | 8462.8 (7667.4,9391.9) | -0.8 (-1.3,-0.3) |
| Barbados | 22904 (20648,25558) | 31430 (28241,35301) | 37 | 8438.6 (7648.3,9355.7) | 8365.1 (7578.8,9302.4) | -0.9 (-1.3,-0.5) |
| Belize | 12936 (11544,14540) | 32227 (28787,36128) | 149 | 8467.8 (7682.8,9407.4) | 8442.3 (7655.7,9366.5) | -0.3 (-0.7,0.1) |
| Cuba | 894792 (806911,1002036) | 1191568 (1071425,1332121) | 33 | 8375.9 (7599.9,9311.7) | 8294.9 (7504.8,9232.7) | -1 (-1.4,-0.6) |
| Dominica | 5760 (5202,6439) | 6166 (5578,6881) | 7 | 8505.9 (7691.5,9439.4) | 8527.1 (7719.3,9469.8) | 0.2 (-0.3,0.8) |
| Dominican Republic | 501525 (444331,567754) | 901044 (811481,1003292) | 80 | 8533 (7732.9,9462.5) | 8462.1 (7668.8,9397.7) | -0.8 (-1.4,-0.4) |
| Grenada | 7009 (6348,7816) | 8848 (7987,9851) | 26 | 8487 (7682.9,9425.3) | 8443.1 (7645.4,9395.6) | -0.5 (-1,-0.1) |
| Guyana | 54800 (48560,62004) | 60767 (54762,67830) | 11 | 8637.1 (7822.2,9569.6) | 8586.8 (7776.9,9523.3) | -0.6 (-1.2,0) |
| Haiti | 454275 (405317,512794) | 932996 (832209,1051115) | 105 | 8762.3 (7924.9,9713.2) | 8683.1 (7867.4,9637.8) | -0.9 (-1.7,-0.3) |
| Jamaica | 182700 (164497,203791) | 248401 (225062,277165) | 36 | 8416.2 (7634.6,9336.2) | 8401.1 (7607.9,9330.3) | -0.2 (-0.6,0.2) |
| Saint Lucia | 10056 (8991,11274) | 16821 (15217,18754) | 67 | 8481.9 (7685.7,9418.9) | 8417.4 (7636.3,9349.9) | -0.8 (-1.3,-0.4) |
| Saint Vincent and the Grenadines | 8120 (7242,9080) | 10447 (9462,11662) | 29 | 8468.2 (7667.3,9397.3) | 8461.9 (7676.1,9391.6) | -0.1 (-0.6,0.4) |
| Suriname | 29027 (25924,32473) | 50474 (45720,56042) | 74 | 8577.9 (7779.6,9507.1) | 8514.1 (7721.2,9460.9) | -0.7 (-1.3,-0.2) |
| Trinidad and Tobago | 91735 (82202,102495) | 133469 (120819,149147) | 45 | 8547.3 (7738.2,9474.6) | 8463.9 (7663.4,9386.1) | -1 (-1.5,-0.5) |
| Bolivia | 651145 (576733,731482) | 1320272 (1173417,1473602) | 103 | 12292 (11061.3,13611.6) | 12134.1 (10883.4,13423.8) | -1.3 (-1.9,-0.8) |
| Ecuador | 1029620 (906904,1158110) | 2111780 (1877954,2357799) | 105 | 12183.2 (10943,13472) | 12021.6 (10775.8,13319.5) | -1.3 (-1.9,-0.9) |
| Peru | 2338992 (2056889,2639067) | 4371839 (3907548,4857577) | 87 | 12498.1 (11228.5,13850.2) | 12292.3 (11010.1,13653.1) | -1.6 (-2.3,-1.1) |
| Colombia | 1490082 (1341128,1666971) | 2755096 (2494697,3056513) | 85 | 5496.5 (4972,6093.8) | 5341.7 (4835.5,5928.3) | -2.8 (-3.8,-2) |
| Costa Rica | 138737 (125040,155199) | 268081 (242638,296913) | 93 | 5380.7 (4872.4,5965.4) | 5320.6 (4813,5902.8) | -1.1 (-1.7,-0.7) |
| El Salvador | 249583 (224343,278486) | 346198 (312872,384072) | 39 | 5526.3 (5001.2,6138.8) | 5362.3 (4844.7,5952.2) | -3 (-4,-2.1) |
| Guatemala | 375624 (336348,425325) | 770256 (692387,858783) | 105 | 5613.6 (5084.7,6244.1) | 5471.3 (4945.1,6066.7) | -2.5 (-3.6,-1.6) |
| Honduras | 212220 (189210,238460) | 480999 (431673,536795) | 127 | 5582.4 (5065,6189.1) | 5516.8 (5000.3,6113.5) | -1.2 (-2,-0.4) |
| Mexico | 3784375 (3410213,4212310) | 7588938 (6890913,8430945) | 101 | 5316.8 (4841.6,5853.1) | 5965.3 (5425.7,6614.9) | 12.2 (7.6,16.8) |
| Nicaragua | 167414 (149530,188939) | 322895 (292068,359232) | 93 | 5463.4 (4942.6,6057.6) | 5379.7 (4871.5,5968.7) | -1.5 (-2.3,-0.9) |
| Panama | 113234 (101936,126128) | 234330 (211634,259789) | 107 | 5466.5 (4938.2,6051.4) | 5371.2 (4860.1,5962.8) | -1.7 (-2.6,-1.1) |
| Venezuela | 860327 (772443,962895) | 1485673 (1344994,1645704) | 73 | 5513.9 (4992.4,6116.7) | 5402.8 (4892.3,5992.8) | -2 (-2.9,-1.3) |
| Brazil | 11061557 (9924560,12400502) | 19847606 (17999176,21931557) | 79 | 8716.8 (7908.8,9591.5) | 8603 (7784.5,9473.4) | -1.3 (-1.7,-1) |
| Paraguay | 284507 (254467,318980) | 562775 (504761,627767) | 98 | 8471.6 (7672.4,9394.3) | 8420.6 (7633.2,9356.1) | -0.6 (-1.1,-0.3) |
| Algeria | 630980 (568872,701387) | 1301131 (1182241,1444426) | 106 | 3396.3 (3099.9,3755.8) | 3282.8 (2990.5,3625.1) | -3.3 (-4.5,-2.5) |
| Bahrain | 12607 (11113,14351) | 42916 (38330,48333) | 240 | 3389.8 (3074.9,3737.1) | 3279.7 (2980.2,3622.9) | -3.2 (-4.4,-2.3) |
| Egypt | 1599067 (1469360,1757634) | 2717076 (2465998,2990054) | 70 | 3688.8 (3396.7,4068.8) | 3300.8 (3008.3,3653.2) | -10.5 (-12.9,-8.3) |
| Iran | 1444592 (1291359,1607138) | 2806933 (2533131,3120342) | 94 | 3505.7 (3181.7,3869.4) | 3395.8 (3078.2,3749.5) | -3.1 (-4,-2.3) |
| Iraq | 415685 (375908,464613) | 1022828 (922143,1146947) | 146 | 3224.4 (2935.1,3558.8) | 3177.8 (2885.5,3510.5) | -1.4 (-1.8,-1.2) |
| Jordan | 86105 (77402,96356) | 325778 (294401,364261) | 278 | 3378.3 (3075.8,3735.1) | 3273.5 (2980.7,3613.4) | -3.1 (-4.1,-2.2) |
| Kuwait | 41822 (37068,47518) | 135534 (120942,152309) | 224 | 3326.3 (3022,3675.3) | 3251.7 (2959.5,3598.1) | -2.2 (-3.2,-1.5) |
| Lebanon | 83877 (76103,92710) | 193830 (176076,214365) | 131 | 3336 (3040.1,3686.6) | 3238 (2953.1,3577.4) | -2.9 (-3.9,-2.1) |
| Libya | 102876 (92825,113870) | 204336 (184369,227835) | 99 | 3362.3 (3058.5,3712.1) | 3290 (2995,3629.3) | -2.1 (-2.9,-1.5) |
| Morocco | 679575 (613999,752290) | 1163066 (1059711,1288960) | 71 | 3411.5 (3114.9,3771.1) | 3306.4 (3012.1,3650.1) | -3.1 (-4.1,-2.2) |
| Palestine | 47866 (43191,53218) | 124973 (112552,139619) | 161 | 3330.8 (3036.2,3678.4) | 3261.7 (2967.8,3600.6) | -2.1 (-2.8,-1.4) |
| Oman | 47221 (42251,53060) | 122538 (108325,139064) | 159 | 3371.2 (3061.8,3728.8) | 3279.8 (2977.6,3618.4) | -2.7 (-3.7,-1.9) |
| Qatar | 11065 (9668,12799) | 79403 (68790,92527) | 618 | 3397 (3088.6,3749.9) | 3279.5 (2979.9,3622.5) | -3.5 (-4.6,-2.6) |
| Saudi Arabia | 389046 (349821,434424) | 1025007 (910430,1158918) | 163 | 3436.7 (3124,3792.4) | 3306 (3011.8,3653.3) | -3.8 (-5.1,-2.8) |
| Syria | 300798 (270716,334877) | 423380 (384331,469972) | 41 | 3372 (3072.8,3727.1) | 3265.5 (2975.1,3608.4) | -3.2 (-4.2,-2.3) |
| Tunisia | 218895 (197484,243106) | 403696 (366039,446553) | 84 | 3340.7 (3039.9,3689.6) | 3256.2 (2965.4,3600.2) | -2.5 (-3.3,-1.8) |
| Türkiye | 1583196 (1441812,1751180) | 2884519 (2622215,3193384) | 82 | 3428.9 (3142.7,3784.6) | 3286.2 (2998.4,3630.1) | -4.2 (-5.2,-3.2) |
| United Arab Emirates | 47014 (41369,54053) | 282833 (246589,327310) | 502 | 3464 (3147.5,3825.4) | 3377.4 (3077.8,3745.2) | -2.5 (-4,-1.1) |
| Yemen | 314659 (282571,351725) | 806683 (725446,901511) | 156 | 3396.2 (3085.8,3755.9) | 3342.7 (3041.8,3695.2) | -1.6 (-2.5,-0.9) |
| Afghanistan | 278764 (252021,311291) | 707745 (633184,797092) | 154 | 3483.9 (3176.6,3850) | 3380.9 (3083,3730.9) | -3 (-4.2,-1.9) |
| Bangladesh | 6576614 (5532734,7846717) | 9255261 (8265702,10368087) | 41 | 6425.8 (5651.3,7255.1) | 5914.9 (5297.7,6578.4) | -8 (-12,-4.7) |
| Bhutan | 36000 (30541,42145) | 42180 (37724,47103) | 17 | 6324.9 (5579.6,7079.9) | 5934.1 (5295.2,6609.6) | -6.2 (-9,-3.7) |
| India | 56022068 (47705458,65483728) | 87230762 (77093668,98204047) | 56 | 6853.2 (6021.7,7753.5) | 6510.3 (5751.9,7304.6) | -5 (-7.1,-3.3) |
| Nepal | 1149738 (977112,1347327) | 1738172 (1537434,1966679) | 51 | 6355.1 (5609.3,7189.2) | 6003.8 (5342.6,6726) | -5.5 (-8.6,-2.8) |
| Pakistan | 6795585 (5818424,7949149) | 13830281 (11894115,15936194) | 104 | 6489.8 (5751.1,7284.9) | 6435.2 (5676.2,7215.8) | -0.8 (-2.2,0.4) |
| Angola | 1626785 (1250098,2053539) | 4436263 (3372026,5600060) | 173 | 13978.2 (11490.4,16787.5) | 12266.8 (10208.3,14529.1) | -12.2 (-17.6,-6.2) |
| Central African Republic | 443528 (339654,564116) | 796228 (622090,998480) | 80 | 14354.9 (11797.9,17324.3) | 13467 (11189.3,16029.6) | -6.2 (-11.6,-1.6) |
| Congo (Brazzaville) | 387895 (300249,488799) | 667141 (524301,823398) | 72 | 14245.8 (11687.4,17050.6) | 12334.5 (10289.5,14561.3) | -13.4 (-18.9,-7.3) |
| DR Congo | 5930733 (4531591,7580797) | 11446990 (8951292,14136384) | 93 | 13655.3 (11279.6,16367) | 12043.4 (10095.6,14170.6) | -11.8 (-17,-5.9) |
| Equatorial Guinea | 69040 (52889,86819) | 177400 (138205,221247) | 157 | 14124.6 (11497.5,16815) | 11660.4 (9805.9,13840.1) | -17.4 (-23,-11.4) |
| Gabon | 146127 (139359,153343) | 210480 (199322,221981) | 44 | 13468.5 (12784.8,14208) | 11659.5 (10993.2,12357.7) | -13.4 (-14.9,-12.1) |
| Burundi | 889914 (735257,1076870) | 1908562 (1594759,2254425) | 114 | 15603.2 (13695.3,17879.7) | 14882.3 (13131,16870.3) | -4.6 (-8.2,-1.6) |
| Comoros | 71454 (59373,86064) | 105018 (90393,121933) | 47 | 15360 (13501.1,17475.1) | 14849.4 (13045.8,17025.5) | -3.3 (-6.5,-0.5) |
| Djibouti | 59829 (49879,71684) | 170568 (146915,200022) | 185 | 15132.5 (13258.2,17333.5) | 14730 (12981.5,16835) | -2.7 (-6.4,0.9) |
| Eritrea | 540570 (441347,651574) | 963165 (811253,1131733) | 78 | 15695.6 (13691.3,17913.2) | 15264.7 (13422.9,17486.4) | -2.7 (-6.2,1.3) |
| Ethiopia | 15434199 (13040302,18125099) | 25775618 (21624531,29836686) | 67 | 26537 (23195.9,29871.6) | 23086.2 (20202.8,25968.6) | -13 (-16.1,-10) |
| Kenya | 2516503 (2078313,3016065) | 5636115 (4771664,6596370) | 124 | 11224.2 (9874.3,12867) | 11871.2 (10482.5,13507.2) | 5.8 (2.5,9.1) |
| Madagascar | 1433002 (1268041,1639759) | 3236943 (2878511,3650204) | 126 | 13145.4 (11869.4,14566.3) | 12790.5 (11524.7,14126.6) | -2.7 (-4.5,-1.4) |
| Malawi | 1493695 (1232918,1785309) | 2734821 (2287540,3244635) | 83 | 15280.9 (13364.5,17487.6) | 14661.6 (12901.1,16718) | -4.1 (-7.5,-0.8) |
| Mauritius | 108940 (96008,123877) | 147624 (130557,167204) | 36 | 10523.2 (9385,11872.6) | 10480.9 (9337.9,11827.1) | -0.4 (-0.7,-0.2) |
| Mozambique | 1826157 (1656343,2022638) | 4115301 (3687682,4567197) | 125 | 14119.3 (12898.9,15502.7) | 13934.1 (12709.1,15255.6) | -1.3 (-3,0.3) |
| Rwanda | 1691286 (1481938,1862094) | 2217571 (2032542,2410457) | 31 | 20393 (18446.8,22276.9) | 16830.9 (15513.8,18257.4) | -17.5 (-20.4,-13.4) |
| Seychelles | 7285 (6438,8230) | 11709 (10348,13308) | 61 | 10603.2 (9465.5,11955.6) | 10550.3 (9409.3,11915.6) | -0.5 (-1,-0.1) |
| Somalia | 1205535 (994054,1450817) | 3088455 (2556356,3701272) | 156 | 15209.3 (13361.7,17436.9) | 14670.3 (12954.4,16656.1) | -3.5 (-7,-0.2) |
| Tanzania | 3629669 (3279700,4077072) | 8667917 (7890776,9404155) | 139 | 14452.3 (13308.5,15837.8) | 15145.1 (13965.5,16398.4) | 4.8 (0.3,8.7) |
| Uganda | 2498169 (2072984,2991432) | 6076623 (5047124,7290723) | 143 | 14667.1 (13016.2,16781.3) | 14474.4 (12798.5,16554.3) | -1.3 (-4.6,2) |
| Zambia | 1257181 (1027254,1516586) | 2806575 (2319982,3318542) | 123 | 15682.7 (13633.6,18000.8) | 14923.1 (13093.2,16999.5) | -4.8 (-8.7,-1.8) |
| Botswana | 113626 (98756,130821) | 215277 (190185,243677) | 89 | 10153.2 (9141.5,11264.4) | 10073.8 (9081.1,11242.5) | -0.8 (-2,0.4) |
| Lesotho | 136205 (119829,155467) | 171182 (149322,195213) | 26 | 10072.6 (9059.9,11189.9) | 10291.8 (9260.8,11527.3) | 2.2 (0.6,3.9) |
| Namibia | 122766 (107008,140365) | 212504 (188359,241143) | 73 | 10221.9 (9213.7,11321.5) | 9953.9 (8954.8,11078.4) | -2.6 (-4.2,-1.2) |
| South Africa | 3341434 (2931263,3767662) | 5433553 (4783927,6072502) | 63 | 10228.5 (9076.7,11326.5) | 10132.7 (9014,11223.5) | -0.9 (-1.6,-0.4) |
| Eswatini | 67829 (58613,78366) | 101258 (88453,115218) | 49 | 10105 (9079.3,11263.4) | 10148 (9138.5,11250.1) | 0.4 (-0.9,1.8) |
| Zimbabwe | 877771 (761258,1017424) | 1353425 (1178210,1547613) | 54 | 10096.8 (9091.5,11252.6) | 10158.4 (9161.2,11259.5) | 0.6 (-1.3,2.3) |
| Benin | 733690 (607786,880560) | 1855405 (1557969,2183185) | 153 | 15284.1 (13295.1,17262.5) | 14594.5 (12831.1,16441) | -4.5 (-8,-1.3) |
| Burkina Faso | 1377349 (1143506,1652073) | 3136020 (2628669,3685380) | 128 | 14870.4 (13074.3,16770.4) | 14592.2 (12839.5,16493) | -1.9 (-4.7,1.1) |
| Cameroon | 1274696 (1080760,1500692) | 3530601 (3229561,3847104) | 177 | 13058.4 (11589,14723.7) | 12346.8 (11381.3,13515.4) | -5.4 (-13.6,2.3) |
| Cabo Verde | 49159 (42286,57342) | 72612 (63879,82027) | 48 | 14173.1 (12505.1,15987.4) | 13574.5 (11959.8,15250.6) | -4.2 (-7.6,-1.4) |
| Chad | 888782 (734565,1050281) | 2510830 (2090657,2973193) | 183 | 15096.9 (13224.4,17023.3) | 14803 (13023.2,16660.7) | -1.9 (-6.3,1.4) |
| Côte d'Ivoire | 1695753 (1545117,1858210) | 3856510 (3511563,4202935) | 127 | 14798.8 (13593.8,16102.7) | 14732.2 (13600.5,16049.4) | -0.4 (-2.6,1.2) |
| The Gambia | 138618 (114498,163829) | 324302 (272275,376563) | 134 | 14871.9 (13008.4,16613.9) | 14490 (12764.1,16265.7) | -2.6 (-5.8,0) |
| Ghana | 1326278 (1240837,1415201) | 2746681 (2560075,2952858) | 107 | 8661.6 (8090.3,9347.5) | 8309.9 (7729.1,8922.5) | -4.1 (-5.6,-2.5) |
| Guinea | 914727 (757221,1090229) | 1926577 (1617811,2261533) | 111 | 15420.3 (13504,17473.6) | 14918.3 (13085.9,16820.5) | -3.3 (-6.9,0) |
| Guinea-Bissau | 161886 (131163,196002) | 303357 (250888,359749) | 87 | 16045.8 (13959.5,18150.6) | 15360.2 (13490.3,17250.7) | -4.3 (-7.5,-1) |
| Liberia | 373875 (308699,447969) | 723555 (612124,842288) | 94 | 15459.9 (13493.6,17564.3) | 14347.6 (12628.7,16166.2) | -7.2 (-10.8,-3.3) |
| Mali | 2182505 (1858215,2530945) | 6003043 (5084622,6968244) | 175 | 23993.3 (21348.5,26735.9) | 23696.6 (20972.7,26338.7) | -1.2 (-4.6,2) |
| Mauritania | 315413 (262847,374287) | 587571 (502894,690898) | 86 | 15568.2 (13553.9,17664.9) | 14190.7 (12577.6,16070.4) | -8.8 (-12.5,-5.1) |
| Niger | 1471814 (1341699,1601440) | 4131453 (3762671,4539527) | 181 | 18976.3 (17478.2,20659.3) | 17837.7 (16300.9,19503.2) | -6 (-7.7,-4.2) |
| Nigeria | 17595350 (15109347,20275117) | 45466757 (39022952,52447926) | 158 | 20696.5 (18473.2,23279.1) | 20826.8 (18579.1,23380.8) | 0.6 (-0.3,1.5) |
| São Tomé and Príncipe | 17907 (14972,21134) | 28438 (24500,32791) | 59 | 14937.4 (13126.8,16901.5) | 14117 (12462.4,15879.9) | -5.5 (-9.8,-1.9) |
| Senegal | 1124652 (931062,1344289) | 2180866 (1848785,2518183) | 94 | 15133.5 (13260.4,17270.6) | 14565.4 (12801.2,16473.2) | -3.8 (-6.7,-0.7) |
| Sierra Leone | 608775 (510514,715425) | 1212081 (1022336,1423065) | 99 | 15204.3 (13332.8,17101.4) | 14627.6 (12843.7,16522.2) | -3.8 (-7,-0.7) |
| Togo | 534643 (437624,644720) | 1153036 (977419,1338984) | 116 | 15159.5 (13302.1,17214.4) | 14675.4 (12881.5,16558.6) | -3.2 (-6.4,-0.3) |
| American Samoa | 2489 (2196,2805) | 2928 (2612,3275) | 18 | 5953.2 (5318.1,6659.6) | 5921.7 (5288.1,6617) | -0.5 (-1.2,0) |
| Bermuda | 5050 (4553,5658) | 7467 (6723,8346) | 48 | 8374.2 (7588,9302.3) | 8248.3 (7453.6,9184.4) | -1.5 (-2.1,-1) |
| Cook Islands | 1015 (897,1141) | 1122 (1005,1253) | 11 | 5882 (5243,6575.4) | 5742.9 (5122.7,6401.3) | -2.4 (-3.2,-1.6) |
| Greenland | 1242 (1103,1393) | 1498 (1353,1676) | 21 | 2675.3 (2416.5,2986.1) | 2594 (2341.3,2900.6) | -3 (-4.3,-2) |
| Guam | 7204 (6330,8127) | 10015 (8981,11189) | 39 | 5845.2 (5203.5,6534) | 5833.6 (5194.9,6513) | -0.2 (-0.7,0.3) |
| Monaco | 3504 (3176,3936) | 4614 (4183,5178) | 32 | 7800.5 (7035.8,8642.6) | 7741.8 (6969.2,8581.5) | -0.8 (-1.1,-0.4) |
| Nauru | 531 (468,601) | 587 (517,662) | 11 | 6056.9 (5412.8,6763.6) | 5984 (5367,6671.6) | -1.2 (-2.2,-0.4) |
| Niue | 135 (121,150) | 104 (93,117) | -23 | 5935.2 (5312.7,6621.1) | 5874.5 (5250.1,6557) | -1 (-1.8,-0.5) |
| Northern Mariana Islands | 2377 (2069,2706) | 2906 (2591,3273) | 22 | 5891.1 (5262.7,6595.5) | 5848.3 (5221.4,6525) | -0.7 (-1.4,-0.1) |
| Palau | 835 (736,942) | 1141 (1010,1287) | 37 | 5949.8 (5319,6647.4) | 5916.4 (5290.1,6616.6) | -0.6 (-1.4,0.2) |
| Puerto Rico | 303491 (274226,338065) | 399583 (360725,446279) | 32 | 8384.9 (7598.5,9318.2) | 8294.2 (7499.1,9228.9) | -1.1 (-1.6,-0.7) |
| Saint Kitts and Nevis | 3457 (3120,3869) | 5145 (4624,5780) | 49 | 8616.7 (7791.9,9533.3) | 8454.5 (7661.6,9389.9) | -1.9 (-2.7,-1.2) |
| San Marino | 2191 (1984,2432) | 3791 (3444,4235) | 73 | 7789.6 (7024.1,8631.8) | 7733.3 (6963.9,8579.4) | -0.7 (-1.1,-0.4) |
| Tokelau | 89 (80,99) | 82 (73,91) | -8 | 5963.5 (5342.1,6669) | 5864.3 (5231,6536) | -1.7 (-2.6,-1) |
| Tuvalu | 521 (464,582) | 702 (625,788) | 35 | 6052.3 (5414.6,6740.4) | 5955.9 (5332.8,6653.8) | -1.6 (-2.7,-0.6) |
| Virgin Islands | 8193 (7386,9146) | 10085 (9025,11392) | 23 | 8468.1 (7679.5,9412.3) | 8417 (7635,9332) | -0.6 (-1,-0.2) |
| South Sudan | 889952 (742558,1064030) | 1339206 (1125222,1584903) | 50 | 15296.6 (13562.4,17462.4) | 14453.7 (12775.4,16510.9) | -5.5 (-9.1,-2.4) |
| Sudan | 502263 (452479,556729) | 1063705 (954862,1185483) | 112 | 3421.4 (3116.9,3779.9) | 3337.8 (3033.6,3697.8) | -2.4 (-3.4,-1.7) |

| **Table S4.** Comparative analysis of incident cases and age-standardized rate (ASR) changes in fungal skin diseases across 204 nations from 1990 to 2021 | | | | | | |
| --- | --- | --- | --- | --- | --- | --- |
| **Location** | **NO.** | | **percentage change(%)** | **ASRs per 100,000** | | **Percentage change in the ASRs per 100,000(%)** |
|  | **1990 (95%UI)** | **2021 (95%UI)** |  | **1990 (95%UI)** | **2021 (95%UI)** |  |
| China | 162417621 (145294297,182471885) | 229353300 (205924082,254932663) | 41 | 14836.4 (13414.7,16503.5) | 14263 (12845.6,15855.1) | -3.9 (-5,-2.9) |
| North Korea | 2691297 (2425346,3006664) | 3896976 (3524817,4337385) | 45 | 13886.6 (12580.6,15378.7) | 13682.2 (12377,15148.1) | -1.5 (-2.3,-0.9) |
| Taiwan (province of China) | 2507692 (2252571,2807738) | 3700456 (3338704,4096377) | 48 | 13015.8 (11752.7,14462.6) | 12775.2 (11528.8,14227.6) | -1.8 (-2.5,-1.3) |
| Cambodia | 2323782 (2070123,2630712) | 4282307 (3831025,4838193) | 84 | 26950.3 (24245.4,30273.1) | 26674.4 (23983.4,30026.2) | -1 (-1.6,-0.5) |
| Indonesia | 47346548 (41713215,53168071) | 78845408 (69908331,88499425) | 67 | 28594 (25454.6,31730.3) | 28436.9 (25295.2,31552.1) | -0.5 (-0.9,-0.3) |
| Laos | 966107 (860767,1093190) | 1834307 (1633674,2070541) | 90 | 27147.2 (24415.8,30499.1) | 26876.6 (24157.2,30214.8) | -1 (-1.7,-0.5) |
| Malaysia | 4211997 (3742073,4771365) | 8400312 (7517139,9461292) | 99 | 26821 (24119.5,30145.6) | 26566.1 (23842.1,29903.1) | -1 (-1.5,-0.6) |
| Maldives | 48793 (43243,55179) | 137169 (121127,156077) | 181 | 26862.2 (24153.8,30146.2) | 26518.8 (23696.7,29845.4) | -1.3 (-2.3,-0.4) |
| Myanmar | 9730121 (8626076,11014021) | 14731280 (13202375,16629402) | 51 | 27019.1 (24305.4,30325.1) | 26728.1 (24026.2,30064.5) | -1.1 (-1.6,-0.7) |
| Philippines | 15315017 (13522260,17151211) | 29960578 (26709093,33573893) | 96 | 27987 (25109,31177.8) | 27916.1 (25061.3,31098.4) | -0.3 (-0.4,-0.1) |
| Sri Lanka | 4698945 (4217185,5266957) | 6706833 (6061165,7479536) | 43 | 29220.3 (26417.4,32434) | 28844.8 (26057.5,32125.2) | -1.3 (-1.8,-0.9) |
| Thailand | 14070281 (12481034,15945854) | 20408394 (18190530,22936323) | 45 | 26616.6 (23911.9,29938.6) | 26308.4 (23551.2,29628.8) | -1.2 (-1.7,-0.8) |
| Timor-Leste | 176615 (156578,198632) | 335498 (297894,380709) | 90 | 27019.2 (24291.3,30377.7) | 26856.8 (24144.8,30150) | -0.6 (-1,-0.3) |
| Viet Nam | 16013354 (14162759,18128225) | 26822342 (24038332,30154666) | 67 | 26652.7 (23958.7,30001.3) | 26400.1 (23695.3,29738.3) | -0.9 (-1.5,-0.5) |
| Fiji | 125563 (111597,139993) | 167833 (150810,187586) | 34 | 19114.3 (17192.3,21242.8) | 19064.6 (17152.9,21199.8) | -0.3 (-0.7,0.2) |
| Kiribati | 12425 (11096,13826) | 20917 (18738,23322) | 68 | 19439.3 (17463.9,21583.6) | 19292.3 (17343.7,21398.5) | -0.8 (-1.4,0) |
| Marshall Islands | 7113 (6272,7958) | 9808 (8757,10970) | 38 | 19248.3 (17284.7,21374.6) | 19155.6 (17231.5,21292.5) | -0.5 (-1.1,0.1) |
| Federated States of Micronesia | 16878 (14936,18853) | 18144 (16206,20285) | 8 | 19304 (17310.6,21396.5) | 19065.1 (17126.2,21227.3) | -1.2 (-1.9,-0.6) |
| Papua New Guinea | 655269 (581859,732889) | 1725204 (1539294,1935481) | 163 | 19084.6 (17151,21197.9) | 19076.2 (17150.5,21190.5) | 0 (-0.5,0.4) |
| Samoa | 27422 (24286,30677) | 36338 (32492,40420) | 33 | 19111.5 (17172.8,21218.2) | 18926.4 (17003.4,21014.3) | -1 (-1.5,-0.5) |
| Solomon Islands | 53241 (47010,59395) | 113110 (100957,126722) | 112 | 19202.9 (17252.5,21303) | 19055.3 (17163.5,21179.1) | -0.8 (-1.3,-0.3) |
| Tonga | 16078 (14296,17930) | 18471 (16560,20534) | 15 | 18953.3 (17036.4,21093.1) | 18845.4 (16930.8,20948.4) | -0.6 (-1,-0.1) |
| Vanuatu | 24036 (21325,26805) | 52447 (46753,58625) | 118 | 19157.4 (17199.8,21269.2) | 19092.6 (17185.7,21203.9) | -0.3 (-0.8,0.1) |
| Armenia | 553590 (494492,616201) | 641926 (570877,715037) | 16 | 18050.9 (16115.6,19999.2) | 18032.6 (16097.2,19975.6) | -0.1 (-0.2,0) |
| Azerbaijan | 1124263 (1003929,1253501) | 1846978 (1653507,2055929) | 64 | 18058.1 (16137,20002.5) | 18055.4 (16120.1,20004.4) | 0 (-0.3,0.2) |
| Georgia | 1034062 (921407,1151218) | 834699 (742352,931658) | -19 | 18038.8 (16116,19980) | 18039.4 (16109.9,19985.4) | 0 (-0.2,0.1) |
| Kazakhstan | 2622340 (2349219,2920213) | 3249712 (2915584,3604920) | 24 | 18042.6 (16120.8,19981.7) | 18022.1 (16096.4,19965.4) | -0.1 (-0.3,0) |
| Kyrgyzstan | 675057 (601994,752448) | 1059817 (951533,1179738) | 57 | 18055.2 (16131,19997.2) | 18036.6 (16106.9,19986.2) | -0.1 (-0.3,0.1) |
| Mongolia | 297088 (264246,334354) | 511329 (459677,570367) | 72 | 18108.2 (16171.9,20064.8) | 18044.3 (16111,19991.9) | -0.4 (-0.5,-0.2) |
| Tajikistan | 743093 (662465,831508) | 1471019 (1317452,1646195) | 98 | 18082.5 (16138.8,20033.8) | 18084.9 (16155.6,20032.1) | 0 (-0.4,0.3) |
| Turkmenistan | 514286 (458012,577217) | 829443 (745526,918571) | 61 | 18069.1 (16138,20013.5) | 18066.3 (16128.4,20019.8) | 0 (-0.2,0.2) |
| Uzbekistan | 2995954 (2670988,3351434) | 5454395 (4912025,6054349) | 82 | 18061.6 (16124.7,20003.4) | 18058.9 (16122.8,20005.8) | 0 (-0.3,0.2) |
| Albania | 496370 (443841,554921) | 604269 (535013,676708) | 22 | 18075.3 (16130,20039.1) | 18042 (16103.3,19991.3) | -0.2 (-0.5,0.1) |
| Bosnia and Herzegovina | 768425 (690110,854342) | 809701 (714405,906148) | 5 | 18041 (16099.3,19988.7) | 18026.6 (16084.4,19975.4) | -0.1 (-0.2,0) |
| Bulgaria | 1784012 (1579734,1998948) | 1782157 (1565820,2011136) | 0 | 18053 (16118.3,19997.6) | 18028.3 (16086.2,19983.4) | -0.1 (-0.3,0) |
| Croatia | 953102 (847939,1064708) | 1114889 (984075,1252728) | 17 | 18018.9 (16085.4,19960.8) | 18020.1 (16078.6,19971.6) | 0 (-0.2,0.2) |
| Czechia | 2108803 (1874759,2344905) | 2728022 (2410636,3063962) | 29 | 18029.1 (16098.5,19976.7) | 18027 (16082.1,19981.7) | 0 (-0.2,0.2) |
| Hungary | 2174619 (1931539,2421883) | 2483515 (2189805,2781734) | 14 | 18030.7 (16099.9,19978.1) | 18012.6 (16077,19962.1) | -0.1 (-0.2,0) |
| North Macedonia | 345695 (309782,383685) | 468130 (413755,524469) | 35 | 18067.6 (16136.3,20016.9) | 18044.8 (16101.9,19995.5) | -0.1 (-0.3,0) |
| Montenegro | 112092 (100102,124473) | 136036 (120354,152285) | 21 | 18034.6 (16091,19981.7) | 18024.5 (16081.4,19972.6) | -0.1 (-0.1,0) |
| Poland | 7397943 (6603032,8254632) | 9618610 (8492551,10772250) | 30 | 18428.5 (16490.5,20466.2) | 18403.1 (16468.9,20436.8) | -0.1 (-0.3,0) |
| Romania | 4449730 (3953700,4957962) | 4809434 (4250251,5373698) | 8 | 18053.2 (16111.5,20001.3) | 18024.1 (16081.9,19977.8) | -0.2 (-0.3,-0.1) |
| Serbia | 1806453 (1610711,2013567) | 2177295 (1927956,2439071) | 21 | 18050 (16102.6,20000.5) | 18036.3 (16092.4,19985) | -0.1 (-0.2,0) |
| Slovakia | 999401 (886990,1111200) | 1280113 (1132300,1430790) | 28 | 18034.8 (16100.3,19983.6) | 18020 (16081.2,19975.7) | -0.1 (-0.2,0) |
| Slovenia | 393519 (350803,437025) | 554872 (490539,621275) | 41 | 18019.8 (16085.2,19959.4) | 18031.6 (16086,19986.9) | 0.1 (-0.3,0.4) |
| Belarus | 2050974 (1826668,2283676) | 2180007 (1930938,2440399) | 6 | 18022.3 (16101.1,19960.6) | 18008.1 (16085.8,19950.6) | -0.1 (-0.2,0) |
| Estonia | 316638 (281991,351839) | 344954 (304731,385657) | 9 | 18022 (16107.4,19963.3) | 18022.2 (16091.7,19967.7) | 0 (-0.2,0.2) |
| Latvia | 545734 (485960,605938) | 500291 (441509,560699) | -8 | 18023.1 (16104.8,19961.7) | 18018.1 (16092.4,19961) | 0 (-0.2,0.1) |
| Lithuania | 725675 (647645,806216) | 728727 (643772,816627) | 0 | 18029.9 (16103.5,19969) | 18020.8 (16094.2,19964.5) | -0.1 (-0.2,0) |
| Moldova | 775238 (692532,863665) | 830320 (737304,926632) | 7 | 18042 (16116.4,19986.2) | 18028.2 (16097.4,19972) | -0.1 (-0.2,0) |
| Russia | 29489783 (26278196,32901378) | 33793482 (29866735,37810539) | 15 | 18421.2 (16467.4,20484.5) | 18409.5 (16463.2,20452.9) | -0.1 (-0.3,0.1) |
| Ukraine | 10938659 (9691805,12257421) | 10608096 (9356831,11877152) | -3 | 18419.4 (16468.4,20473.4) | 18417.7 (16471.8,20457.3) | 0 (-0.2,0.1) |
| Brunei | 38740 (34373,43430) | 78694 (70096,87589) | 103 | 19427.9 (17379.9,21501.7) | 19175.2 (17157.8,21245.1) | -1.3 (-1.8,-0.9) |
| Japan | 27851218 (24710232,31097552) | 44109283 (38559914,50206552) | 58 | 19516.6 (17443.3,21785.7) | 19346.7 (17293.7,21646.7) | -0.9 (-1.2,-0.6) |
| South Korea | 7381090 (6599888,8217678) | 12996496 (11563248,14535022) | 76 | 19294.9 (17304.1,21350) | 18950.7 (16927.7,21010.4) | -1.8 (-2.4,-1.2) |
| Singapore | 526268 (470032,586139) | 1312912 (1170801,1465798) | 149 | 19166.7 (17167.2,21216.3) | 18939.8 (16905.2,21011.8) | -1.2 (-1.6,-0.8) |
| Australia | 4420439 (3984337,4868066) | 8053935 (7275653,8937386) | 82 | 24531.7 (22121.6,26973.2) | 24358.7 (21960.6,26816.8) | -0.7 (-1,-0.4) |
| New Zealand | 920728 (824005,1030654) | 1616154 (1440268,1807920) | 76 | 25382.4 (22754.1,28310.8) | 25171.8 (22558.7,28102.9) | -0.8 (-1.1,-0.6) |
| Andorra | 13229 (11749,14676) | 26051 (23162,29075) | 97 | 23554.3 (21087.2,26065) | 23318 (20854.6,25815) | -1 (-1.4,-0.7) |
| Austria | 2182497 (1942347,2435326) | 2883453 (2560376,3233875) | 32 | 23550.2 (21062.6,26085.8) | 23318.7 (20860.9,25824.2) | -1 (-1.4,-0.6) |
| Belgium | 2806138 (2497313,3127398) | 3681844 (3272166,4124691) | 31 | 23535.2 (21064.6,26041) | 23317.5 (20870.6,25824.9) | -0.9 (-1.3,-0.6) |
| Cyprus | 184801 (164792,205267) | 379143 (337379,421578) | 105 | 23594.8 (21120.3,26097.6) | 23299.2 (20851.5,25780.2) | -1.3 (-1.7,-0.9) |
| Denmark | 1473160 (1315329,1642318) | 1862125 (1646226,2095181) | 26 | 23591.8 (21120.5,26089.5) | 23343 (20868.8,25843) | -1.1 (-1.5,-0.7) |
| Finland | 1366132 (1216969,1517080) | 1883111 (1660920,2122131) | 38 | 23589 (21081.5,26126.9) | 23325.9 (20872.1,25823.1) | -1.1 (-1.6,-0.7) |
| France | 15834021 (14103952,17572417) | 21708487 (19296928,24400237) | 37 | 23555 (21081.5,26053.1) | 23297.8 (20845.4,25784.4) | -1.1 (-1.5,-0.8) |
| Germany | 22938632 (20426004,25562256) | 29157995 (25807614,32759575) | 27 | 23621.1 (21132,26179.7) | 23351.2 (20901.4,25852.5) | -1.1 (-1.6,-0.7) |
| Greece | 2831618 (2520785,3161962) | 3568146 (3169758,4028583) | 26 | 23443.6 (20987.8,25938) | 23312.9 (20866.8,25796.1) | -0.6 (-0.8,-0.4) |
| Iceland | 62743 (56061,69554) | 101085 (90192,112880) | 61 | 23470.6 (21014.9,25982) | 23289.7 (20840.4,25783.2) | -0.8 (-1.1,-0.5) |
| Ireland | 885292 (792218,985812) | 1401130 (1247675,1565498) | 58 | 23535.3 (21065.1,26044.2) | 23282.1 (20826.4,25768.3) | -1.1 (-1.5,-0.8) |
| Israel | 1427920 (1278326,1595537) | 3020321 (2700629,3377351) | 112 | 29276.5 (26244.2,32712.3) | 29037.7 (26000.4,32511.7) | -0.8 (-1.2,-0.6) |
| Italy | 16584219 (14704569,18485529) | 21906000 (19286296,24684313) | 32 | 24301.8 (21671.3,26963.8) | 24074.7 (21448.9,26735.3) | -0.9 (-1.3,-0.7) |
| Luxembourg | 104240 (92808,115504) | 187202 (166817,207489) | 80 | 23602.5 (21124.5,26132) | 23328.7 (20877.5,25826) | -1.2 (-1.6,-0.8) |
| Malta | 91764 (81965,101584) | 147245 (129800,166044) | 60 | 23524.6 (21060.2,26023.8) | 23324.3 (20861.6,25838.6) | -0.9 (-1.2,-0.6) |
| Netherlands | 3971816 (3537502,4404241) | 5485955 (4845090,6185715) | 38 | 23510.8 (21042.3,26030.6) | 23297.6 (20849.2,25796.3) | -0.9 (-1.3,-0.6) |
| Norway | 1261963 (1122839,1406575) | 1705978 (1516421,1901489) | 35 | 24375.7 (21741.4,27043.8) | 24121.9 (21496.1,26775.2) | -1 (-1.4,-0.7) |
| Portugal | 2687555 (2392047,3001942) | 3668294 (3242357,4145040) | 36 | 23571.1 (21092.4,26073.7) | 23290.2 (20844.9,25780.8) | -1.2 (-1.6,-0.9) |
| Spain | 8360119 (7442301,9288703) | 12502656 (11164090,13997496) | 50 | 18583.8 (16601.5,20460.8) | 18335.2 (16365.8,20223.4) | -1.3 (-1.8,-0.9) |
| Sweden | 2653442 (2370516,2966038) | 3458456 (3062199,3867685) | 30 | 24309.3 (21679.7,26980.2) | 24104.1 (21482.3,26747.6) | -0.8 (-1.2,-0.6) |
| Switzerland | 1937223 (1726521,2154093) | 2867381 (2547481,3213946) | 48 | 23529.6 (21055.2,26042.8) | 23313.4 (20865.1,25815.2) | -0.9 (-1.3,-0.6) |
| UK | 16728155 (14898936,18578521) | 21614427 (19223323,24131053) | 29 | 24227.4 (21627.8,26836.6) | 23997.6 (21388.7,26619.1) | -0.9 (-1.3,-0.7) |
| Argentina | 6235115 (5574873,6903437) | 9598724 (8552740,10634311) | 54 | 19320.8 (17306.7,21374.2) | 19090.7 (17083.4,21134) | -1.2 (-1.6,-0.9) |
| Chile | 2298470 (2060606,2550066) | 4130943 (3682013,4584241) | 80 | 19276.6 (17269.4,21324.6) | 19006 (16996.2,21065.7) | -1.4 (-1.9,-1) |
| Uruguay | 657625 (586432,729284) | 825498 (737023,919795) | 26 | 19243 (17235.8,21285.1) | 19028.1 (17000.9,21077) | -1.1 (-1.5,-0.8) |
| Canada | 2614869 (2337886,2914567) | 4586339 (4069374,5159299) | 75 | 8831.1 (7949.3,9829.1) | 8757.9 (7878.4,9743.2) | -0.8 (-1.2,-0.5) |
| USA | 23546132 (21759507,25188972) | 36663960 (33839814,39273511) | 56 | 8282 (7684.8,8834.6) | 8400.8 (7814.2,8916.2) | 1.4 (-0.3,3.2) |
| Antigua and Barbuda | 14966 (13389,16643) | 24249 (21661,27183) | 62 | 25599.9 (22880.4,28502.2) | 25459.4 (22756.9,28344.6) | -0.5 (-1,-0.3) |
| The Bahamas | 57089 (50844,63556) | 100785 (89531,112823) | 77 | 25816.4 (23096,28707.2) | 25629.9 (22926.1,28524.7) | -0.7 (-1.1,-0.4) |
| Barbados | 69213 (61861,77429) | 95311 (85137,107584) | 38 | 25625.7 (22910.4,28544.8) | 25416.4 (22714.3,28315.3) | -0.8 (-1.2,-0.5) |
| Belize | 39088 (34739,43714) | 97972 (87718,109532) | 151 | 25671.7 (22982.3,28601.4) | 25564.7 (22874.2,28519.3) | -0.4 (-0.7,-0.1) |
| Cuba | 2723887 (2431849,3048353) | 3610912 (3232527,4062522) | 33 | 25457.1 (22750.7,28361.7) | 25244.2 (22558.2,28177.1) | -0.8 (-1.3,-0.5) |
| Dominica | 17400 (15620,19377) | 18691 (16705,20923) | 7 | 25778.6 (23038,28690.6) | 25773.5 (23090.5,28691.6) | 0 (-0.5,0.5) |
| Dominican Republic | 1518846 (1350885,1701314) | 2734357 (2438009,3049757) | 80 | 25804.2 (23138.2,28690.6) | 25618.3 (22922.8,28546.8) | -0.7 (-1.1,-0.4) |
| Grenada | 21080 (18972,23447) | 26985 (24007,30210) | 28 | 25732.6 (23022.5,28702.6) | 25611.5 (22901.3,28521.2) | -0.5 (-0.9,-0.1) |
| Guyana | 165128 (146880,184599) | 184014 (164435,205335) | 11 | 26007.1 (23333.3,28942.8) | 25860.4 (23186,28807) | -0.6 (-1,-0.2) |
| Haiti | 1356634 (1210821,1513834) | 2805608 (2520726,3131081) | 107 | 26225.1 (23477.8,29129.1) | 26013.6 (23353.4,28914) | -0.8 (-1.4,-0.4) |
| Jamaica | 552553 (494979,615654) | 752760 (672744,837114) | 36 | 25564.4 (22862.3,28478.7) | 25493.2 (22817,28385.3) | -0.3 (-0.6,0) |
| Saint Lucia | 30432 (27217,33983) | 51173 (45784,57330) | 68 | 25726 (23003.2,28660.2) | 25532 (22836.4,28429.8) | -0.8 (-1.2,-0.4) |
| Saint Vincent and the Grenadines | 24537 (21852,27478) | 31698 (28344,35415) | 29 | 25693.2 (22976.5,28606.1) | 25618.8 (22929.1,28551.2) | -0.3 (-0.6,0.1) |
| Suriname | 87677 (78635,97790) | 153017 (136850,171110) | 75 | 25904.9 (23208.3,28796.5) | 25731 (23047.8,28647) | -0.7 (-1.1,-0.3) |
| Trinidad and Tobago | 277527 (248790,308465) | 405163 (362331,453266) | 46 | 25853.3 (23154.4,28765.7) | 25628.8 (22929.3,28524.4) | -0.9 (-1.3,-0.5) |
| Bolivia | 1675611 (1495048,1866323) | 3391917 (3030341,3766296) | 102 | 31450.1 (28205.7,34938.5) | 31039.4 (27808.3,34521.2) | -1.3 (-1.9,-0.9) |
| Ecuador | 2647319 (2360241,2948081) | 5414956 (4838183,6024919) | 105 | 31220 (27989.2,34744.5) | 30810.4 (27590.1,34300.2) | -1.3 (-1.8,-0.9) |
| Peru | 5870920 (5196062,6564259) | 11026675 (9836150,12278894) | 88 | 31490.1 (28212,35087.5) | 30964.1 (27667.3,34596.8) | -1.7 (-2.3,-1.2) |
| Colombia | 4947280 (4454505,5490072) | 9346671 (8367805,10367897) | 89 | 18463.4 (16597.8,20404) | 18067.9 (16202,19969.6) | -2.1 (-2.9,-1.5) |
| Costa Rica | 464636 (418256,516492) | 910733 (816093,1012234) | 96 | 18199.9 (16343.2,20107.9) | 18016.1 (16168.7,19929.4) | -1 (-1.5,-0.7) |
| El Salvador | 823012 (738304,912568) | 1166119 (1047969,1288271) | 42 | 18521.1 (16656.9,20464.3) | 18109.6 (16264.3,20034.5) | -2.2 (-3.1,-1.6) |
| Guatemala | 1218686 (1089708,1355068) | 2562830 (2303084,2852127) | 110 | 18703.9 (16803.7,20681) | 18351.3 (16484.8,20294.2) | -1.9 (-2.6,-1.3) |
| Honduras | 691754 (619556,768544) | 1592118 (1431664,1771407) | 130 | 18642.5 (16744.5,20632.9) | 18437.7 (16559,20369.9) | -1.1 (-1.7,-0.7) |
| Mexico | 12301839 (10981009,13644591) | 24763824 (22364841,27470397) | 101 | 17617.6 (15942.8,19531.6) | 19444.6 (17518.4,21611.2) | 10.4 (5.9,14.4) |
| Nicaragua | 551184 (494300,615479) | 1085195 (974140,1207211) | 97 | 18391.4 (16523.6,20326.2) | 18156.4 (16302.5,20026.2) | -1.3 (-1.8,-0.8) |
| Panama | 377415 (339788,419918) | 791115 (710577,874740) | 110 | 18403.4 (16528.9,20328.9) | 18138.7 (16274,20026.1) | -1.4 (-2.2,-1) |
| Venezuela | 2846890 (2559943,3158193) | 5024651 (4515279,5571701) | 76 | 18501.6 (16633.9,20419.1) | 18201 (16352.4,20111.3) | -1.6 (-2.3,-1.1) |
| Brazil | 33754760 (30357571,37479822) | 60942534 (54876447,67703857) | 81 | 26612.5 (24004.4,29478.6) | 26326.7 (23782.6,29124.4) | -1.1 (-1.5,-0.8) |
| Paraguay | 862459 (771915,961712) | 1710677 (1527260,1907455) | 98 | 25686.1 (22996.8,28584.1) | 25528.9 (22826.4,28404) | -0.6 (-1,-0.3) |
| Algeria | 2084041 (1883072,2310587) | 4384047 (3936262,4845571) | 110 | 11384.2 (10251.5,12550.9) | 11074.1 (9976.7,12247.5) | -2.7 (-3.7,-2) |
| Bahrain | 41859 (36970,47093) | 144973 (128670,163454) | 246 | 11379.9 (10256,12567) | 11088.8 (9941.4,12282.8) | -2.6 (-3.5,-1.8) |
| Egypt | 4968460 (4471227,5469557) | 9106487 (8245885,9998301) | 83 | 11904.7 (10776.3,13062.5) | 11128.5 (10044.1,12215.6) | -6.5 (-9.2,-4.4) |
| Iran | 4671405 (4190954,5211788) | 9297241 (8349412,10277825) | 99 | 11557.2 (10435.1,12734.4) | 11248.4 (10126.6,12410.3) | -2.7 (-3.5,-2) |
| Iraq | 1402487 (1258758,1555912) | 3466395 (3076234,3850931) | 147 | 10955.7 (9806.9,12126.8) | 10807.6 (9654.4,11982.8) | -1.4 (-1.7,-1.1) |
| Jordan | 284308 (254828,314577) | 1096088 (978903,1216372) | 286 | 11345.5 (10220.6,12517.1) | 11058.1 (9951.3,12237.7) | -2.5 (-3.4,-1.8) |
| Kuwait | 139974 (123845,157436) | 458231 (407858,514032) | 227 | 11239.7 (10103.7,12413.7) | 11010.1 (9916.9,12194.9) | -2 (-2.8,-1.3) |
| Lebanon | 281329 (253862,311048) | 656562 (590005,727361) | 133 | 11236.2 (10123,12432.1) | 10960.9 (9853.5,12130.3) | -2.5 (-3.3,-1.8) |
| Libya | 341210 (308643,377818) | 688135 (616083,763767) | 102 | 11311.6 (10189.6,12490.2) | 11087.7 (9975.2,12272.2) | -2 (-2.6,-1.4) |
| Morocco | 2247572 (2030175,2473474) | 3915038 (3511009,4334329) | 74 | 11412.3 (10277.8,12620.8) | 11123.5 (10017.2,12308.3) | -2.5 (-3.5,-1.8) |
| Palestine | 159175 (143486,176107) | 418615 (373436,466339) | 163 | 11222.8 (10117.5,12415.9) | 11017.5 (9902.2,12191.8) | -1.8 (-2.6,-1.2) |
| Oman | 156634 (139796,174692) | 411630 (363056,463022) | 163 | 11344.1 (10222.3,12527.9) | 11082.8 (9947.4,12265.5) | -2.3 (-3.1,-1.5) |
| Qatar | 36895 (32264,41842) | 267872 (232064,305136) | 626 | 11428.3 (10246.9,12590.3) | 11102.6 (9921.7,12266.7) | -2.9 (-3.8,-2.1) |
| Saudi Arabia | 1276956 (1139428,1420151) | 3447226 (3053896,3872767) | 170 | 11492.4 (10355.6,12697.4) | 11149.8 (10026.5,12338.5) | -3 (-4,-2.1) |
| Syria | 993380 (892674,1103076) | 1431923 (1284009,1591745) | 44 | 11331.4 (10211.5,12530.4) | 11026.9 (9910.6,12205.9) | -2.7 (-3.5,-2) |
| Tunisia | 731981 (658631,812343) | 1367643 (1223489,1521022) | 87 | 11253.7 (10131.9,12444.8) | 11005 (9900.2,12172.2) | -2.2 (-2.9,-1.6) |
| Türkiye | 5235962 (4717139,5744726) | 9747298 (8747552,10833828) | 86 | 11452.3 (10321,12650.7) | 11074.8 (9977.4,12245.5) | -3.3 (-4.3,-2.4) |
| United Arab Emirates | 154694 (136054,174356) | 950381 (828138,1093303) | 514 | 11570.1 (10389.1,12727.2) | 11344.5 (10150.6,12491.3) | -1.9 (-3.2,-0.6) |
| Yemen | 1029012 (924063,1144367) | 2656465 (2379046,2950639) | 158 | 11372 (10246.7,12556.1) | 11194.9 (10084.2,12401.4) | -1.6 (-2.3,-0.9) |
| Afghanistan | 911391 (820701,1007870) | 2297892 (2055659,2553714) | 152 | 11551.4 (10433.6,12742.6) | 11264.9 (10152.5,12465.6) | -2.5 (-3.4,-1.6) |
| Bangladesh | 18730315 (16353172,21302562) | 28941648 (25891405,32150688) | 55 | 19403.7 (17386.1,21470.6) | 18606.3 (16744.3,20598.6) | -4.1 (-6.2,-2) |
| Bhutan | 103829 (91550,117890) | 132349 (119157,147496) | 27 | 19260.2 (17216.2,21308.5) | 18646.7 (16782.9,20594.7) | -3.2 (-4.6,-1.8) |
| India | 161054541 (139765978,182192618) | 265162118 (235600132,294116264) | 65 | 20501.3 (18224,22762) | 19871.6 (17699.4,21973.9) | -3.1 (-4.4,-1.8) |
| Nepal | 3329806 (2925547,3798780) | 5364144 (4801984,5962107) | 61 | 19328.3 (17294.9,21396.5) | 18753.8 (16871.9,20737.9) | -3 (-5,-1.4) |
| Pakistan | 20080062 (17441849,22780254) | 41337539 (36424661,46462323) | 106 | 19914.8 (17886.9,22004.6) | 19771.2 (17760.2,21904.1) | -0.7 (-1.7,0.1) |
| Angola | 3054319 (2573362,3690138) | 9218538 (7774679,11139227) | 202 | 30876.1 (27080.2,34962.7) | 29821.2 (26260.4,33721.6) | -3.4 (-6.9,0.9) |
| Central African Republic | 821972 (695478,974827) | 1558808 (1341037,1844417) | 90 | 31160.8 (27474.7,35279.7) | 30531.8 (26995.1,34417.7) | -2 (-5.5,1.4) |
| Congo (Brazzaville) | 720429 (615778,855725) | 1470116 (1259987,1708890) | 104 | 31098.8 (27312.3,35231) | 29838.9 (26243.6,33686) | -4.1 (-8.3,-0.9) |
| DR Congo | 11327898 (9500798,13727269) | 24763438 (20883299,29435486) | 119 | 30669.1 (27057.9,34835.7) | 29680.1 (26100.1,33428.6) | -3.2 (-6.3,0.8) |
| Equatorial Guinea | 129283 (109515,156455) | 397291 (337521,467848) | 207 | 31037.5 (27265.3,35141.3) | 29403.4 (25912.2,33097.5) | -5.3 (-8.7,-1.4) |
| Gabon | 292636 (253010,344194) | 481780 (429921,555626) | 65 | 30488.1 (27176.8,34526.4) | 28921.5 (26076.6,32376.2) | -5.1 (-8.4,-1.3) |
| Burundi | 1935746 (1684418,2264173) | 4395881 (3808028,5058524) | 127 | 36838.7 (33038.3,41177.4) | 36419.9 (32523.6,40628.7) | -1.1 (-3.6,1) |
| Comoros | 158551 (138334,181152) | 251775 (222932,284082) | 59 | 36694.5 (32880.5,40867.7) | 36385.3 (32636.8,40726) | -0.8 (-3,1.5) |
| Djibouti | 135714 (117455,155757) | 410354 (361401,463359) | 202 | 36579.3 (32628.7,40758.2) | 36309.7 (32475.8,40511) | -0.7 (-3,1.3) |
| Eritrea | 1163190 (997369,1357586) | 2200811 (1921988,2522963) | 89 | 37002.8 (33130.6,41351.2) | 36821.4 (32807.7,41123.3) | -0.5 (-2.4,1.6) |
| Ethiopia | 26073170 (22545859,30487980) | 49754827 (43552428,56873684) | 91 | 49542.7 (44237,55641.1) | 47244.7 (42242.2,52965.5) | -4.6 (-7.4,-1.9) |
| Kenya | 6924589 (5985962,8078523) | 15523592 (13540467,17719791) | 124 | 32631 (29142.6,36489.9) | 34019 (30222.8,38071.5) | 4.3 (1.1,7.4) |
| Madagascar | 3648654 (3208792,4126458) | 8418110 (7455531,9451256) | 131 | 34316.4 (30813.3,37856.5) | 33885.1 (30535,37337.9) | -1.3 (-2.9,0.3) |
| Malawi | 3331910 (2898615,3861829) | 6376474 (5556527,7304172) | 91 | 36597.3 (32496,40894.4) | 36242.4 (32363.3,40300.8) | -1 (-2.8,1.5) |
| Mauritius | 271813 (241940,307638) | 374163 (333651,420962) | 38 | 26626.2 (23904,29966) | 26504 (23798.6,29814) | -0.5 (-0.7,-0.2) |
| Mozambique | 4401731 (3815110,5011783) | 10015914 (8657667,11459926) | 128 | 35637.9 (31980.8,39724.1) | 35600.5 (32019.7,39616.6) | -0.1 (-2.6,2.5) |
| Rwanda | 2830017 (2442741,3288449) | 4722206 (4084495,5479141) | 67 | 39757 (35441.9,44602.2) | 38271.5 (34035.8,43380.4) | -3.7 (-7.9,0.9) |
| Seychelles | 18340 (16257,20788) | 29308 (26142,32897) | 60 | 26790.5 (24072.6,30151.4) | 26626.1 (23885.7,29981.6) | -0.6 (-1.1,-0.3) |
| Somalia | 2676928 (2297639,3121666) | 7122305 (6120055,8256681) | 166 | 36617.2 (32669.8,40926.9) | 36278.3 (32499.2,40338.1) | -0.9 (-3.3,1.4) |
| Tanzania | 8531434 (7385986,9818093) | 19913225 (17299553,22919603) | 133 | 35777.3 (31923.6,39840.1) | 36738 (32799.3,40847.4) | 2.7 (-0.5,5.7) |
| Uganda | 5759104 (4980021,6616908) | 14215622 (12271104,16485466) | 147 | 36063.7 (32222.3,40021.3) | 36057.2 (32220.3,40278.6) | 0 (-2.2,2.5) |
| Zambia | 2718571 (2348790,3130594) | 6440586 (5578465,7379911) | 137 | 36879.2 (32881.9,41247.5) | 36449.3 (32564,40677) | -1.2 (-3.6,1) |
| Botswana | 322347 (284468,365241) | 620935 (552668,691094) | 93 | 29349.7 (26163.6,32516) | 29234.6 (26022.2,32355.7) | -0.4 (-1.3,0.5) |
| Lesotho | 389417 (344848,436615) | 485853 (429990,542552) | 25 | 29231.6 (26083.1,32370.9) | 29622.9 (26446.8,32799) | 1.3 (0.1,2.4) |
| Namibia | 348001 (308237,391014) | 614568 (548940,687369) | 77 | 29468.8 (26406.1,32588.9) | 29026.4 (25916.6,32118.3) | -1.5 (-2.7,-0.5) |
| South Africa | 9775207 (8682459,10938104) | 16069822 (14308907,17939506) | 64 | 30220.6 (26993.8,33463) | 30022.2 (26817,33238.8) | -0.7 (-1.2,-0.3) |
| Eswatini | 192770 (170198,217743) | 289162 (256985,324692) | 50 | 29292.1 (26206.5,32373.8) | 29390.8 (26189.2,32575.5) | 0.3 (-0.5,1.4) |
| Zimbabwe | 2493285 (2215849,2814333) | 3851234 (3412204,4320539) | 54 | 29229.8 (26148.5,32414.4) | 29365.6 (26260.3,32497.1) | 0.5 (-0.8,1.8) |
| Benin | 1650073 (1426056,1926225) | 4394861 (3803976,5070002) | 166 | 36698.9 (32687.6,41067.1) | 36191.9 (32246.9,40436.7) | -1.4 (-3.3,0.6) |
| Burkina Faso | 3180292 (2746586,3655012) | 7441890 (6453018,8590221) | 134 | 36275.6 (32339.3,40260.1) | 36174.3 (32307.1,40418.4) | -0.3 (-2.3,2.4) |
| Cameroon | 3179485 (2776077,3647739) | 9297579 (8195537,10473955) | 192 | 34055.3 (30530.5,37969.1) | 33389.2 (30036.3,36935.5) | -2 (-7.1,2.5) |
| Cabo Verde | 119573 (104274,136518) | 185655 (164449,208004) | 55 | 35555.9 (31624.1,39719) | 34987.4 (31009.6,38918.2) | -1.6 (-3.9,0.3) |
| Chad | 2035732 (1763013,2352373) | 5803054 (4975671,6750772) | 185 | 36480 (32525.4,40722.6) | 36294.2 (32227.8,40556.3) | -0.5 (-2.7,1.3) |
| Côte d'Ivoire | 3934689 (3400495,4515744) | 9202565 (8087660,10529319) | 134 | 36261.7 (32322.7,40281.4) | 36587.3 (32690.3,40814.1) | 0.9 (-2.9,5) |
| The Gambia | 319260 (274842,367127) | 771903 (673731,877933) | 142 | 36278.3 (32378,40529.3) | 36005.9 (32062.7,40033) | -0.8 (-3.1,1.3) |
| Ghana | 3602209 (3042754,4194233) | 7951132 (6886344,9033215) | 121 | 25870.1 (22880.5,29034.5) | 25513.3 (22778.9,28321.7) | -1.4 (-5.4,3.4) |
| Guinea | 2074877 (1803942,2382428) | 4473889 (3872715,5146731) | 116 | 36760.3 (32664.2,40968.9) | 36487.3 (32413.9,40627.7) | -0.7 (-3,1.3) |
| Guinea-Bissau | 345015 (297703,398543) | 683890 (592719,790212) | 98 | 37184 (33216.5,41520) | 36878.2 (32852.9,41342.5) | -0.8 (-3.3,2) |
| Liberia | 837424 (726152,959563) | 1740736 (1510006,1966063) | 108 | 36740.7 (32819.8,41011.6) | 35873 (31912.7,39939.4) | -2.4 (-4.9,0) |
| Mali | 3662633 (3199209,4236954) | 10099792 (8815661,11703522) | 176 | 43695.9 (38964.3,49001.3) | 43741.4 (39113.8,49030.6) | 0.1 (-2.3,2.8) |
| Mauritania | 705040 (612342,811866) | 1429574 (1248147,1614075) | 103 | 36916.5 (32870.8,41180.1) | 35696.8 (31948.4,39708.6) | -3.3 (-6.2,-0.7) |
| Niger | 3042413 (2641387,3512202) | 9098693 (7999677,10336439) | 199 | 40894.6 (37109,45535) | 39969 (36454.2,44207.3) | -2.3 (-5.1,0.5) |
| Nigeria | 37675384 (33229634,42451802) | 95963470 (83963374,108223361) | 155 | 44489.7 (39660.4,49317.9) | 44547.9 (39673.2,49362.5) | 0.1 (-0.6,0.9) |
| São Tomé and Príncipe | 41263 (35682,47507) | 69853 (61224,78642) | 69 | 36355.5 (32390.4,40645.8) | 35656 (31756.8,39590.7) | -1.9 (-4.2,0.2) |
| Senegal | 2550929 (2203044,2935857) | 5204391 (4528735,5923107) | 104 | 36480.1 (32426.4,40659.1) | 36092 (31973.5,40174.2) | -1.1 (-3.4,1.2) |
| Sierra Leone | 1398461 (1216027,1602083) | 2886598 (2524451,3308656) | 106 | 36558.1 (32545.5,40936.3) | 36228.4 (32442,40432.3) | -0.9 (-2.9,1.2) |
| Togo | 1203282 (1034671,1396423) | 2736677 (2386575,3118401) | 127 | 36543.6 (32407.4,40797.6) | 36250.3 (32183.4,40525) | -0.8 (-3.1,1.3) |
| American Samoa | 7853 (6986,8781) | 9329 (8355,10408) | 19 | 19054.9 (17109.6,21152.3) | 18956.3 (17027.8,21071.5) | -0.5 (-1,-0.1) |
| Bermuda | 15455 (13746,17288) | 22560 (20081,25559) | 46 | 25484 (22756.2,28371.6) | 25137.1 (22450.9,28071.6) | -1.4 (-1.8,-0.9) |
| Cook Islands | 3227 (2869,3604) | 3649 (3259,4065) | 13 | 18899.5 (16983.9,21014.3) | 18546.2 (16657,20672.7) | -1.9 (-2.6,-1.3) |
| Greenland | 4220 (3756,4724) | 5231 (4639,5867) | 24 | 9152.4 (8256.4,10170.2) | 8976.4 (8099,9980.7) | -1.9 (-2.7,-1.2) |
| Guam | 22978 (20494,25846) | 32377 (29115,36045) | 41 | 18811.8 (16910.4,20901) | 18761.5 (16860.8,20852.9) | -0.3 (-0.6,0.1) |
| Monaco | 10496 (9247,11832) | 13718 (12085,15651) | 31 | 23498.9 (21024.3,25989.6) | 23305.9 (20853.3,25797.4) | -0.8 (-1.2,-0.6) |
| Nauru | 1660 (1481,1852) | 1841 (1638,2059) | 11 | 19272.6 (17311,21417.8) | 19070.5 (17145.6,21218.6) | -1 (-1.7,-0.4) |
| Niue | 430 (385,478) | 336 (302,375) | -22 | 19011.1 (17086.2,21116.2) | 18848.8 (16925,20955.8) | -0.9 (-1.4,-0.4) |
| Northern Mariana Islands | 7554 (6680,8569) | 9338 (8373,10469) | 24 | 18923.7 (16964.1,21018.4) | 18798.9 (16899.2,20885.4) | -0.7 (-1.2,-0.2) |
| Palau | 2646 (2361,2966) | 3674 (3286,4125) | 39 | 19046.5 (17132.2,21168.6) | 18958.9 (17050.1,21074.4) | -0.5 (-1,0) |
| Puerto Rico | 922580 (825970,1031402) | 1197602 (1063508,1352276) | 30 | 25491.2 (22784.2,28392.9) | 25251 (22552.4,28170.8) | -0.9 (-1.4,-0.7) |
| Saint Kitts and Nevis | 10372 (9226,11562) | 15765 (14066,17724) | 52 | 25990.2 (23304.3,28937.8) | 25618 (22929.2,28515.3) | -1.4 (-2.1,-0.9) |
| San Marino | 6582 (5874,7336) | 11247 (10007,12640) | 71 | 23466.2 (21009,25975.7) | 23279.9 (20834.7,25758.6) | -0.8 (-1.1,-0.6) |
| Tokelau | 281 (252,311) | 263 (236,293) | -6 | 19069.9 (17144.1,21200.3) | 18827.1 (16922.9,20914.3) | -1.3 (-2,-0.7) |
| Tuvalu | 1642 (1471,1828) | 2232 (1995,2491) | 36 | 19230.2 (17278.5,21384.8) | 19026.6 (17114.5,21159.4) | -1.1 (-1.9,-0.3) |
| Virgin Islands | 25013 (22522,27850) | 30439 (27000,34663) | 22 | 25691.1 (23004.6,28593) | 25534.5 (22834.7,28417.2) | -0.6 (-1,-0.3) |
| South Sudan | 2005154 (1741424,2293592) | 3184057 (2765511,3650371) | 59 | 36713.9 (32814.1,40841.6) | 36052.7 (32216.9,40278.4) | -1.8 (-4.2,0.1) |
| Sudan | 1651621 (1491247,1825620) | 3520066 (3153324,3906371) | 113 | 11434.2 (10305.2,12638.7) | 11195.8 (10080.7,12386.7) | -2.1 (-2.8,-1.5) |

| **Table S5.** Comparative analysis of DALY cases and age-standardized rate (ASR) changes in fungal skin diseases across 204 nations from 1990 to 2021 | | | | | | |
| --- | --- | --- | --- | --- | --- | --- |
| **Location** | **NO.** | | **percentage change(%)** | **ASRs per 100,000** | | **Percentage change in the ASRs per 100,000(%)** |
|  | **1990 (95%UI)** | **2021 (95%UI)** |  | **1990 (95%UI)** | **2021 (95%UI)** |  |
| China | 283873 (114080,594497) | 386453 (157258,805104) | 36 | 25.6 (10.4,53.3) | 24.4 (9.9,50.9) | -4.8 (-6.1,-3.7) |
| North Korea | 4519 (1840,9382) | 6424 (2638,13197) | 42 | 23.1 (9.4,47.4) | 22.8 (9.3,46.7) | -1.4 (-3.1,0.5) |
| Taiwan (province of China) | 4100 (1650,8571) | 5845 (2398,12097) | 43 | 21.1 (8.5,43.9) | 20.7 (8.4,43.1) | -2 (-3.7,-0.3) |
| Cambodia | 5217 (2095,10976) | 9643 (3862,20399) | 85 | 59.3 (24,123.9) | 58.9 (23.8,123.1) | -0.7 (-1.9,0.5) |
| Indonesia | 105930 (43217,221227) | 176817 (71497,364358) | 67 | 62.5 (25.2,128.4) | 62.3 (25.2,127.9) | -0.3 (-0.6,0.2) |
| Laos | 2180 (877,4561) | 4170 (1681,8798) | 91 | 60.2 (24.3,125.6) | 59.7 (24.2,124.4) | -0.8 (-2,0.3) |
| Malaysia | 9517 (3813,20053) | 18845 (7539,40122) | 98 | 59.3 (24,123.8) | 58.7 (23.7,123.6) | -0.9 (-2,0.2) |
| Maldives | 110 (44,231) | 314 (125,673) | 185 | 59.4 (24.1,124.4) | 59 (23.9,124.7) | -0.6 (-2,0.7) |
| Myanmar | 22000 (8793,46479) | 33101 (13249,69624) | 50 | 59.7 (24.1,124.2) | 59.1 (23.7,123.6) | -1 (-2.2,0.1) |
| Philippines | 33382 (13520,69860) | 65367 (26444,136845) | 96 | 59.7 (24.3,123.8) | 59.7 (24.3,124) | 0.1 (-0.2,0.3) |
| Sri Lanka | 12168 (4943,25339) | 16582 (6731,34600) | 36 | 72.8 (29.6,152.1) | 71.8 (29.2,150) | -1.3 (-2.4,-0.4) |
| Thailand | 31926 (12653,68080) | 44431 (18106,92447) | 39 | 58.7 (23.7,123.1) | 58.1 (23.5,121.8) | -1 (-2.1,-0.1) |
| Timor-Leste | 400 (162,842) | 757 (303,1599) | 89 | 59.7 (24.2,124.6) | 59.4 (24,123.9) | -0.5 (-1.5,0.5) |
| Viet Nam | 35856 (14238,75811) | 60035 (24139,126463) | 67 | 58.9 (23.8,123) | 58.5 (23.6,123) | -0.6 (-1.8,0.4) |
| Fiji | 225 (91,462) | 295 (120,607) | 31 | 33.3 (13.5,68) | 33.2 (13.5,67.8) | -0.2 (-1.7,1.4) |
| Kiribati | 23 (9,47) | 38 (15,79) | 65 | 34.2 (14,70.4) | 33.9 (13.9,69.9) | -0.8 (-2.6,0.8) |
| Marshall Islands | 13 (5,27) | 17 (7,36) | 31 | 33.7 (13.7,69) | 33.4 (13.5,68) | -0.9 (-2.4,0.8) |
| Federated States of Micronesia | 31 (13,64) | 32 (13,67) | 3 | 33.9 (13.8,69.2) | 33.2 (13.6,68.2) | -1.9 (-3.6,-0.3) |
| Papua New Guinea | 1172 (478,2444) | 3084 (1254,6345) | 163 | 33.1 (13.5,68.2) | 33.2 (13.5,68.3) | 0.3 (-1.2,1.8) |
| Samoa | 49 (20,103) | 64 (26,133) | 31 | 33.3 (13.5,68.3) | 32.8 (13.3,67.3) | -1.4 (-2.9,0.2) |
| Solomon Islands | 96 (39,201) | 203 (83,422) | 111 | 33.5 (13.7,69) | 33.2 (13.5,68.7) | -0.9 (-2.5,0.6) |
| Tonga | 29 (12,60) | 32 (13,67) | 10 | 33 (13.4,68.2) | 32.7 (13.4,67.3) | -0.8 (-2.1,0.7) |
| Vanuatu | 43 (18,90) | 94 (38,196) | 119 | 33.4 (13.6,68.5) | 33.3 (13.5,68.6) | -0.3 (-1.7,1.2) |
| Armenia | 939 (378,1953) | 1066 (431,2164) | 14 | 30.3 (12.3,62.3) | 30.3 (12.3,62.1) | -0.1 (-1.5,1.2) |
| Azerbaijan | 1919 (774,3989) | 3113 (1243,6437) | 62 | 30.5 (12.4,62.4) | 30.4 (12.3,62.3) | -0.1 (-1.5,1.2) |
| Georgia | 1737 (703,3558) | 1377 (561,2795) | -21 | 30.4 (12.4,62.2) | 30.3 (12.3,61.9) | -0.4 (-1.8,1) |
| Kazakhstan | 4442 (1791,9213) | 5457 (2212,11220) | 23 | 30.3 (12.3,62.1) | 30.2 (12.3,61.8) | -0.2 (-1.4,1.1) |
| Kyrgyzstan | 1152 (468,2371) | 1806 (731,3730) | 57 | 30.4 (12.4,62.1) | 30.4 (12.4,62.1) | 0 (-1.3,1.3) |
| Mongolia | 512 (208,1049) | 869 (351,1774) | 70 | 30.5 (12.4,62.1) | 30.3 (12.3,61.8) | -0.6 (-1.9,0.7) |
| Tajikistan | 1279 (517,2635) | 2523 (1010,5230) | 97 | 30.5 (12.4,62.4) | 30.5 (12.4,62.4) | 0 (-1.3,1.4) |
| Turkmenistan | 885 (357,1838) | 1410 (568,2926) | 59 | 30.4 (12.4,62.4) | 30.5 (12.4,62.4) | 0 (-1.3,1.4) |
| Uzbekistan | 5137 (2087,10603) | 9253 (3740,19071) | 80 | 30.4 (12.4,62.3) | 30.4 (12.4,62) | 0 (-1.4,1.3) |
| Albania | 848 (341,1754) | 1000 (407,2037) | 18 | 30.4 (12.3,62) | 30.4 (12.4,62.1) | -0.1 (-1.4,1.2) |
| Bosnia and Herzegovina | 1294 (518,2672) | 1323 (538,2687) | 2 | 30.3 (12.3,61.9) | 30.2 (12.2,61.7) | -0.3 (-1.6,1.1) |
| Bulgaria | 2955 (1207,6041) | 2911 (1195,5954) | -1 | 30.3 (12.4,62.1) | 30.3 (12.3,62.3) | -0.1 (-1.5,1.2) |
| Croatia | 1585 (644,3220) | 1817 (743,3679) | 15 | 30.2 (12.4,61.7) | 30.2 (12.3,61.9) | 0 (-1.2,1.3) |
| Czechia | 3482 (1414,7112) | 4445 (1820,9029) | 28 | 30.1 (12.2,61.5) | 30.2 (12.2,61.6) | 0.2 (-1.2,1.4) |
| Hungary | 3578 (1462,7268) | 4057 (1653,8227) | 13 | 30 (12.3,61.3) | 30.2 (12.2,61.3) | 0.5 (-0.9,1.8) |
| North Macedonia | 582 (235,1202) | 772 (315,1572) | 33 | 30.3 (12.3,61.9) | 30.2 (12.3,62) | -0.3 (-1.6,1) |
| Montenegro | 189 (77,386) | 224 (92,457) | 19 | 30.3 (12.4,61.9) | 30.2 (12.3,61.8) | -0.3 (-1.7,0.9) |
| Poland | 12384 (5047,25555) | 15852 (6475,32115) | 28 | 31 (12.7,63.9) | 31.1 (12.7,64) | 0.2 (-0.2,0.7) |
| Romania | 7421 (3013,15277) | 7909 (3222,16125) | 7 | 30.3 (12.3,61.8) | 30.3 (12.3,62.2) | 0.1 (-1.4,1.4) |
| Serbia | 3021 (1209,6158) | 3577 (1456,7269) | 18 | 30.3 (12.3,61.9) | 30.3 (12.2,61.9) | -0.2 (-1.7,1.2) |
| Slovakia | 1665 (670,3433) | 2100 (856,4262) | 26 | 30.2 (12.2,62.1) | 30.2 (12.2,61.4) | 0.1 (-1.3,1.4) |
| Slovenia | 652 (264,1333) | 904 (370,1822) | 39 | 30.1 (12.2,61.6) | 30.2 (12.2,61.9) | 0.4 (-1,1.8) |
| Belarus | 3417 (1383,6971) | 3584 (1453,7226) | 5 | 30.2 (12.3,61.8) | 30.2 (12.2,61.5) | -0.2 (-1.5,1.1) |
| Estonia | 525 (214,1067) | 565 (231,1143) | 8 | 30.2 (12.3,61.6) | 30.3 (12.2,61.7) | 0.2 (-1.1,1.4) |
| Latvia | 902 (368,1842) | 817 (333,1652) | -9 | 30.2 (12.3,61.9) | 30.2 (12.2,61.6) | 0.1 (-1.4,1.6) |
| Lithuania | 1207 (492,2470) | 1189 (486,2393) | -1 | 30.2 (12.3,62) | 30.2 (12.3,61.8) | -0.1 (-1.5,1.3) |
| Moldova | 1302 (528,2680) | 1370 (559,2774) | 5 | 30.3 (12.3,62.1) | 30.3 (12.4,61.5) | 0.1 (-1.3,1.3) |
| Russia | 49356 (20119,101896) | 55806 (22685,113388) | 13 | 31 (12.7,64) | 31 (12.7,64) | -0.1 (-0.4,0.3) |
| Ukraine | 18222 (7429,37596) | 17483 (7122,35512) | -4 | 31 (12.7,64.4) | 31.1 (12.7,64) | 0.1 (-1.2,1.4) |
| Brunei | 68 (28,140) | 134 (55,275) | 97 | 33.3 (13.7,68.2) | 32.6 (13.4,66.3) | -1.9 (-3.4,-0.5) |
| Japan | 47240 (19115,95533) | 74067 (30184,149065) | 57 | 33.4 (13.5,67.1) | 33.2 (13.4,66.7) | -0.8 (-1.2,-0.3) |
| South Korea | 12665 (5134,26055) | 21569 (8883,44407) | 70 | 32.8 (13.4,66.7) | 32 (13.2,65.2) | -2.3 (-3.9,-0.8) |
| Singapore | 899 (367,1876) | 2207 (911,4570) | 145 | 32.6 (13.4,67.1) | 32.2 (13.3,66) | -1.3 (-2.6,0) |
| Australia | 8624 (3571,17645) | 16106 (6629,32666) | 87 | 47.8 (19.8,98.4) | 47.6 (19.5,98) | -0.5 (-1.5,0.6) |
| New Zealand | 1754 (731,3577) | 3123 (1302,6371) | 78 | 48.3 (20,98.2) | 48.1 (20,98.2) | -0.4 (-1.3,0.5) |
| Andorra | 24 (10,51) | 47 (19,99) | 96 | 43.4 (17.8,89.5) | 42.9 (17.6,88) | -1.3 (-2.5,-0.1) |
| Austria | 3955 (1631,8181) | 5230 (2148,10804) | 32 | 43.1 (17.8,88.7) | 42.8 (17.5,87.7) | -0.8 (-1.9,0.3) |
| Belgium | 5097 (2103,10537) | 6675 (2758,13811) | 31 | 43.2 (17.8,88.5) | 42.7 (17.6,87.3) | -1 (-2.1,0.2) |
| Cyprus | 339 (140,701) | 690 (285,1422) | 104 | 43.4 (17.9,89.1) | 42.8 (17.7,87.7) | -1.3 (-2.5,0) |
| Denmark | 2680 (1112,5542) | 3399 (1402,7069) | 27 | 43.4 (17.9,89.2) | 43 (17.7,88.6) | -0.9 (-2.2,0.3) |
| Finland | 2473 (1019,5083) | 3408 (1406,7088) | 38 | 43.1 (17.8,88.1) | 42.7 (17.6,87.6) | -1 (-2.2,0.2) |
| France | 28785 (11862,59236) | 39409 (16250,81729) | 37 | 43.2 (17.7,88.4) | 42.7 (17.6,87.3) | -1.1 (-2.2,0.1) |
| Germany | 41560 (17010,85528) | 52716 (21675,109586) | 27 | 43.3 (17.7,88.6) | 42.8 (17.6,87.8) | -1.2 (-2.5,0.2) |
| Greece | 5163 (2122,10728) | 6487 (2672,13426) | 26 | 43.1 (17.6,88.5) | 42.8 (17.6,88.2) | -0.5 (-1.6,0.5) |
| Iceland | 115 (47,238) | 185 (76,385) | 61 | 43.2 (17.8,88.9) | 42.9 (17.5,88.3) | -0.6 (-1.8,0.5) |
| Ireland | 1626 (667,3333) | 2557 (1052,5305) | 57 | 43.3 (17.7,88.5) | 42.8 (17.6,88) | -1.1 (-2.4,0.2) |
| Israel | 3374 (1393,7007) | 7121 (2951,14747) | 111 | 69.3 (28.5,143.6) | 68.7 (28.4,142.4) | -0.8 (-1.7,0.2) |
| Italy | 29580 (12265,61414) | 38996 (16151,80869) | 32 | 43.8 (18,90.1) | 43.5 (17.8,89.7) | -0.7 (-1.2,-0.2) |
| Luxembourg | 189 (78,389) | 341 (141,705) | 80 | 43.3 (17.8,88.5) | 42.9 (17.7,87.9) | -1 (-2.3,0.2) |
| Malta | 168 (69,348) | 268 (110,562) | 60 | 43.3 (17.8,89.1) | 42.9 (17.7,88) | -1 (-2.1,0.3) |
| Netherlands | 7258 (2990,14963) | 9974 (4097,20785) | 37 | 43.2 (17.7,88.7) | 42.8 (17.5,87.8) | -1 (-2,0.2) |
| Norway | 2256 (938,4676) | 3058 (1269,6338) | 36 | 44 (18.1,90.5) | 43.7 (17.9,90) | -0.8 (-1.4,-0.2) |
| Portugal | 4899 (2010,10150) | 6649 (2739,13878) | 36 | 43.3 (17.8,88.3) | 42.7 (17.6,87.8) | -1.3 (-2.5,0) |
| Spain | 13212 (5435,27126) | 19651 (8108,40321) | 49 | 29.6 (12.2,60.8) | 29.1 (12,59.9) | -1.5 (-3.1,-0.2) |
| Sweden | 4757 (1979,9877) | 6202 (2573,12918) | 30 | 44.1 (18,90.5) | 43.7 (17.9,90.1) | -0.9 (-1.9,0.1) |
| Switzerland | 3510 (1448,7233) | 5201 (2140,10776) | 48 | 43 (17.7,88.3) | 42.7 (17.6,87.8) | -0.7 (-1.8,0.4) |
| UK | 30073 (12488,62435) | 38759 (16077,80526) | 29 | 44 (18.1,90.4) | 43.5 (17.9,89.5) | -1.1 (-1.5,-0.7) |
| Argentina | 10604 (4376,21633) | 16141 (6632,32893) | 52 | 32.8 (13.6,66.9) | 32.3 (13.2,65.7) | -1.7 (-3,-0.3) |
| Chile | 3917 (1605,8032) | 6887 (2852,14115) | 76 | 32.6 (13.4,66.5) | 32 (13.2,65.2) | -2.1 (-3.7,-0.7) |
| Uruguay | 1108 (457,2275) | 1378 (569,2808) | 24 | 32.6 (13.4,66.8) | 32.1 (13.2,65.1) | -1.6 (-2.9,-0.3) |
| Canada | 4083 (1657,8402) | 6980 (2821,14196) | 71 | 13.9 (5.7,28.7) | 13.7 (5.6,27.8) | -1.4 (-3.5,0.7) |
| USA | 38907 (15934,79887) | 58556 (24201,118692) | 51 | 13.8 (5.6,28.3) | 13.7 (5.6,28) | -0.7 (-2,0.5) |
| Antigua and Barbuda | 27 (11,57) | 44 (18,90) | 63 | 46.6 (19.4,96.4) | 46.2 (19.2,95.5) | -0.9 (-1.9,0.4) |
| The Bahamas | 105 (43,220) | 183 (75,380) | 74 | 47.3 (19.7,97.6) | 46.8 (19.4,96.8) | -1.1 (-2.2,0) |
| Barbados | 127 (52,261) | 172 (71,354) | 35 | 46.8 (19.4,96.7) | 46.2 (19.1,95.6) | -1.2 (-2.3,-0.2) |
| Belize | 73 (30,150) | 180 (74,375) | 147 | 47 (19.6,97.3) | 46.7 (19.3,96.4) | -0.7 (-1.8,0.5) |
| Cuba | 4965 (2049,10286) | 6496 (2657,13432) | 31 | 46.4 (19.3,95.5) | 45.8 (19,94.8) | -1.2 (-2.3,-0.1) |
| Dominica | 32 (13,66) | 34 (14,70) | 6 | 47.1 (19.6,98) | 47.1 (19.5,97.3) | -0.1 (-1.3,1.3) |
| Dominican Republic | 2814 (1161,5863) | 4987 (2061,10349) | 77 | 47.3 (19.6,98.1) | 46.7 (19.4,96.8) | -1.2 (-2.4,0.1) |
| Grenada | 39 (16,80) | 49 (20,102) | 26 | 47 (19.5,96.7) | 46.6 (19.2,96.8) | -0.8 (-2,0.3) |
| Guyana | 306 (126,637) | 335 (138,698) | 9 | 47.5 (19.6,97.9) | 47.1 (19.5,97.4) | -0.8 (-2,0.5) |
| Haiti | 2535 (1041,5250) | 5195 (2147,10901) | 105 | 48.2 (19.9,99.5) | 47.7 (19.8,98.9) | -1 (-2.3,0.3) |
| Jamaica | 1018 (423,2105) | 1370 (565,2851) | 35 | 46.7 (19.4,96.5) | 46.5 (19.3,96.6) | -0.4 (-1.4,0.7) |
| Saint Lucia | 56 (23,117) | 92 (38,191) | 64 | 46.8 (19.4,96.8) | 46.4 (19.2,95.8) | -0.9 (-2,0.2) |
| Saint Vincent and the Grenadines | 45 (19,94) | 57 (24,119) | 27 | 46.8 (19.4,96.2) | 46.6 (19.3,96.5) | -0.4 (-1.6,0.7) |
| Suriname | 162 (66,340) | 277 (115,577) | 71 | 47.4 (19.6,98.5) | 46.9 (19.5,97) | -1.3 (-2.6,0) |
| Trinidad and Tobago | 511 (211,1067) | 730 (301,1519) | 43 | 47.2 (19.6,97.7) | 46.6 (19.4,96.6) | -1.3 (-2.4,0) |
| Bolivia | 3648 (1487,7489) | 7341 (2987,15261) | 101 | 67.9 (27.8,141.2) | 67 (27.4,139.5) | -1.4 (-2.5,-0.3) |
| Ecuador | 5774 (2359,11799) | 11712 (4771,24299) | 103 | 67.6 (27.6,140.2) | 66.5 (27.1,138.3) | -1.6 (-2.6,-0.5) |
| Peru | 13119 (5360,27635) | 24299 (9972,51033) | 85 | 69.4 (28.6,145.3) | 68.2 (28.1,143) | -1.6 (-2.7,-0.6) |
| Colombia | 8348 (3394,17176) | 15166 (6198,31282) | 82 | 30.4 (12.3,62.6) | 29.5 (12,60.7) | -2.8 (-4.2,-1.2) |
| Costa Rica | 777 (316,1595) | 1472 (600,3042) | 89 | 29.8 (12.1,61.3) | 29.3 (12,60.9) | -1.4 (-2.9,0) |
| El Salvador | 1393 (569,2872) | 1907 (780,3954) | 37 | 30.5 (12.4,62.7) | 29.6 (12.1,61.2) | -3 (-4.7,-1.3) |
| Guatemala | 2109 (860,4420) | 4271 (1759,8791) | 103 | 30.9 (12.7,63.6) | 30 (12.4,62) | -2.7 (-4.3,-1.1) |
| Honduras | 1194 (487,2475) | 2682 (1103,5560) | 125 | 30.8 (12.6,63.4) | 30.4 (12.4,62.6) | -1.5 (-3,0.1) |
| Mexico | 21195 (8693,43388) | 41863 (17035,85598) | 98 | 29.2 (12.2,59.3) | 32.8 (13.5,66.9) | 12.3 (7.9,16.8) |
| Nicaragua | 941 (384,1959) | 1793 (733,3694) | 91 | 30.1 (12.3,61.9) | 29.6 (12,60.8) | -1.7 (-3.3,-0.2) |
| Panama | 633 (259,1303) | 1291 (528,2663) | 104 | 30.2 (12.4,62.4) | 29.6 (12.1,61.1) | -2 (-3.6,-0.6) |
| Venezuela | 4823 (1972,9976) | 8186 (3324,16714) | 70 | 30.5 (12.5,63) | 29.8 (12.1,61.2) | -2.1 (-3.7,-0.6) |
| Brazil | 61503 (25424,125545) | 108572 (44952,223665) | 77 | 47.8 (19.9,97.8) | 47.2 (19.6,96.4) | -1.3 (-1.8,-0.8) |
| Paraguay | 1590 (661,3292) | 3115 (1283,6493) | 96 | 46.8 (19.5,96.9) | 46.4 (19.2,95.8) | -0.9 (-1.9,0.1) |
| Algeria | 3533 (1418,7272) | 7182 (2929,14489) | 103 | 18.7 (7.6,37.9) | 18 (7.3,36.3) | -3.8 (-5.6,-2) |
| Bahrain | 71 (28,144) | 239 (97,486) | 237 | 18.6 (7.5,37.8) | 17.9 (7.3,36.2) | -3.9 (-5.8,-1.8) |
| Egypt | 8978 (3654,18318) | 15121 (6150,31179) | 68 | 20.3 (8.4,41.3) | 18 (7.4,36.5) | -11.2 (-14.2,-8.2) |
| Iran | 8080 (3281,16500) | 15420 (6359,31276) | 91 | 19.2 (7.9,38.7) | 18.5 (7.6,37.6) | -3.4 (-4.4,-2.4) |
| Iraq | 2307 (935,4717) | 5645 (2294,11494) | 145 | 17.6 (7.2,35.6) | 17.2 (7.1,35) | -2 (-3.6,-0.1) |
| Jordan | 484 (196,991) | 1809 (730,3729) | 274 | 18.6 (7.6,37.6) | 17.9 (7.3,36.5) | -3.7 (-5.6,-1.7) |
| Kuwait | 235 (95,481) | 751 (301,1542) | 220 | 18.3 (7.5,37.2) | 17.8 (7.3,36.2) | -3 (-5.1,-1.1) |
| Lebanon | 464 (190,944) | 1051 (428,2132) | 127 | 18.2 (7.5,36.8) | 17.6 (7.2,36) | -3.4 (-5.3,-1.6) |
| Libya | 575 (231,1171) | 1126 (457,2286) | 96 | 18.5 (7.5,37.5) | 17.9 (7.3,36.2) | -2.9 (-4.8,-0.8) |
| Morocco | 3786 (1541,7746) | 6376 (2629,12956) | 68 | 18.7 (7.7,38.1) | 18 (7.4,36.4) | -3.8 (-5.7,-1.7) |
| Palestine | 268 (109,547) | 695 (282,1425) | 159 | 18.3 (7.5,37) | 17.8 (7.3,36) | -2.6 (-4.4,-0.8) |
| Oman | 265 (107,538) | 686 (275,1418) | 159 | 18.4 (7.5,37.3) | 17.9 (7.3,36.5) | -2.8 (-4.8,-0.9) |
| Qatar | 62 (25,126) | 445 (177,910) | 618 | 18.6 (7.6,37.7) | 17.8 (7.3,36.2) | -4.1 (-6.1,-2.3) |
| Saudi Arabia | 2180 (884,4468) | 5711 (2303,11610) | 162 | 18.8 (7.7,38.2) | 18 (7.4,36.2) | -4.1 (-5.9,-2.3) |
| Syria | 1684 (674,3454) | 2329 (952,4760) | 38 | 18.5 (7.6,37.8) | 17.8 (7.3,36.2) | -3.7 (-5.6,-1.7) |
| Tunisia | 1221 (493,2500) | 2204 (897,4481) | 81 | 18.4 (7.5,37.1) | 17.8 (7.3,36.3) | -3.2 (-4.9,-1.5) |
| Türkiye | 8828 (3612,18233) | 15805 (6446,32149) | 79 | 18.9 (7.7,38.4) | 18 (7.3,36.5) | -4.6 (-6.3,-2.7) |
| United Arab Emirates | 265 (106,538) | 1583 (636,3257) | 497 | 19 (7.7,38.3) | 18.5 (7.6,37.6) | -2.7 (-4.9,-0.7) |
| Yemen | 1759 (707,3597) | 4494 (1830,9150) | 155 | 18.5 (7.6,37.5) | 18.2 (7.5,36.9) | -1.6 (-3.4,0.2) |
| Afghanistan | 1541 (633,3146) | 3949 (1598,8037) | 156 | 19 (7.8,38.2) | 18.3 (7.5,37.1) | -3.7 (-5.9,-1.7) |
| Bangladesh | 37084 (14736,80425) | 51670 (21191,106835) | 39 | 35.7 (14.5,73.6) | 32.9 (13.4,68.1) | -7.9 (-11.7,-4.7) |
| Bhutan | 204 (82,437) | 236 (97,478) | 16 | 35.2 (14.3,73.2) | 33 (13.5,67.5) | -6.2 (-9.2,-3) |
| India | 314340 (127202,671810) | 485519 (197480,994783) | 54 | 37.9 (15.6,79) | 36 (14.7,74.3) | -4.8 (-6.9,-3.1) |
| Nepal | 6470 (2588,13809) | 9705 (3998,19786) | 50 | 35.2 (14.3,72.9) | 33.3 (13.7,67.8) | -5.4 (-8.7,-2.2) |
| Pakistan | 38213 (15409,81384) | 77693 (31207,161655) | 103 | 36 (14.6,75.4) | 35.6 (14.3,73.5) | -0.9 (-2.4,0.6) |
| Angola | 9267 (3642,20152) | 25292 (10161,55876) | 173 | 78.5 (31.6,168.7) | 68.8 (28.3,148.1) | -12.4 (-17.9,-6.4) |
| Central African Republic | 2514 (1020,5441) | 4522 (1826,9829) | 80 | 80.2 (32.8,172.8) | 75.4 (30.8,160.6) | -6 (-11.7,-0.9) |
| Congo (Brazzaville) | 2208 (889,4791) | 3786 (1519,8063) | 71 | 80 (32.3,174.3) | 69.2 (28.3,147.5) | -13.6 (-18.9,-7.4) |
| DR Congo | 33597 (13426,73146) | 65018 (26540,140063) | 94 | 76.2 (30.6,164.1) | 67.4 (27.6,143.6) | -11.5 (-16.8,-5.3) |
| Equatorial Guinea | 390 (157,839) | 1008 (407,2175) | 158 | 78.7 (32.1,166.5) | 65.2 (26.6,139.6) | -17.2 (-23.1,-11.2) |
| Gabon | 826 (338,1723) | 1189 (485,2446) | 44 | 75.3 (30.9,155.1) | 65.1 (26.7,133.1) | -13.6 (-15.2,-11.9) |
| Burundi | 5033 (2023,10717) | 10816 (4433,23009) | 115 | 87 (36.4,183.8) | 83.1 (34.6,176.4) | -4.5 (-8,-1.3) |
| Comoros | 405 (166,862) | 592 (247,1261) | 46 | 85.7 (35.5,180.1) | 83 (34.5,176) | -3.2 (-6.5,0) |
| Djibouti | 340 (139,714) | 964 (399,2010) | 184 | 84.6 (35.3,175.3) | 82.3 (34.1,172.4) | -2.7 (-6.3,0.9) |
| Eritrea | 3055 (1221,6469) | 5454 (2239,11543) | 79 | 87.3 (36,181.6) | 85.2 (35.3,177) | -2.4 (-6,1.5) |
| Ethiopia | 87523 (35406,179991) | 146226 (59500,304555) | 67 | 148.4 (60.3,307.9) | 129.3 (52.4,270.1) | -12.9 (-16.1,-9.7) |
| Kenya | 14268 (5802,30763) | 31908 (13144,67341) | 124 | 62.5 (25.6,130) | 66.2 (27.1,137.9) | 6 (2.8,9.3) |
| Madagascar | 8061 (3275,16431) | 18283 (7506,37439) | 127 | 72.9 (30.1,150.6) | 71.2 (29.4,146.4) | -2.4 (-4.2,-0.6) |
| Malawi | 8426 (3438,17946) | 15488 (6341,32872) | 84 | 84.9 (35,177.7) | 81.8 (34,173.6) | -3.7 (-7.1,-0.4) |
| Mauritius | 612 (244,1302) | 817 (332,1713) | 33 | 58.7 (23.7,122.6) | 58.3 (23.7,122) | -0.6 (-1.6,0.4) |
| Mozambique | 10255 (4238,21210) | 23212 (9557,48354) | 126 | 78 (32.2,162.6) | 77.2 (31.9,162) | -1.1 (-3,0.8) |
| Rwanda | 9600 (3890,19817) | 12563 (5086,25781) | 31 | 114.4 (46.4,233.6) | 94.2 (38.4,194) | -17.6 (-20.5,-13.5) |
| Seychelles | 41 (16,87) | 65 (26,137) | 59 | 59.4 (24,124.3) | 58.9 (23.8,123.9) | -0.8 (-2,0.5) |
| Somalia | 6821 (2731,14615) | 17501 (7063,36630) | 157 | 84.6 (34.9,180.1) | 81.6 (34.3,171.1) | -3.5 (-7,0) |
| Tanzania | 20407 (8382,42551) | 49083 (19641,101466) | 141 | 80.1 (32.9,166.2) | 84.6 (34.3,175.9) | 5.7 (0.6,9.6) |
| Uganda | 14079 (5734,30016) | 34428 (14055,74089) | 145 | 81.3 (34,171) | 80.7 (33.3,168) | -0.8 (-4.1,2.5) |
| Zambia | 7102 (2865,14974) | 15863 (6474,33468) | 123 | 87.3 (36.4,184.2) | 83 (34.4,173.9) | -4.9 (-8.9,-1.5) |
| Botswana | 640 (261,1341) | 1201 (488,2520) | 88 | 56.3 (22.8,115.2) | 55.6 (22.5,114.8) | -1.3 (-2.8,0.2) |
| Lesotho | 766 (312,1592) | 955 (389,1988) | 25 | 55.9 (22.7,114.2) | 56.6 (22.9,115.9) | 1.3 (-0.7,3.4) |
| Namibia | 692 (283,1446) | 1190 (484,2492) | 72 | 56.7 (23.1,116.2) | 55 (22.4,113.2) | -3 (-4.9,-1.2) |
| South Africa | 18681 (7577,38882) | 30036 (12032,62423) | 61 | 56.5 (22.9,116.9) | 55.7 (22.5,115.3) | -1.4 (-2.1,-0.7) |
| Eswatini | 384 (155,807) | 567 (231,1196) | 48 | 56.1 (22.7,115.2) | 55.8 (22.6,115.1) | -0.5 (-2.1,1.5) |
| Zimbabwe | 4953 (2026,10386) | 7617 (3094,15989) | 54 | 56 (22.8,115.6) | 56.2 (22.6,115.6) | 0.3 (-2,2.5) |
| Benin | 4148 (1645,8792) | 10505 (4267,22073) | 153 | 85.1 (34.1,179.6) | 81.4 (32.8,171) | -4.3 (-7.9,-0.9) |
| Burkina Faso | 7757 (3160,16371) | 17752 (7147,37430) | 129 | 82.6 (33.5,173.1) | 81.5 (33,172.7) | -1.3 (-4.5,1.7) |
| Cameroon | 7196 (2919,15277) | 19951 (8074,41683) | 177 | 72.6 (29.8,152.8) | 68.7 (28.1,142) | -5.4 (-13.5,2.9) |
| Cabo Verde | 277 (112,589) | 407 (166,860) | 47 | 79.3 (32.1,166) | 75.8 (30.8,158.8) | -4.4 (-7.6,-1.7) |
| Chad | 5013 (2042,10811) | 14223 (5779,30048) | 184 | 84 (34.4,176) | 82.5 (33.8,172.7) | -1.8 (-6.2,1.8) |
| Côte d'Ivoire | 9558 (3876,19944) | 21809 (8915,45553) | 128 | 81.9 (33.2,169.8) | 82.1 (33.4,170) | 0.3 (-2.2,2.1) |
| The Gambia | 785 (318,1676) | 1832 (755,3934) | 133 | 83 (33.9,177.4) | 80.7 (32.9,169.6) | -2.7 (-6.1,0.5) |
| Ghana | 7502 (3080,15294) | 15540 (6429,32035) | 107 | 48.3 (19.9,98.3) | 46.5 (19.3,94.9) | -3.9 (-5.9,-2.1) |
| Guinea | 5162 (2085,10985) | 10905 (4455,23173) | 111 | 86 (35.9,182.2) | 83.3 (34.4,175.2) | -3.2 (-6.8,0.2) |
| Guinea-Bissau | 916 (368,1952) | 1720 (704,3659) | 88 | 89.4 (36.4,187.9) | 85.8 (35.5,180) | -4 (-7.7,-0.8) |
| Liberia | 2100 (837,4481) | 4072 (1661,8708) | 94 | 85.6 (34.3,180) | 79.5 (32.3,165.9) | -7.1 (-10.9,-3.2) |
| Mali | 12326 (4971,26084) | 34017 (13761,72060) | 176 | 133.9 (54.2,277.9) | 132.5 (54.3,273.6) | -1 (-4.5,2.5) |
| Mauritania | 1784 (711,3841) | 3324 (1359,7034) | 86 | 87 (35.1,184.6) | 79.3 (32.2,165.2) | -8.8 (-12.5,-4.8) |
| Niger | 8321 (3329,17356) | 23420 (9604,49340) | 181 | 105.7 (43.2,220.3) | 99.5 (40.6,208) | -5.8 (-7.6,-4) |
| Nigeria | 98883 (40273,208790) | 256949 (104358,544387) | 160 | 114.9 (47.3,240.7) | 116.1 (47.7,242.5) | 1.1 (0.1,1.9) |
| São Tomé and Príncipe | 101 (40,217) | 161 (66,337) | 59 | 83.6 (33.7,175.5) | 78.9 (32.3,165.5) | -5.6 (-10.1,-1.9) |
| Senegal | 6345 (2568,13486) | 12318 (5041,26896) | 94 | 84.2 (34.6,177.4) | 81.2 (33.4,172.5) | -3.5 (-6.7,0) |
| Sierra Leone | 3429 (1394,7358) | 6847 (2800,14801) | 100 | 84.6 (34.9,177.2) | 81.5 (33.1,172.8) | -3.6 (-7,-0.3) |
| Togo | 3024 (1219,6474) | 6519 (2632,13738) | 116 | 84.4 (34.4,178.8) | 81.9 (33.3,172.5) | -3 (-6.4,0.1) |
| American Samoa | 14 (6,29) | 16 (7,34) | 14 | 33.1 (13.6,67.8) | 32.8 (13.4,67.5) | -1 (-2.5,0.5) |
| Bermuda | 28 (12,58) | 41 (17,84) | 46 | 46.5 (19.3,96.2) | 45.7 (18.9,94.2) | -1.8 (-3,-0.6) |
| Cook Islands | 6 (2,12) | 6 (3,13) | 0 | 32.7 (13.2,67.3) | 31.9 (12.8,66.2) | -2.7 (-4.4,-1.1) |
| Greenland | 7 (3,14) | 8 (3,17) | 14 | 14.7 (6,30.2) | 14.3 (5.8,29.6) | -3 (-5.1,-0.6) |
| Guam | 41 (16,85) | 56 (23,115) | 37 | 32.7 (13.2,67.6) | 32.5 (13.2,67.4) | -0.4 (-1.9,1.2) |
| Monaco | 19 (8,40) | 25 (10,52) | 32 | 43.3 (17.8,89.3) | 42.9 (17.7,88.4) | -0.9 (-2.1,0.3) |
| Nauru | 3 (1,6) | 3 (1,7) | 0 | 33.7 (13.7,69.3) | 33.2 (13.6,68.4) | -1.6 (-3.2,0.1) |
| Niue | 1 (0,2) | 1 (0,1) | 0 | 33.1 (13.4,67.8) | 32.6 (13.2,67.1) | -1.4 (-2.9,0.1) |
| Northern Mariana Islands | 13 (5,28) | 16 (7,34) | 23 | 32.9 (13.4,67.3) | 32.6 (13.2,67.4) | -0.9 (-2.4,0.7) |
| Palau | 5 (2,10) | 6 (3,13) | 20 | 33.1 (13.5,68.1) | 32.8 (13.3,67.1) | -0.9 (-2.4,0.6) |
| Puerto Rico | 1681 (695,3498) | 2158 (889,4456) | 28 | 46.4 (19.2,96.4) | 45.8 (18.9,94.9) | -1.4 (-2.5,-0.2) |
| Saint Kitts and Nevis | 19 (8,39) | 28 (12,59) | 47 | 47.6 (19.7,98.5) | 46.6 (19.3,96.8) | -2.1 (-3.3,-0.9) |
| San Marino | 12 (5,25) | 21 (8,43) | 75 | 43.2 (17.8,88.2) | 42.8 (17.6,87.7) | -0.8 (-2,0.3) |
| Tokelau | 0 (0,1) | 0 (0,1) | 0 | 33.2 (13.5,68.6) | 32.6 (13.3,66.9) | -2 (-3.5,-0.4) |
| Tuvalu | 3 (1,6) | 4 (2,8) | 33 | 33.7 (13.8,69.7) | 33.2 (13.5,68.3) | -1.7 (-3.4,-0.1) |
| Virgin Islands | 46 (19,95) | 55 (22,113) | 20 | 46.9 (19.4,96.6) | 46.4 (19.2,95.9) | -1.1 (-2.1,0.1) |
| South Sudan | 5011 (2033,10615) | 7556 (3091,16003) | 51 | 84.8 (35.4,177.9) | 80.1 (33.4,166.5) | -5.5 (-9.3,-2.3) |
| Sudan | 2802 (1128,5771) | 5936 (2401,12032) | 112 | 18.7 (7.6,38.1) | 18.3 (7.5,36.7) | -2.5 (-4.4,-0.5) |

| **Table S6.** Comparative analysis of male-to-female ratios in cases and age-standardized rate (ASR) changes of fungal skin diseases globally from 1990 to 2021 | | | | | | |
| --- | --- | --- | --- | --- | --- | --- |
| **Prevalence** | | | | | | |
| **Year** | **number** | | | **rate** | | |
|  | **Male** | **Female** | **ratio** | **Male** | **Female** | **ratio** |
| 1990 | 191797086.5 | 175784255.3 | 1.09 | 7141.3 | 6638.7 | 1.08 |
| 1991 | 195692038.6 | 179288333.4 | 1.09 | 7172.0 | 6670.1 | 1.08 |
| 1992 | 199582306 | 182748161.4 | 1.09 | 7204.8 | 6701.3 | 1.08 |
| 1993 | 203389409.9 | 186141334.3 | 1.09 | 7237.5 | 6731.4 | 1.08 |
| 1994 | 207001160.9 | 189405146.5 | 1.09 | 7267.6 | 6759.2 | 1.08 |
| 1995 | 210558344.7 | 192590169 | 1.09 | 7297.3 | 6784.6 | 1.08 |
| 1996 | 214282635.3 | 195865605.9 | 1.09 | 7331.5 | 6811.3 | 1.08 |
| 1997 | 218101359.7 | 199192512.5 | 1.09 | 7368.0 | 6838.1 | 1.08 |
| 1998 | 221921555.9 | 202523982.8 | 1.10 | 7403.9 | 6864.0 | 1.08 |
| 1999 | 225749456.1 | 205898179.6 | 1.10 | 7439.2 | 6890.2 | 1.08 |
| 2000 | 229621906.8 | 209375377.7 | 1.10 | 7474.2 | 6917.9 | 1.08 |
| 2001 | 233507734.6 | 212957050.5 | 1.10 | 7507.1 | 6946.4 | 1.08 |
| 2002 | 237380580.7 | 216584415.7 | 1.10 | 7536.8 | 6973.6 | 1.08 |
| 2003 | 241248856.2 | 220265116.3 | 1.10 | 7563.9 | 7000.0 | 1.08 |
| 2004 | 245169295.5 | 224050695.5 | 1.09 | 7590.4 | 7027.1 | 1.08 |
| 2005 | 249197737.4 | 227966149 | 1.09 | 7617.8 | 7055.4 | 1.08 |
| 2006 | 253279053.6 | 231949725.9 | 1.09 | 7643.1 | 7082.7 | 1.08 |
| 2007 | 257363122.4 | 235958553.3 | 1.09 | 7665.0 | 7107.7 | 1.08 |
| 2008 | 261520751.7 | 240035076.1 | 1.09 | 7685.3 | 7131.9 | 1.08 |
| 2009 | 265819431.5 | 244249441 | 1.09 | 7707.8 | 7157.8 | 1.08 |
| 2010 | 270172484.7 | 248561822.6 | 1.09 | 7735.4 | 7187.9 | 1.08 |
| 2011 | 274557253.1 | 252916698 | 1.09 | 7766.4 | 7220.8 | 1.08 |
| 2012 | 279096470.3 | 257348021.3 | 1.08 | 7798.9 | 7254.0 | 1.08 |
| 2013 | 283730680.2 | 261814402.5 | 1.08 | 7831.2 | 7286.5 | 1.07 |
| 2014 | 288378357.6 | 266295596.6 | 1.08 | 7862.6 | 7319.0 | 1.07 |
| 2015 | 292972074.7 | 270744756.6 | 1.08 | 7890.9 | 7349.9 | 1.07 |
| 2016 | 297466585.1 | 275197497.3 | 1.08 | 7915.6 | 7380.7 | 1.07 |
| 2017 | 301852643 | 279658282.9 | 1.08 | 7938.0 | 7412.2 | 1.07 |
| 2018 | 306168742.7 | 284072367.6 | 1.08 | 7960.6 | 7444.2 | 1.07 |
| 2019 | 310526374.5 | 288477415.8 | 1.08 | 7987.3 | 7478.2 | 1.07 |
| 2020 | 314371057.8 | 292349028.7 | 1.08 | 8007.6 | 7503.0 | 1.07 |
| 2021 | 319142132 | 297390154.5 | 1.07 | 8060.4 | 7563.4 | 1.07 |
| **Incidence** | | | | | | |
| **Year** | **number** | | | **rate** | | |
|  | **Male** | **Female** | **ratio** | **Male** | **Female** | **ratio** |
| 1990 | 524897891.1 | 504827217.6 | 1.04 | 21472.1 | 20263.5 | 1.06 |
| 1991 | 534668432 | 514847038.2 | 1.04 | 21486.6 | 20307.5 | 1.06 |
| 1992 | 544444831 | 524714178.3 | 1.04 | 21505.4 | 20348.8 | 1.06 |
| 1993 | 554102244.1 | 534379954.6 | 1.04 | 21528.5 | 20387.1 | 1.06 |
| 1994 | 563502588.9 | 543751264.6 | 1.04 | 21554.3 | 20420.9 | 1.06 |
| 1995 | 572940888.6 | 552905254.3 | 1.04 | 21586.1 | 20450.1 | 1.06 |
| 1996 | 582842802 | 562145445.2 | 1.04 | 21629.7 | 20478.2 | 1.06 |
| 1997 | 593067025.9 | 571443249.4 | 1.04 | 21680.1 | 20502.0 | 1.06 |
| 1998 | 603392870.8 | 580771857.7 | 1.04 | 21732.3 | 20522.1 | 1.06 |
| 1999 | 613776451.1 | 590275839.8 | 1.04 | 21784.5 | 20542.6 | 1.06 |
| 2000 | 624212601.5 | 600121758.4 | 1.04 | 21835.2 | 20568.2 | 1.06 |
| 2001 | 634509938.3 | 610260129.1 | 1.04 | 21874.8 | 20593.8 | 1.06 |
| 2002 | 644616667.7 | 620483187.4 | 1.04 | 21899.9 | 20613.6 | 1.06 |
| 2003 | 654678362.7 | 630859430.1 | 1.04 | 21917.7 | 20630.6 | 1.06 |
| 2004 | 665006177.5 | 641605419 | 1.04 | 21936.6 | 20648.8 | 1.06 |
| 2005 | 675936698.8 | 652878310 | 1.04 | 21965.6 | 20673.6 | 1.06 |
| 2006 | 687538650.5 | 664692631.8 | 1.03 | 21999.1 | 20701.6 | 1.06 |
| 2007 | 699562241.9 | 676905203.2 | 1.03 | 22031.5 | 20730.2 | 1.06 |
| 2008 | 711925529.1 | 689411155.6 | 1.03 | 22063.0 | 20758.9 | 1.06 |
| 2009 | 724532294.3 | 702192278.7 | 1.03 | 22093.3 | 20786.3 | 1.06 |
| 2010 | 736877398.5 | 714909250.7 | 1.03 | 22122.1 | 20811.1 | 1.06 |
| 2011 | 748917310.3 | 727489506.3 | 1.03 | 22143.6 | 20831.0 | 1.06 |
| 2012 | 761202274.5 | 740260238.9 | 1.03 | 22160.2 | 20848.5 | 1.06 |
| 2013 | 773613126.8 | 753087613 | 1.03 | 22173.6 | 20864.1 | 1.06 |
| 2014 | 786046306.8 | 765967299.8 | 1.03 | 22183.7 | 20877.5 | 1.06 |
| 2015 | 798443460.6 | 778808093.9 | 1.03 | 22191.6 | 20890.1 | 1.06 |
| 2016 | 810715264.1 | 791716133.6 | 1.02 | 22187.4 | 20896.1 | 1.06 |
| 2017 | 822767311.6 | 804663781 | 1.02 | 22173.1 | 20898.3 | 1.06 |
| 2018 | 834782042.2 | 817541542.9 | 1.02 | 22161.0 | 20901.3 | 1.06 |
| 2019 | 847205349.9 | 830552321.4 | 1.02 | 22163.8 | 20909.8 | 1.06 |
| 2020 | 859640862.4 | 841802524.7 | 1.02 | 22202.1 | 20894.2 | 1.06 |
| 2021 | 870653138.3 | 858570870.4 | 1.01 | 22238.6 | 21066.0 | 1.06 |
| **DALYs** | | | | | | |
| **Year** | **number** | | | **rate** | | |
|  | **Male** | **Female** | **ratio** | **Male** | **Female** | **ratio** |
| 1990 | 1078269.719 | 978161.3289 | 1.10 | 42.8 | 38.6 | 1.11 |
| 1991 | 1100203.536 | 997705.0661 | 1.10 | 42.9 | 38.7 | 1.11 |
| 1992 | 1122094.474 | 1016914.329 | 1.10 | 43.1 | 38.8 | 1.11 |
| 1993 | 1143429.682 | 1035878.689 | 1.10 | 43.2 | 38.9 | 1.11 |
| 1994 | 1163661.575 | 1053955.55 | 1.10 | 43.3 | 39.0 | 1.11 |
| 1995 | 1183552.786 | 1071544.814 | 1.10 | 43.4 | 39.1 | 1.11 |
| 1996 | 1204407.212 | 1089587.81 | 1.11 | 43.5 | 39.2 | 1.11 |
| 1997 | 1225785.195 | 1107922.944 | 1.11 | 43.7 | 39.3 | 1.11 |
| 1998 | 1247056.582 | 1126244.107 | 1.11 | 43.8 | 39.3 | 1.11 |
| 1999 | 1268460.819 | 1144737.777 | 1.11 | 44.0 | 39.4 | 1.12 |
| 2000 | 1290082.602 | 1163781.885 | 1.11 | 44.1 | 39.5 | 1.12 |
| 2001 | 1311785.938 | 1183451.369 | 1.11 | 44.3 | 39.6 | 1.12 |
| 2002 | 1333418.984 | 1203457.511 | 1.11 | 44.4 | 39.7 | 1.12 |
| 2003 | 1355050.901 | 1223747.142 | 1.11 | 44.5 | 39.8 | 1.12 |
| 2004 | 1377001.662 | 1244646.614 | 1.11 | 44.7 | 39.9 | 1.12 |
| 2005 | 1399486.973 | 1266153.983 | 1.11 | 44.8 | 40.0 | 1.12 |
| 2006 | 1422283.708 | 1288065.596 | 1.10 | 44.9 | 40.1 | 1.12 |
| 2007 | 1445128.557 | 1310151.311 | 1.10 | 45.0 | 40.1 | 1.12 |
| 2008 | 1468411.199 | 1332598.562 | 1.10 | 45.0 | 40.2 | 1.12 |
| 2009 | 1492458.022 | 1355742.968 | 1.10 | 45.1 | 40.2 | 1.12 |
| 2010 | 1516727.677 | 1379446.272 | 1.10 | 45.2 | 40.3 | 1.12 |
| 2011 | 1541109.381 | 1403220.844 | 1.10 | 45.2 | 40.4 | 1.12 |
| 2012 | 1566436.69 | 1427432.096 | 1.10 | 45.3 | 40.4 | 1.12 |
| 2013 | 1592188.349 | 1451802.366 | 1.10 | 45.4 | 40.5 | 1.12 |
| 2014 | 1618022.972 | 1476350.849 | 1.10 | 45.5 | 40.6 | 1.12 |
| 2015 | 1643507.362 | 1500536.625 | 1.10 | 45.6 | 40.6 | 1.12 |
| 2016 | 1668412.256 | 1524803.094 | 1.09 | 45.6 | 40.7 | 1.12 |
| 2017 | 1692577.868 | 1548901.183 | 1.09 | 45.6 | 40.7 | 1.12 |
| 2018 | 1716295.272 | 1572743.404 | 1.09 | 45.6 | 40.7 | 1.12 |
| 2019 | 1740189.169 | 1596482.868 | 1.09 | 45.6 | 40.7 | 1.12 |
| 2020 | 1760556.195 | 1616400.457 | 1.09 | 45.6 | 40.7 | 1.12 |
| 2021 | 1786507.17 | 1642989.668 | 1.09 | 45.8 | 40.9 | 1.12 |

| **Table S7.** Comparative analysis of male-to-female ratios in cases and age-standardized rate (ASR) changes of fungal skin diseases across global and 5 SDI territories in 2021 | | | | | | |
| --- | --- | --- | --- | --- | --- | --- |
| **Location** | **number** | | | **rate** | | |
|  | **prevalence** | **incidence** | **DALYs** | **prevalence** | **incidence** | **DALYs** |
| Global | 1.07 | 1.01 | 1.09 | 1.11 | 1.06 | 1.12 |
| Low SDI | 1.25 | 1.11 | 1.26 | 1.2 | 1.08 | 1.21 |
| Low-middle SDI | 1.08 | 1.04 | 1.09 | 1.07 | 1.05 | 1.08 |
| Middle SDI | 1.06 | 1.02 | 1.07 | 1.08 | 1.05 | 1.09 |
| High-middle SDI | 0.95 | 0.93 | 0.96 | 1.06 | 1.04 | 1.07 |
| High SDI | 0.94 | 0.91 | 0.95 | 1.1 | 1.07 | 1.1 |
