# Supplementary figures and images for: A systematic analysis of the global, regional, and national burden of fungal skin diseases from 1990 to 2021
Source: Front Epidemiol. 2024 Dec 16;4:1489148. doi: 10.3389/fepid.2024.1489148 (PMC11686433; doi:10.3389/fepid.2024.1489148)

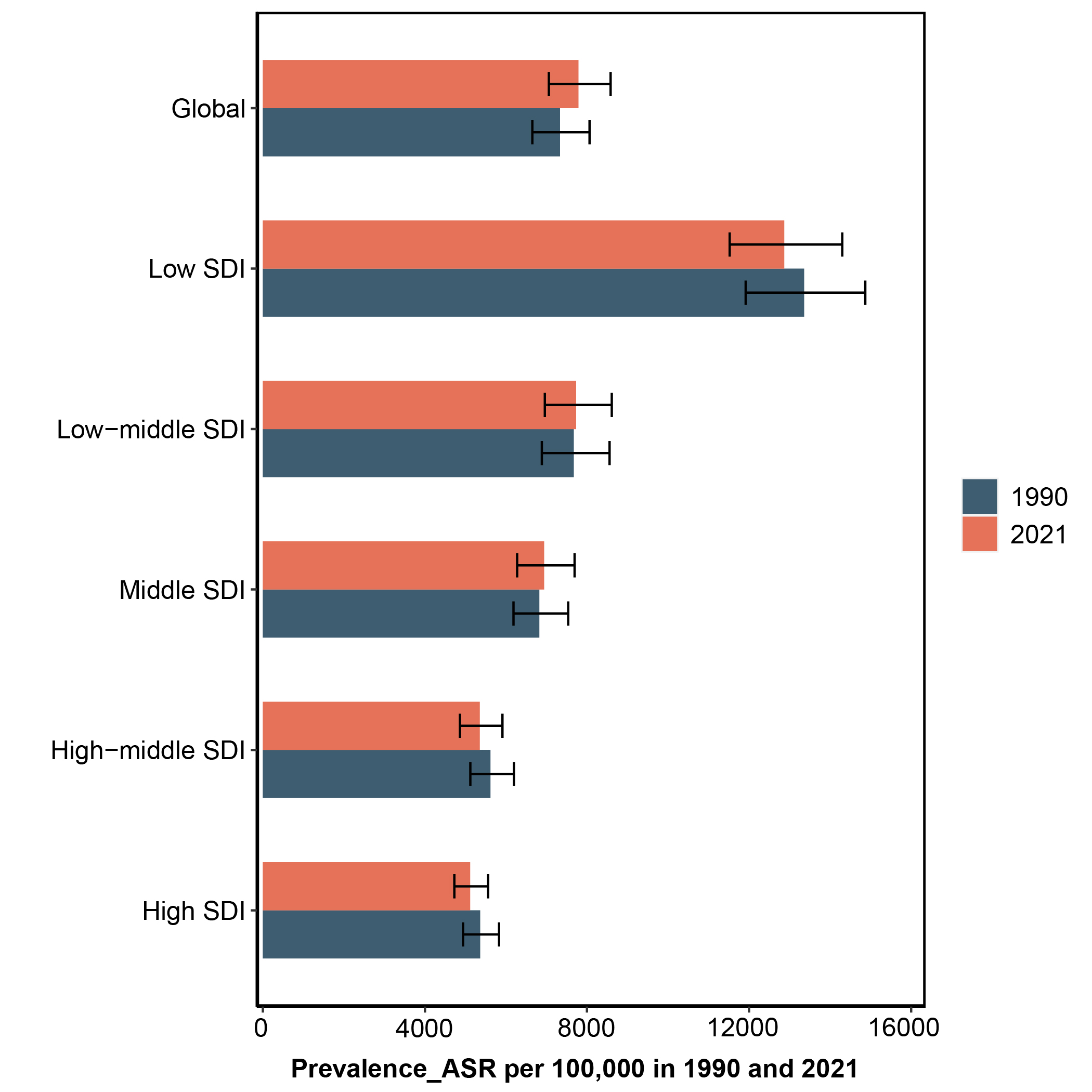

Supplement: Supplementary file 1 [file Datasheet1.zip › FSD supplementary material-20241205-v2/Figure S1-S33/Figure S1.jpg]

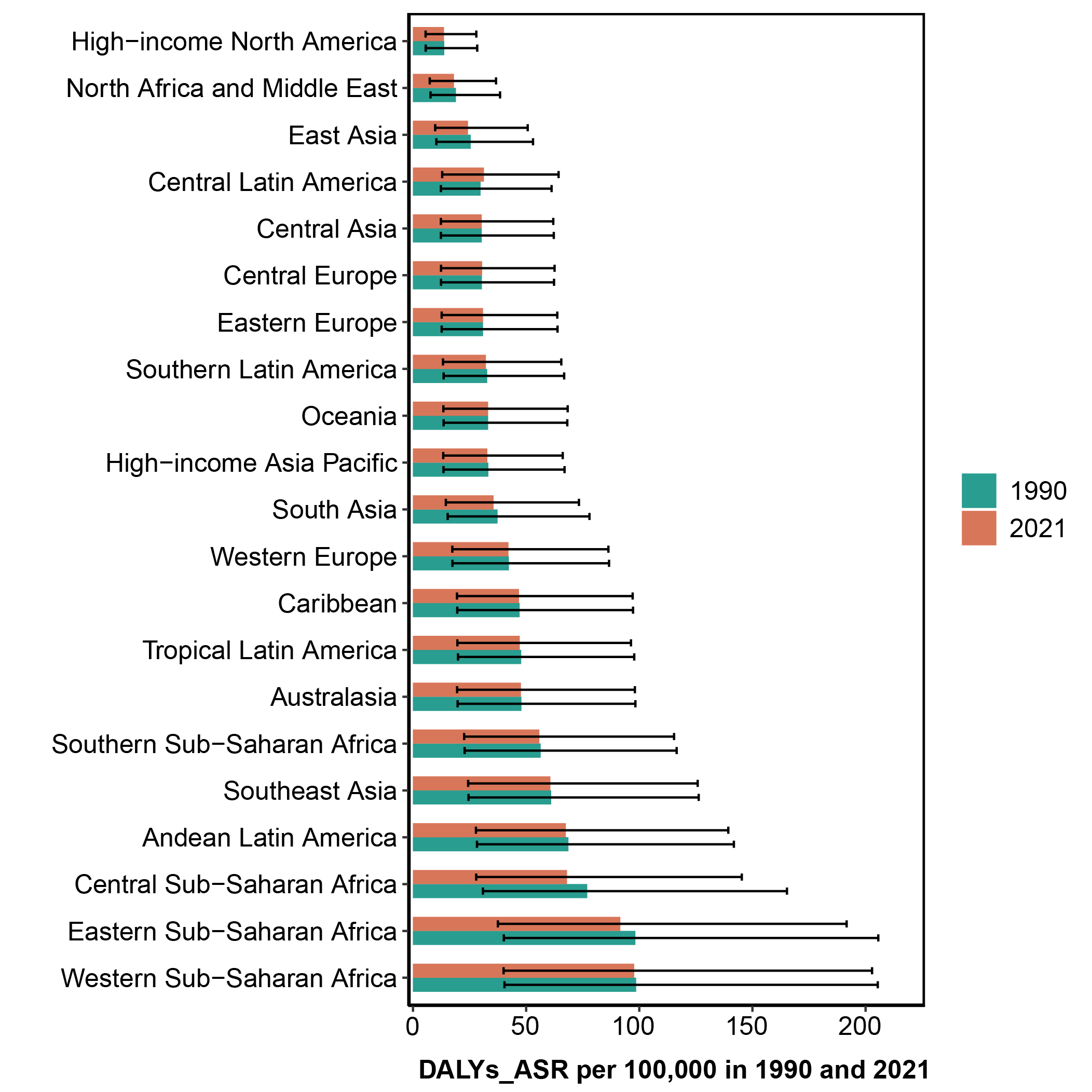

Supplement: Supplementary file 1 [file Datasheet1.zip › FSD supplementary material-20241205-v2/Figure S1-S33/Figure S10.jpg]

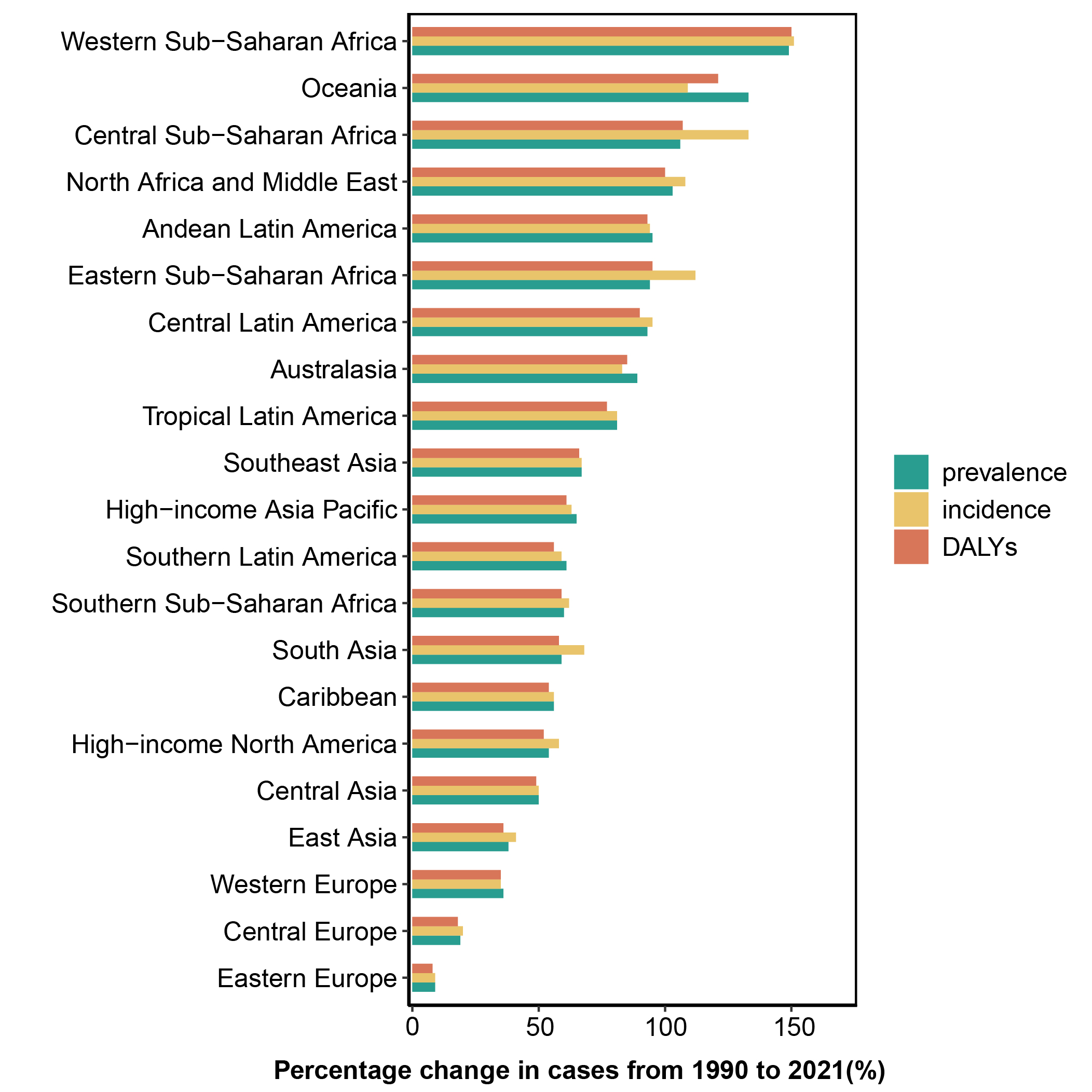

Supplement: Supplementary file 1 [file Datasheet1.zip › FSD supplementary material-20241205-v2/Figure S1-S33/Figure S11.jpg]

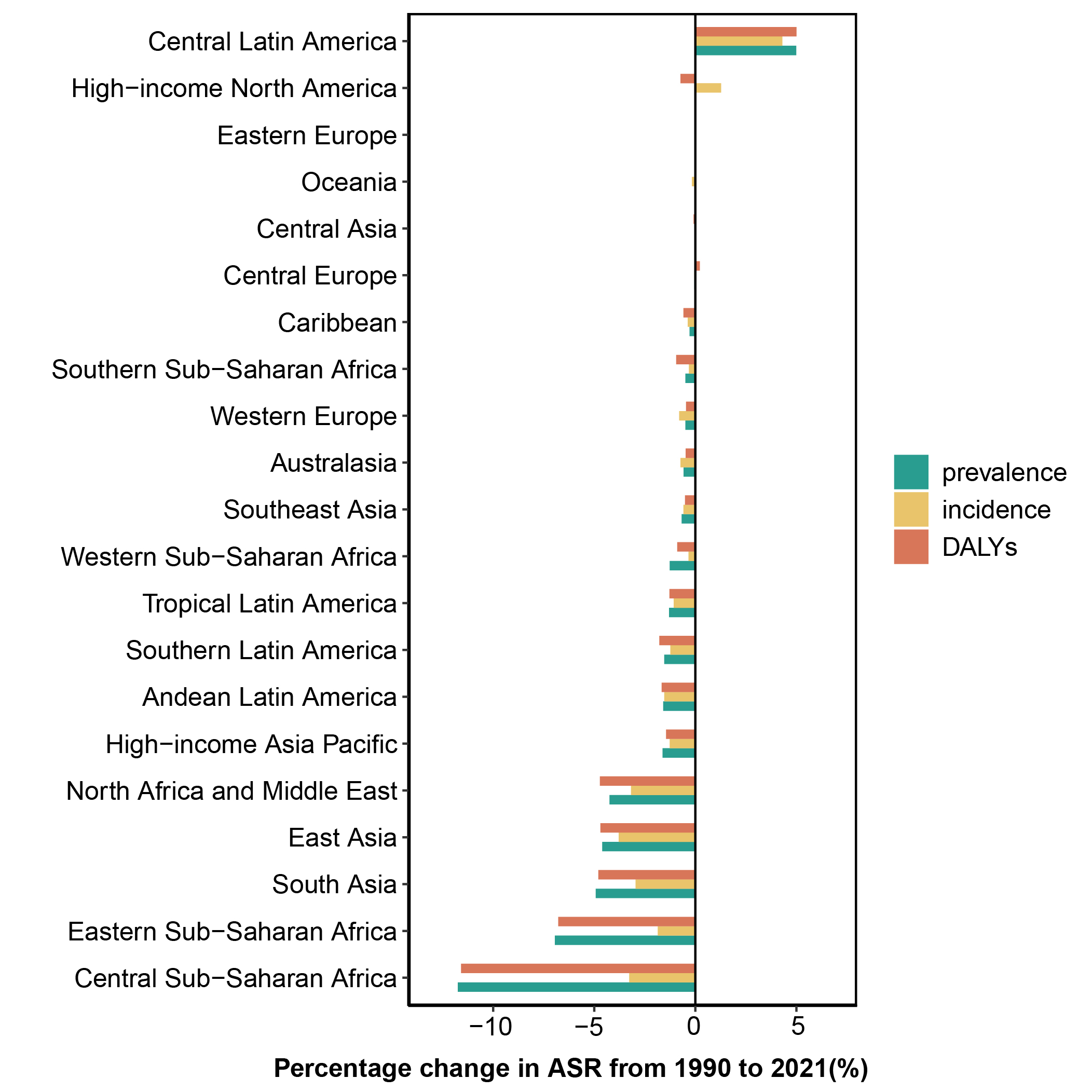

Supplement: Supplementary file 1 [file Datasheet1.zip › FSD supplementary material-20241205-v2/Figure S1-S33/Figure S12.jpg]

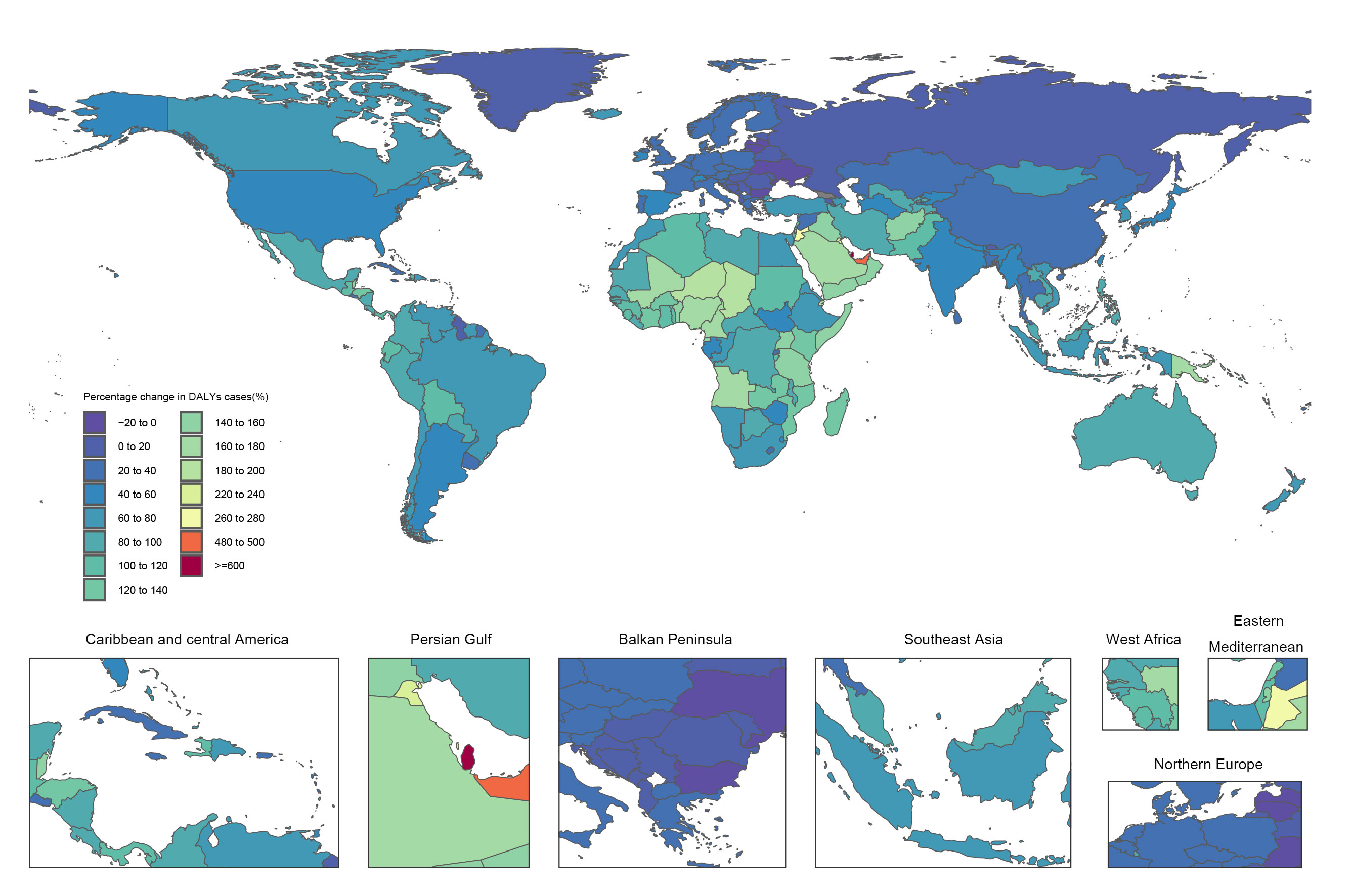

Supplement: Supplementary file 1 [file Datasheet1.zip › FSD supplementary material-20241205-v2/Figure S1-S33/Figure S14.jpg]

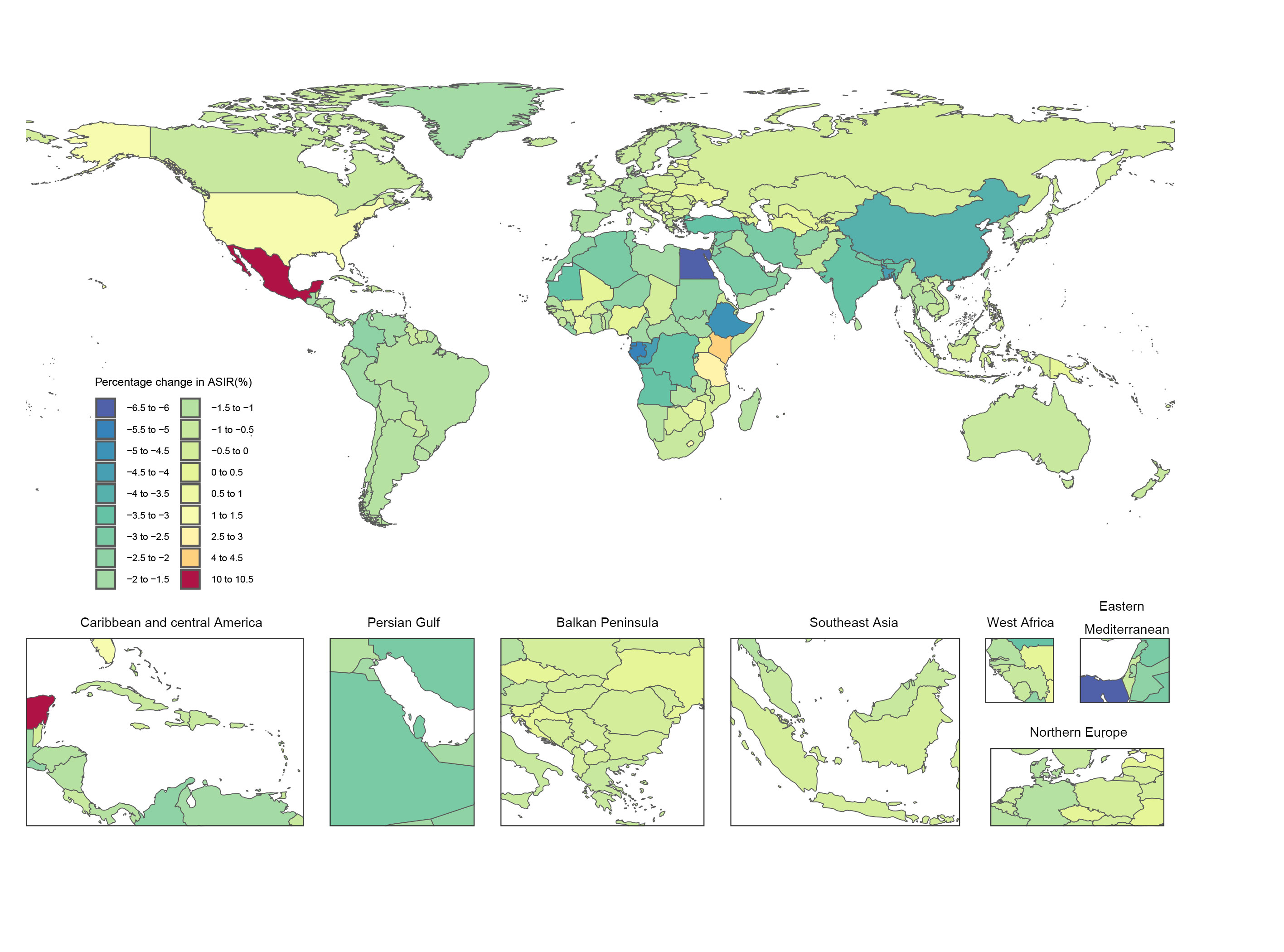

Supplement: Supplementary file 1 [file Datasheet1.zip › FSD supplementary material-20241205-v2/Figure S1-S33/Figure S15.jpg]

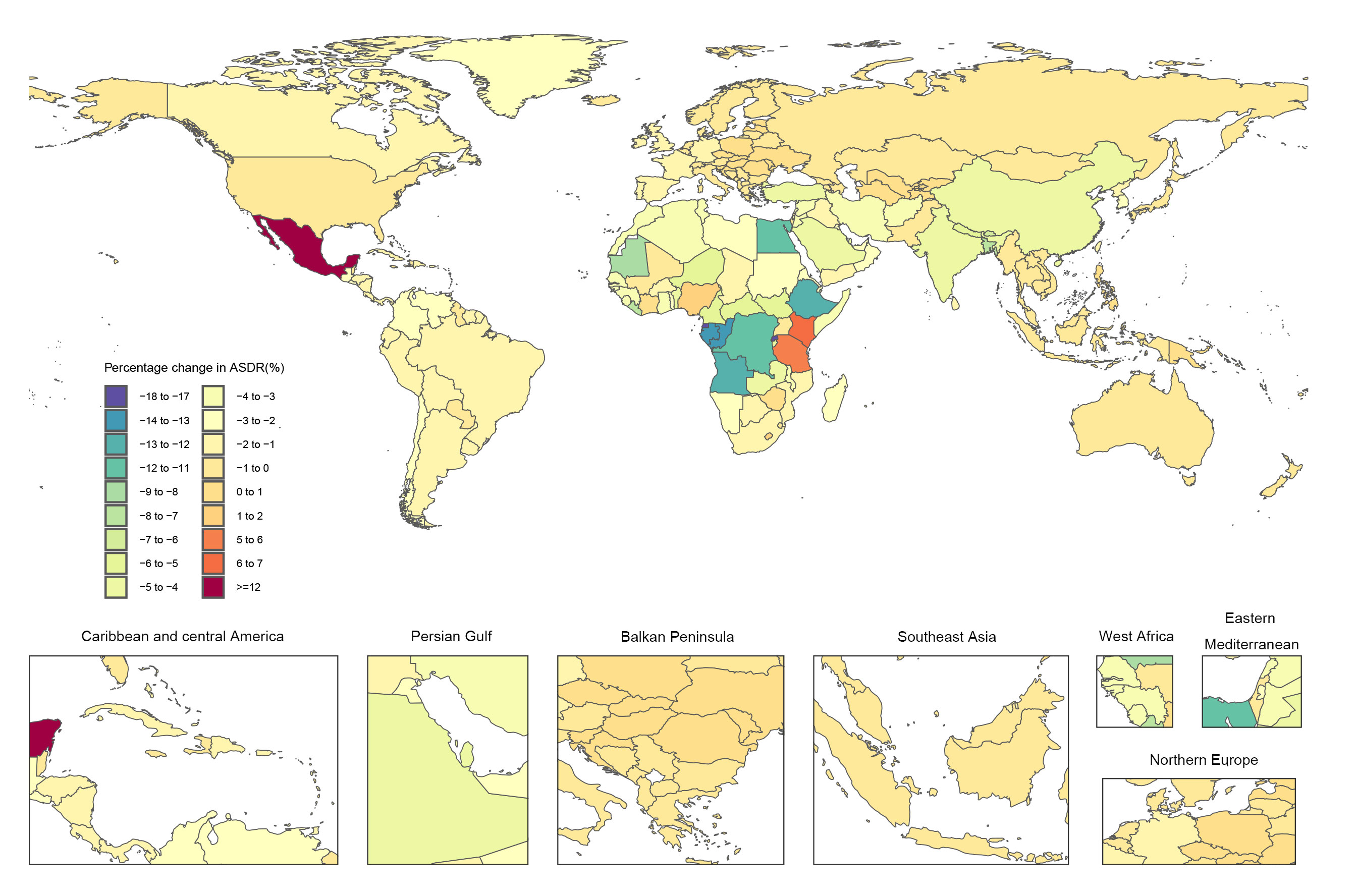

Supplement: Supplementary file 1 [file Datasheet1.zip › FSD supplementary material-20241205-v2/Figure S1-S33/Figure S16.jpg]

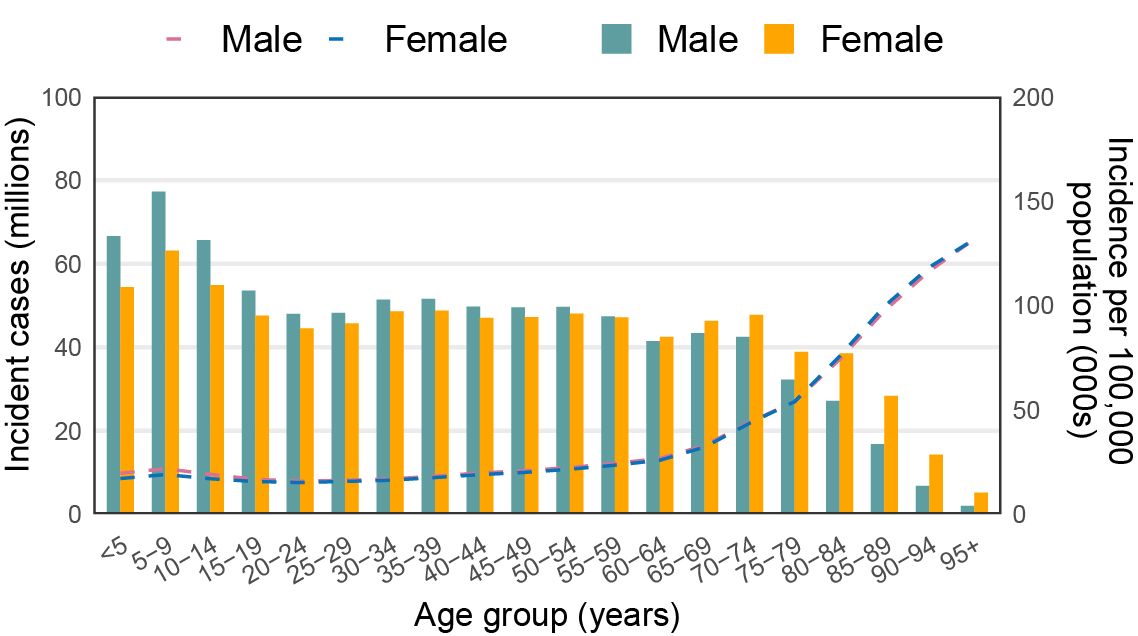

Supplement: Supplementary file 1 [file Datasheet1.zip › FSD supplementary material-20241205-v2/Figure S1-S33/Figure S17.jpg]

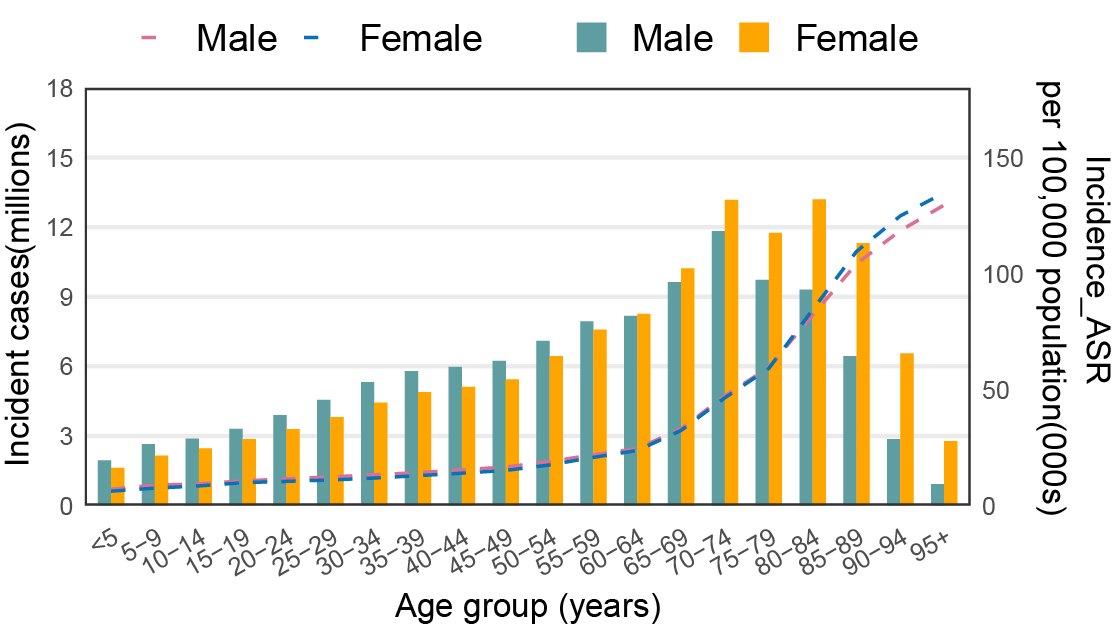

Supplement: Supplementary file 1 [file Datasheet1.zip › FSD supplementary material-20241205-v2/Figure S1-S33/Figure S18.jpg]

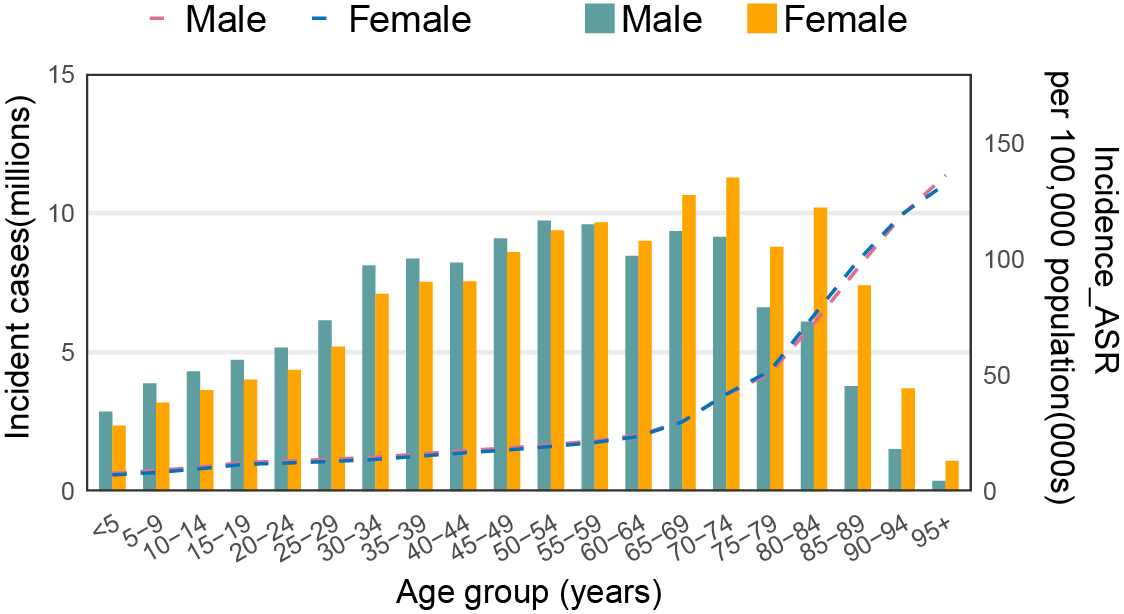

Supplement: Supplementary file 1 [file Datasheet1.zip › FSD supplementary material-20241205-v2/Figure S1-S33/Figure S19.jpg]

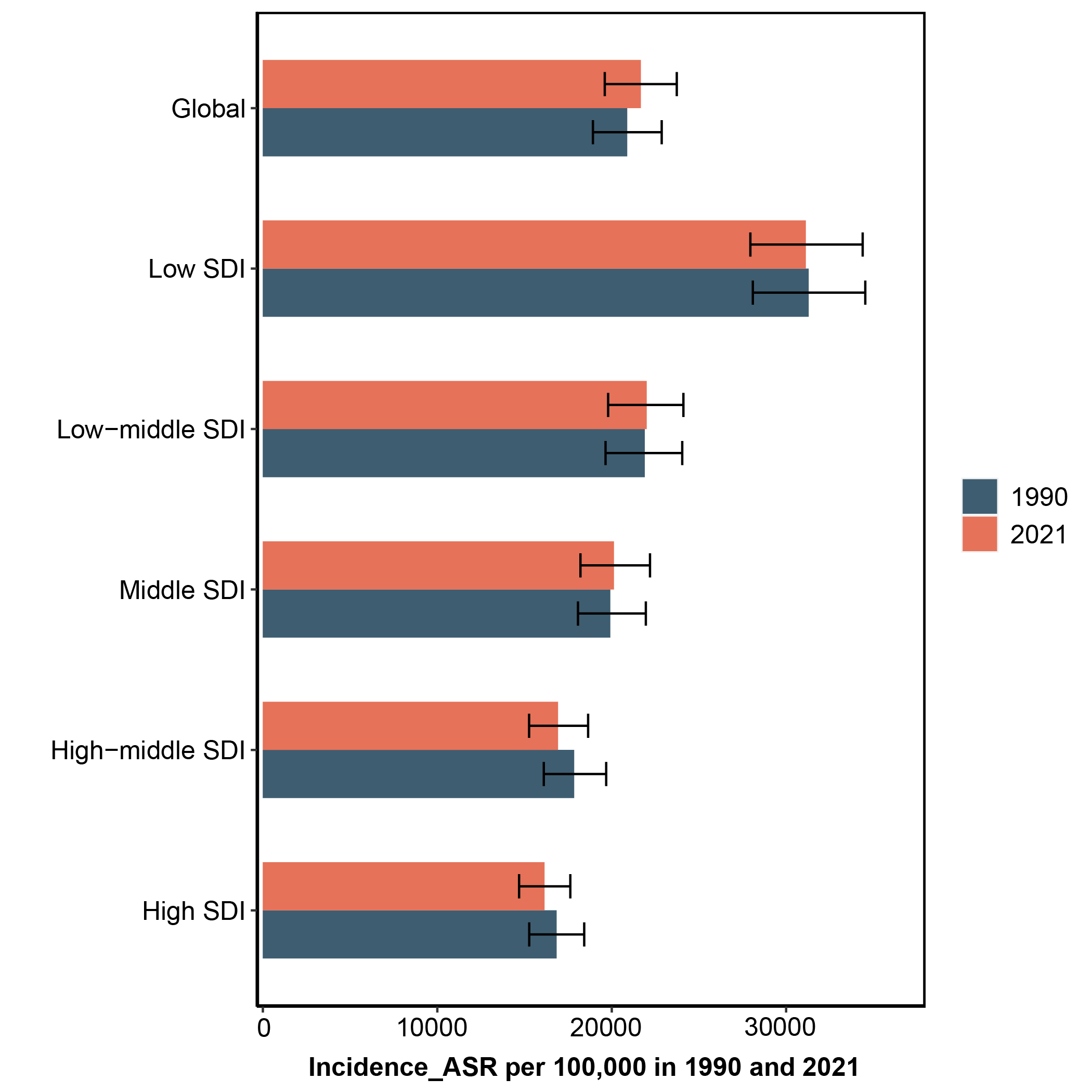

Supplement: Supplementary file 1 [file Datasheet1.zip › FSD supplementary material-20241205-v2/Figure S1-S33/Figure S2.jpg]

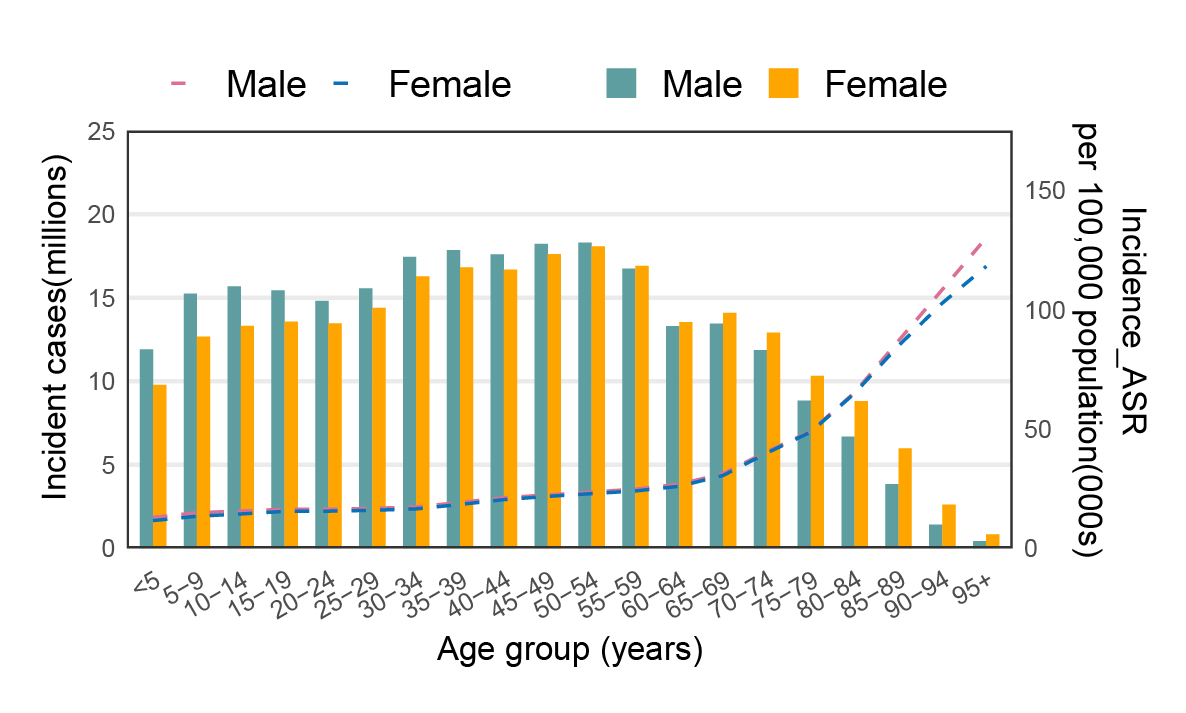

Supplement: Supplementary file 1 [file Datasheet1.zip › FSD supplementary material-20241205-v2/Figure S1-S33/Figure S20.jpg]

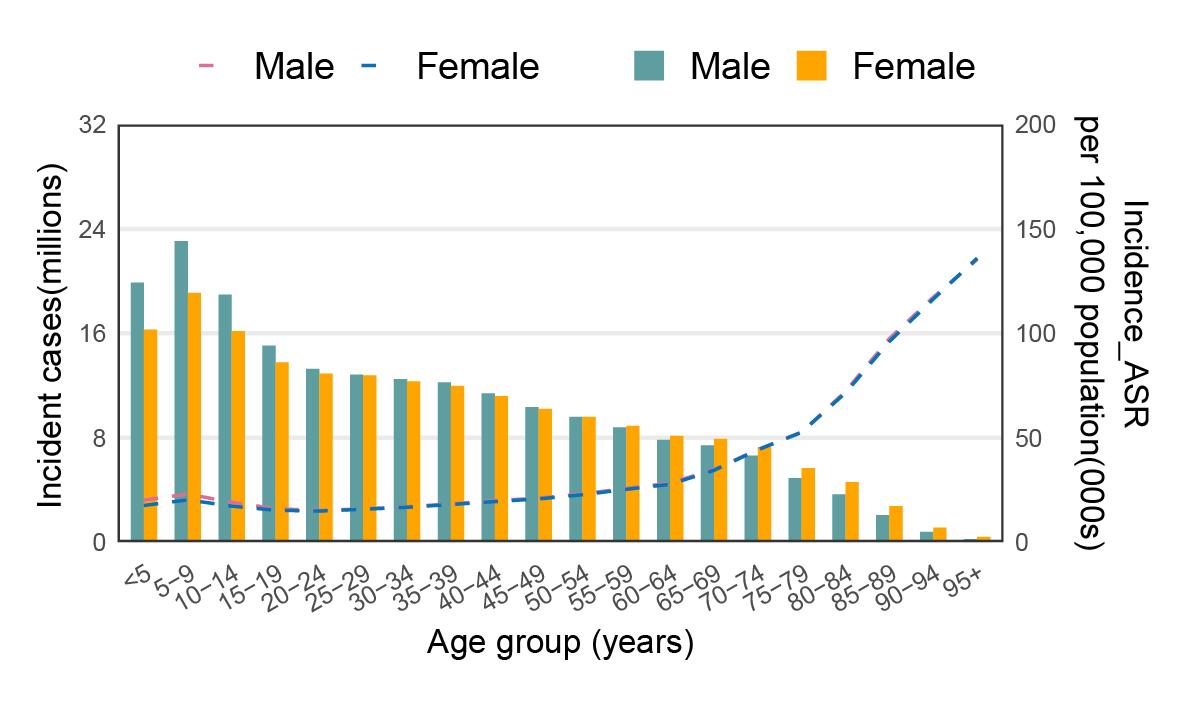

Supplement: Supplementary file 1 [file Datasheet1.zip › FSD supplementary material-20241205-v2/Figure S1-S33/Figure S21.jpg]

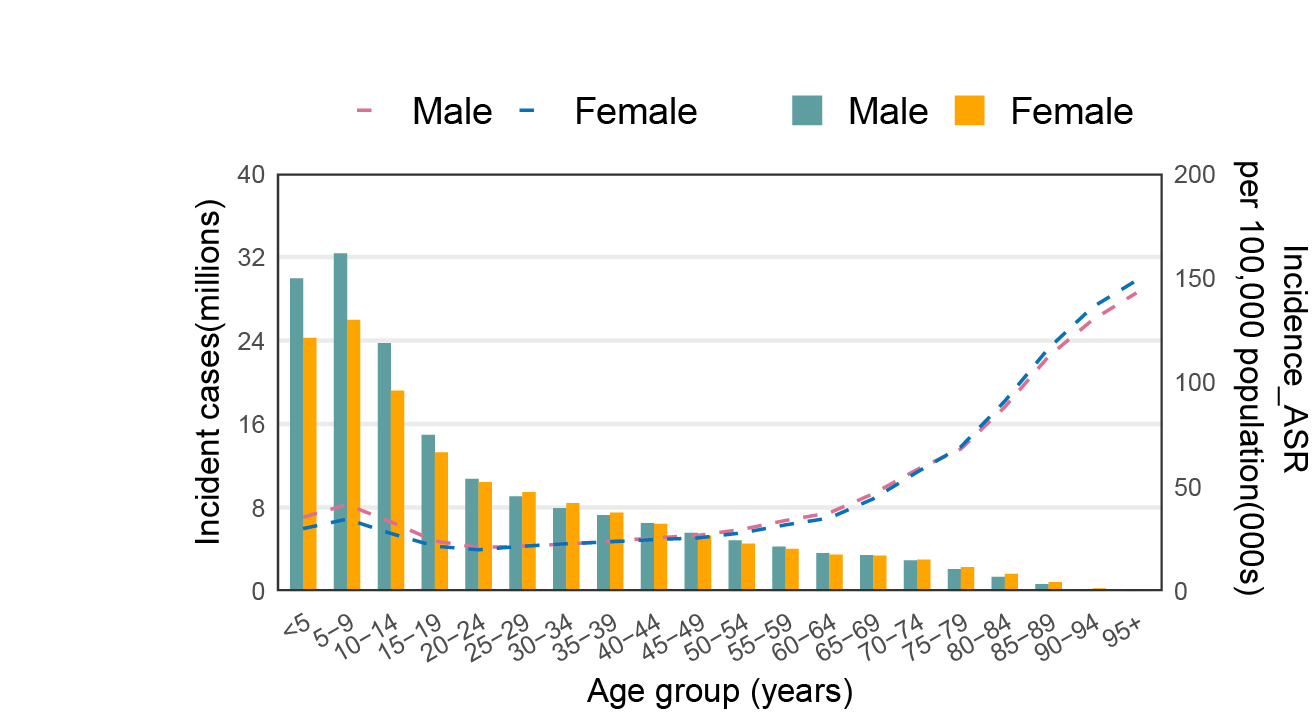

Supplement: Supplementary file 1 [file Datasheet1.zip › FSD supplementary material-20241205-v2/Figure S1-S33/Figure S22.jpg]

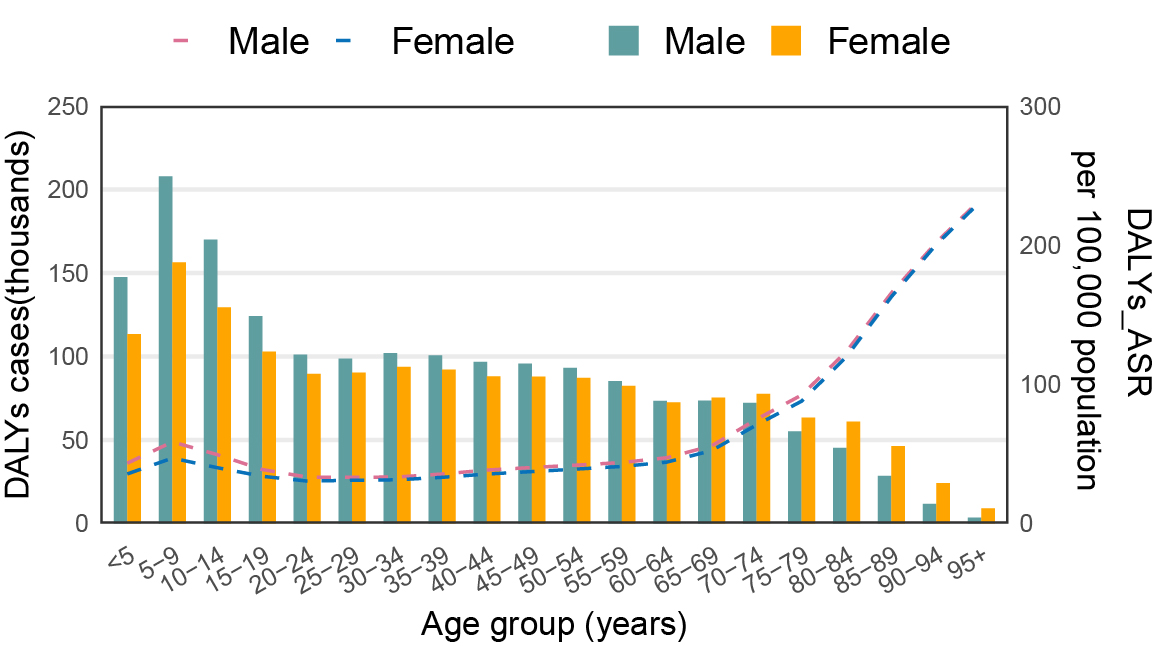

Supplement: Supplementary file 1 [file Datasheet1.zip › FSD supplementary material-20241205-v2/Figure S1-S33/Figure S23l.jpg]

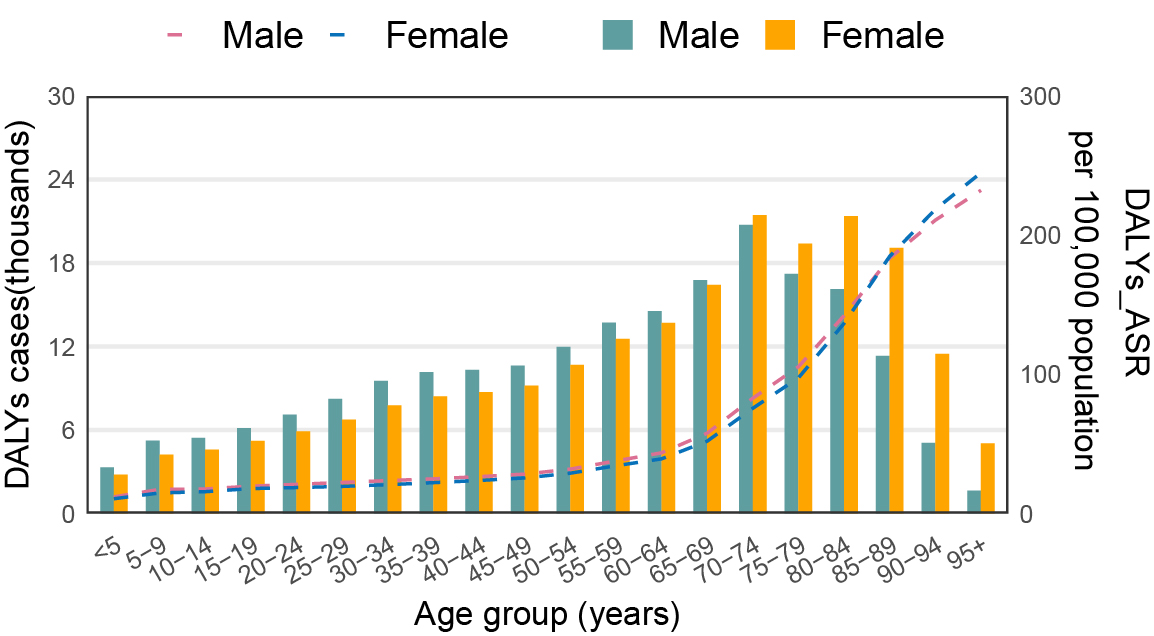

Supplement: Supplementary file 1 [file Datasheet1.zip › FSD supplementary material-20241205-v2/Figure S1-S33/Figure S24.jpg]

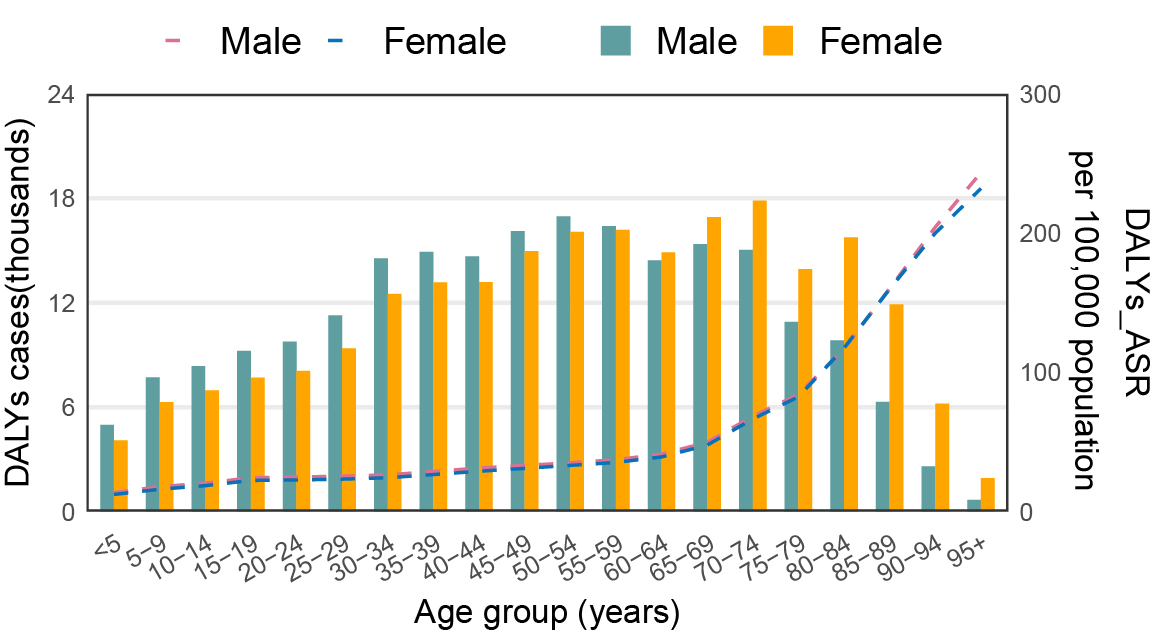

Supplement: Supplementary file 1 [file Datasheet1.zip › FSD supplementary material-20241205-v2/Figure S1-S33/Figure S25.jpg]

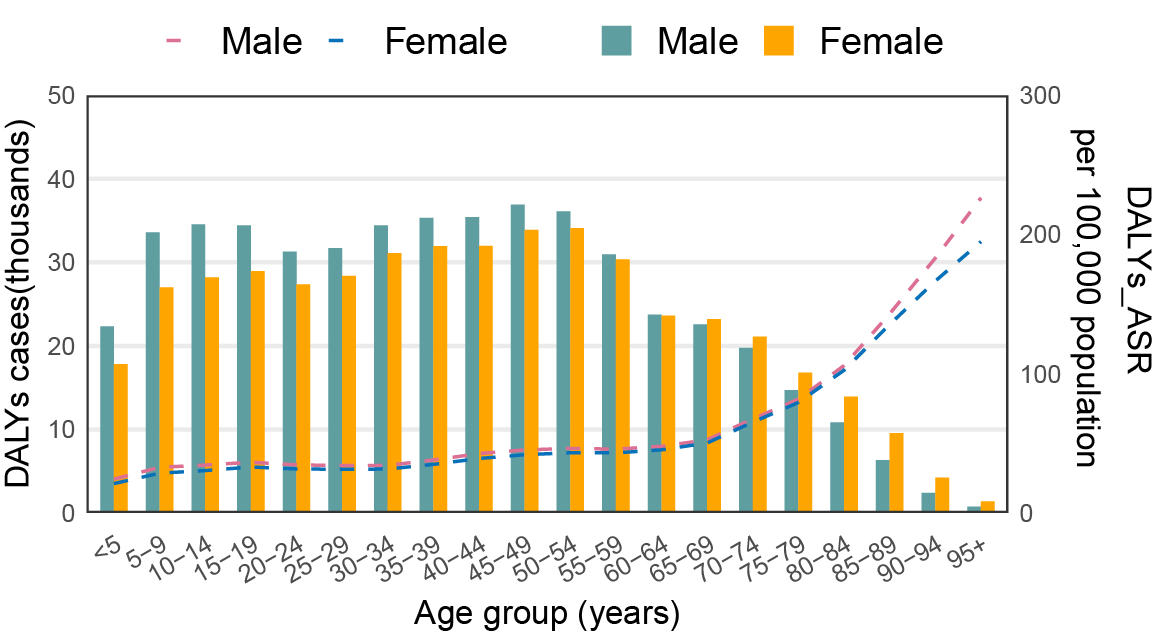

Supplement: Supplementary file 1 [file Datasheet1.zip › FSD supplementary material-20241205-v2/Figure S1-S33/Figure S26.jpg]

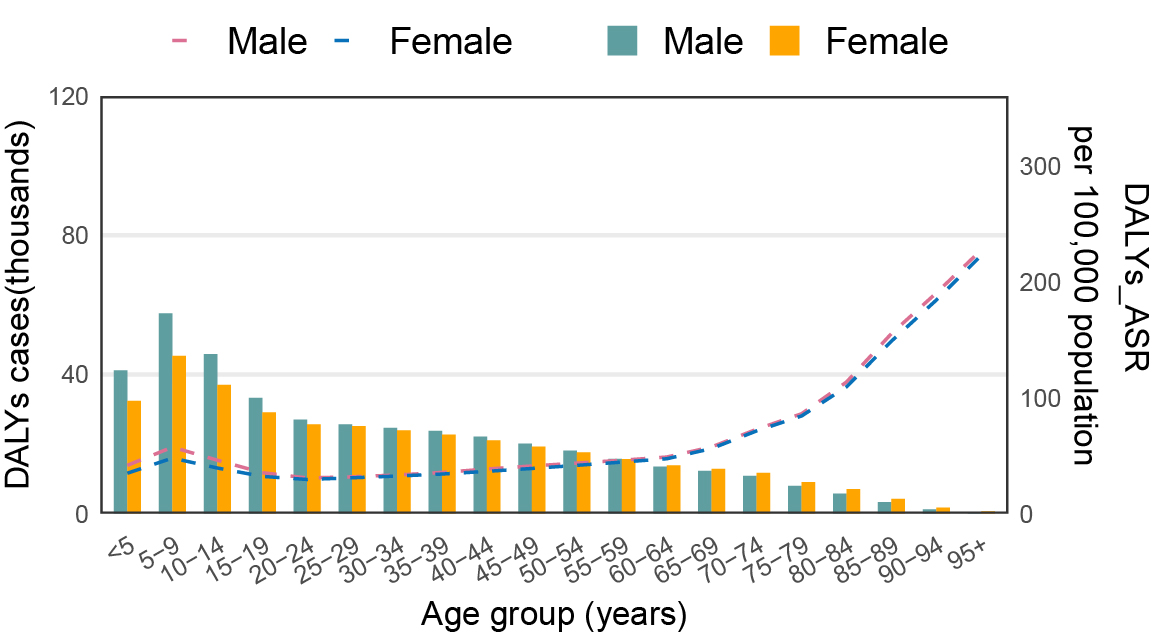

Supplement: Supplementary file 1 [file Datasheet1.zip › FSD supplementary material-20241205-v2/Figure S1-S33/Figure S27.jpg]

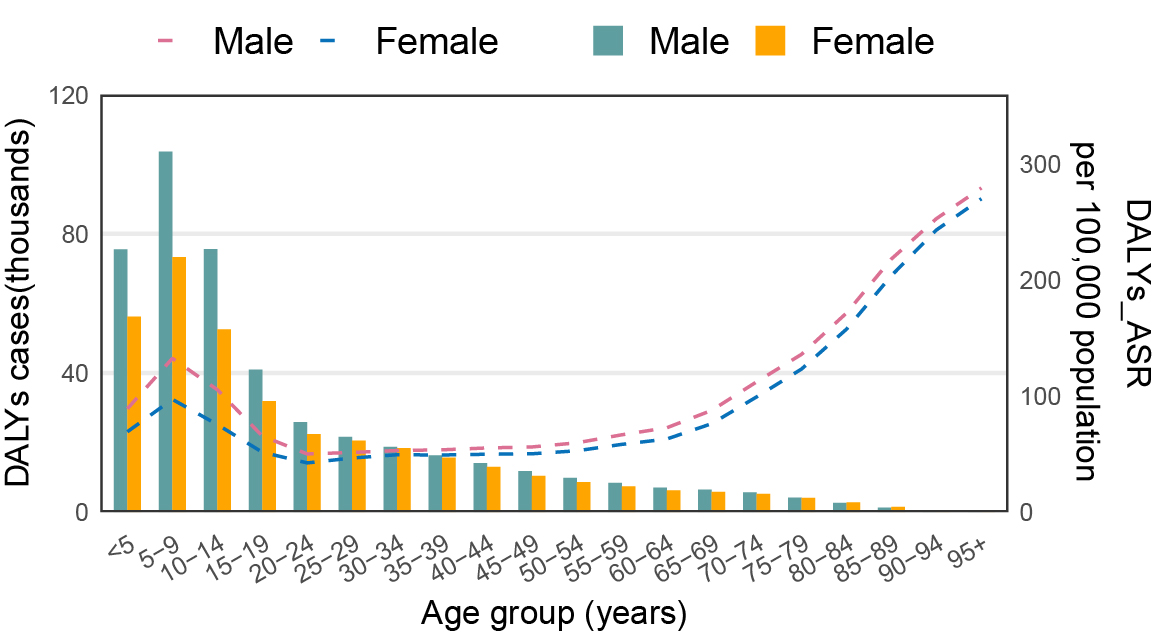

Supplement: Supplementary file 1 [file Datasheet1.zip › FSD supplementary material-20241205-v2/Figure S1-S33/Figure S28.jpg]

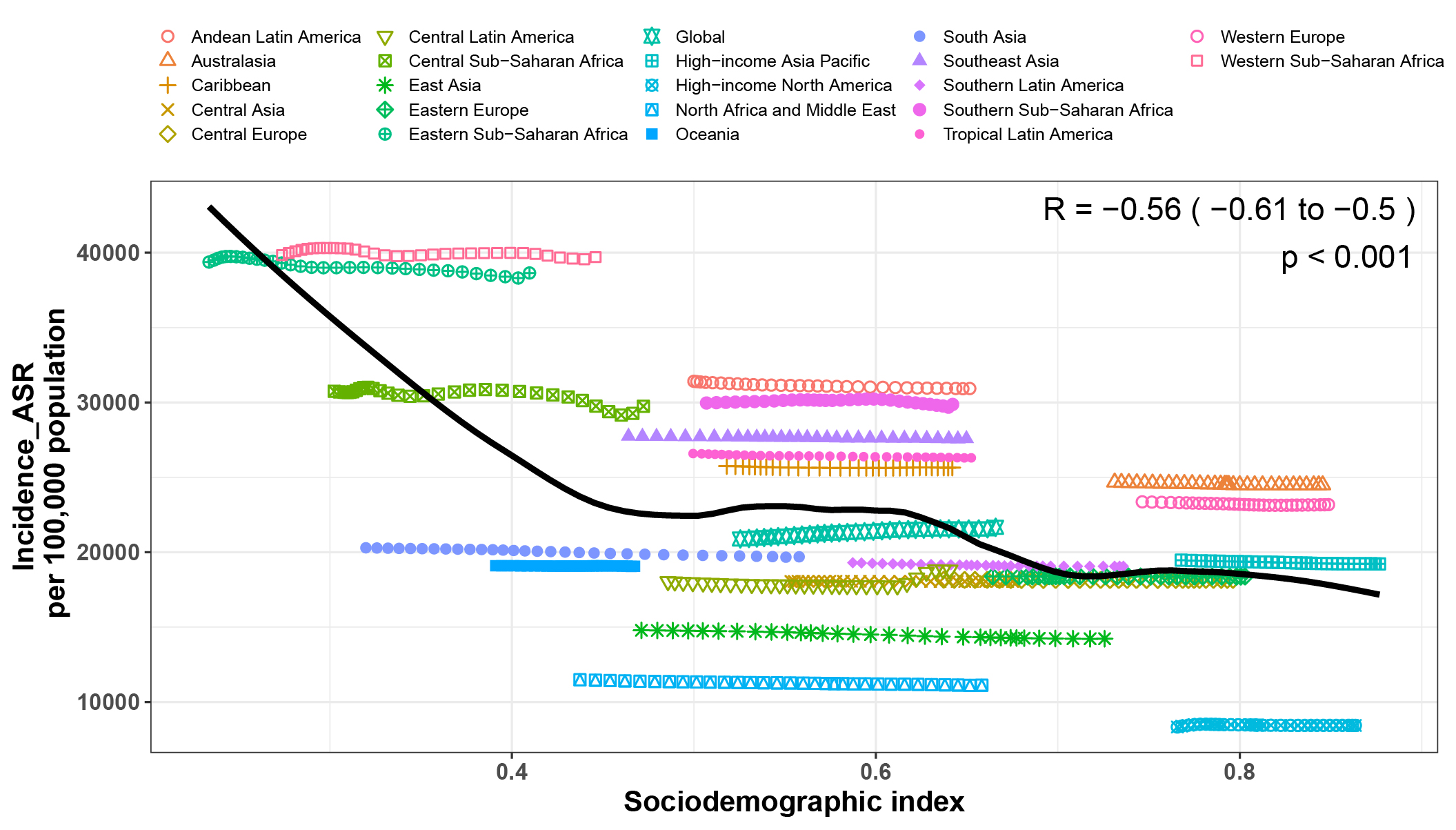

Supplement: Supplementary file 1 [file Datasheet1.zip › FSD supplementary material-20241205-v2/Figure S1-S33/Figure S29.jpg]

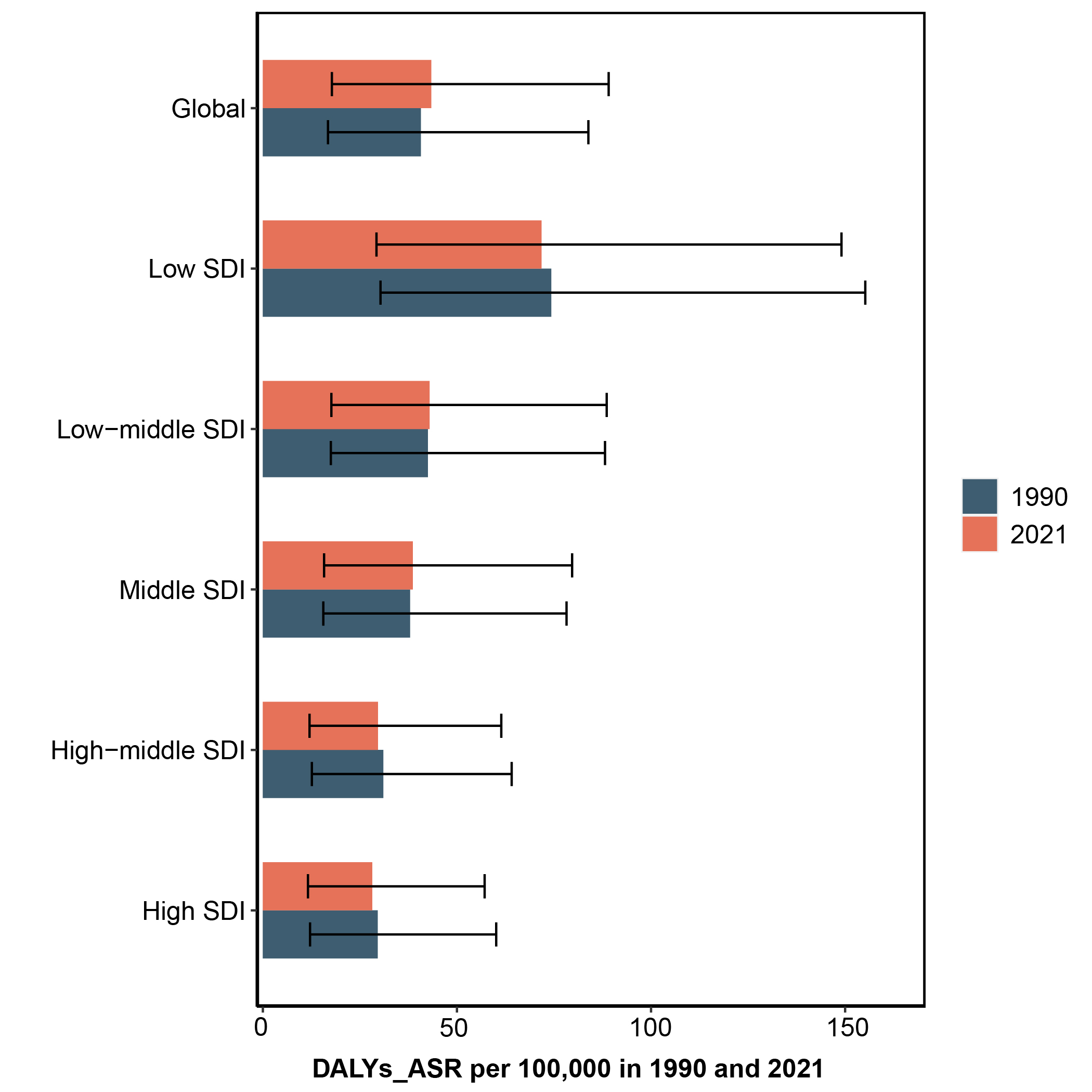

Supplement: Supplementary file 1 [file Datasheet1.zip › FSD supplementary material-20241205-v2/Figure S1-S33/Figure S3.jpg]

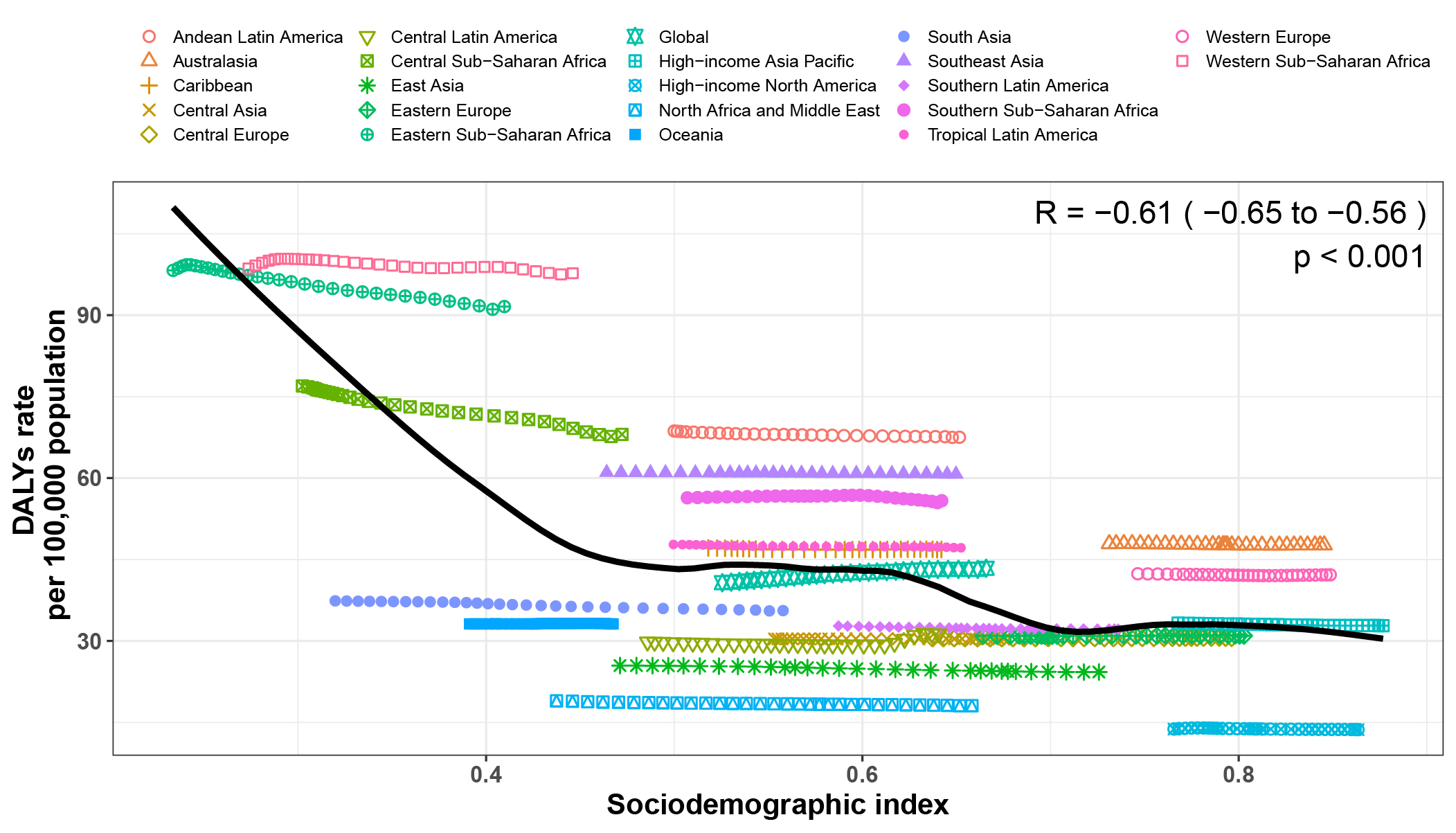

Supplement: Supplementary file 1 [file Datasheet1.zip › FSD supplementary material-20241205-v2/Figure S1-S33/Figure S30.jpg]

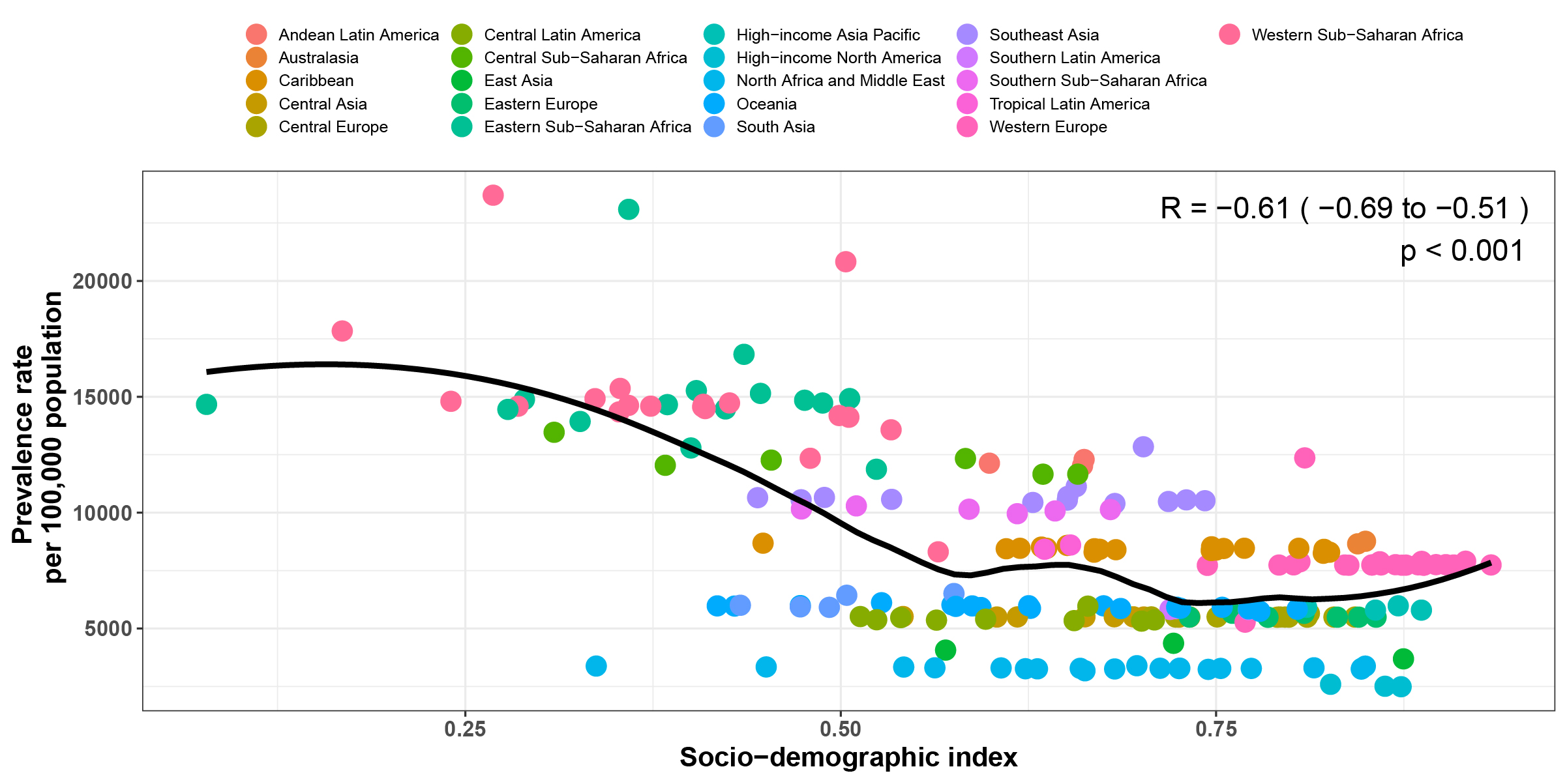

Supplement: Supplementary file 1 [file Datasheet1.zip › FSD supplementary material-20241205-v2/Figure S1-S33/Figure S31.jpg]

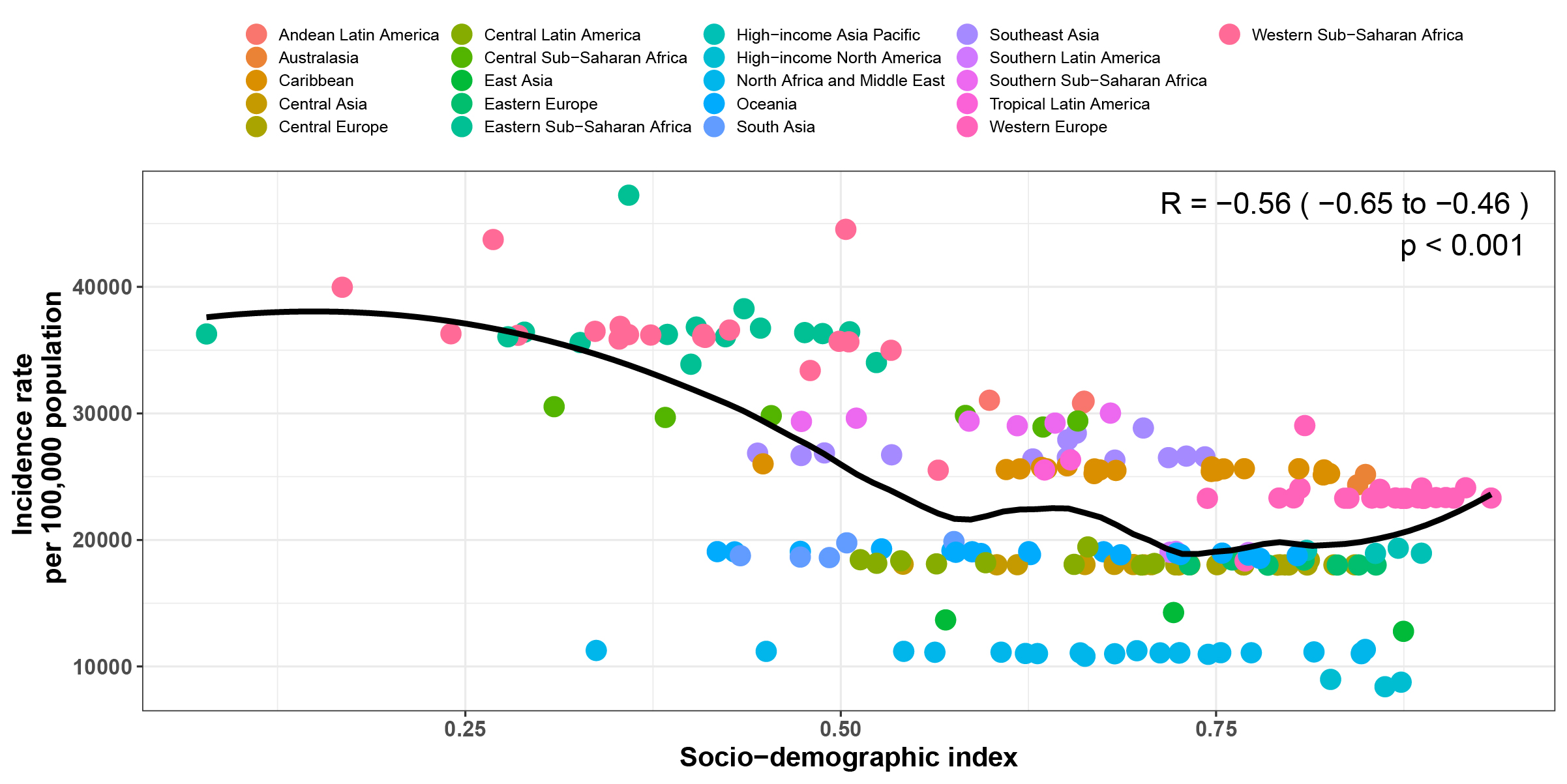

Supplement: Supplementary file 1 [file Datasheet1.zip › FSD supplementary material-20241205-v2/Figure S1-S33/Figure S32.jpg]

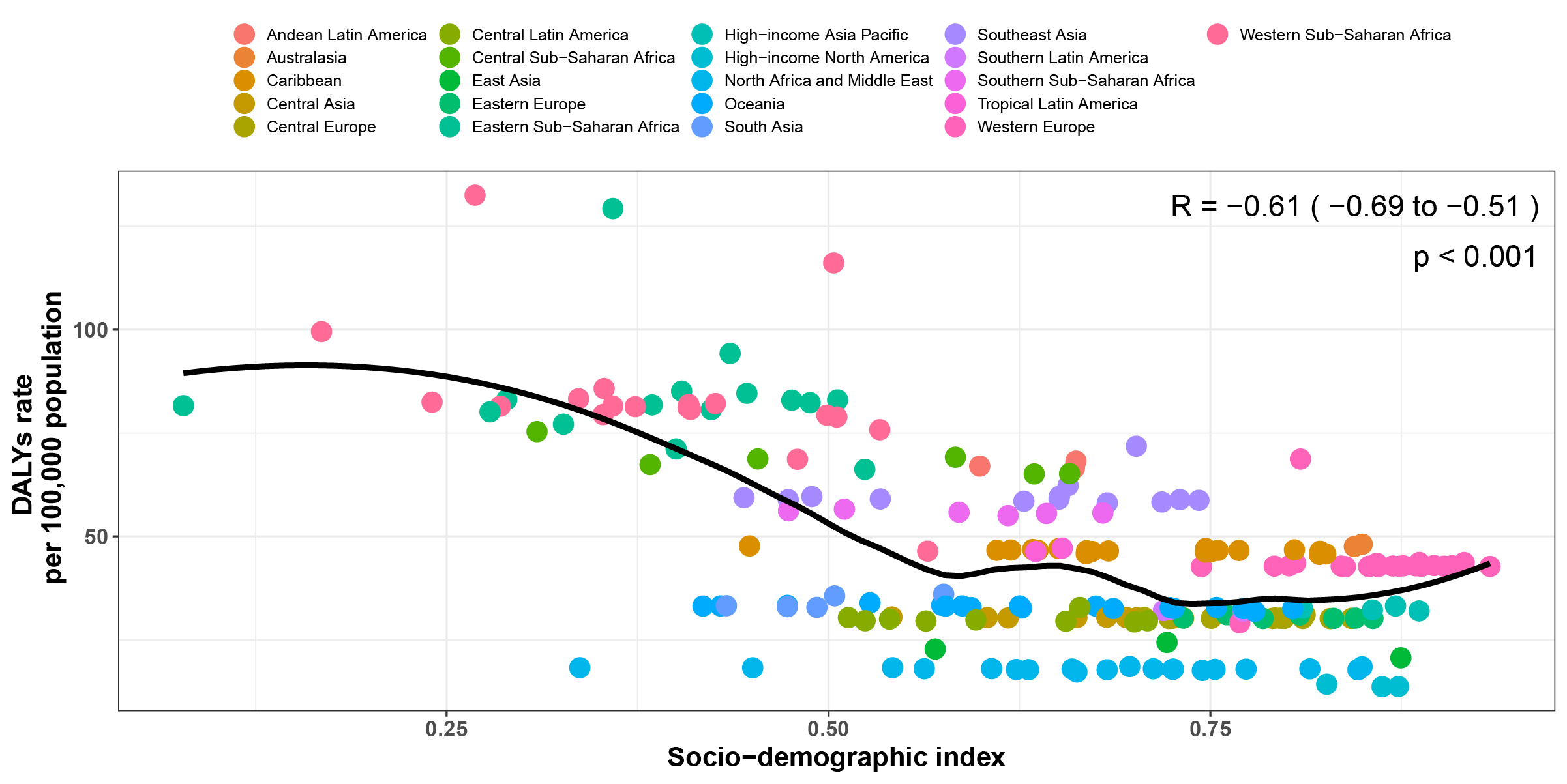

Supplement: Supplementary file 1 [file Datasheet1.zip › FSD supplementary material-20241205-v2/Figure S1-S33/Figure S33.jpg]

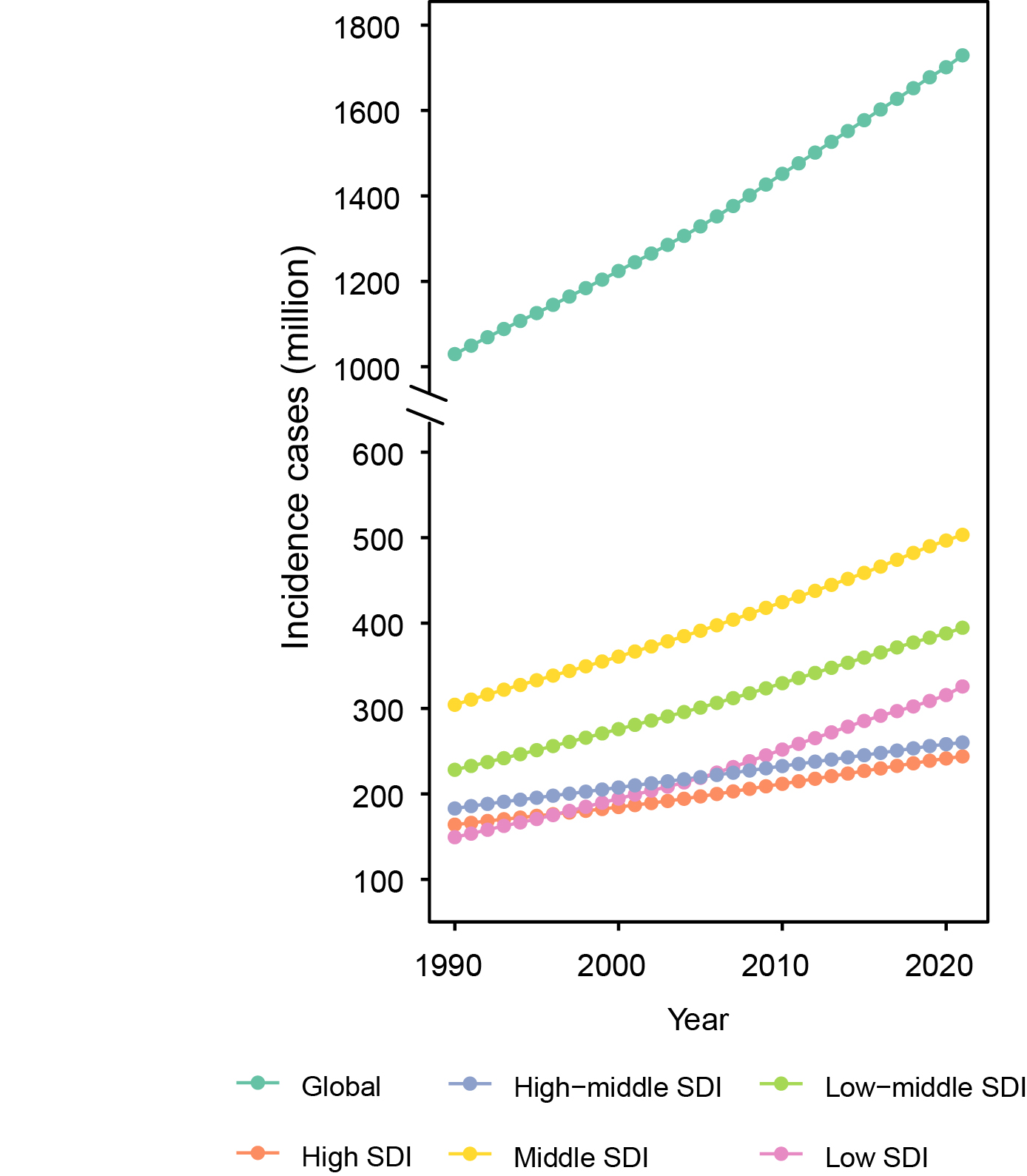

Supplement: Supplementary file 1 [file Datasheet1.zip › FSD supplementary material-20241205-v2/Figure S1-S33/Figure S4.jpg]

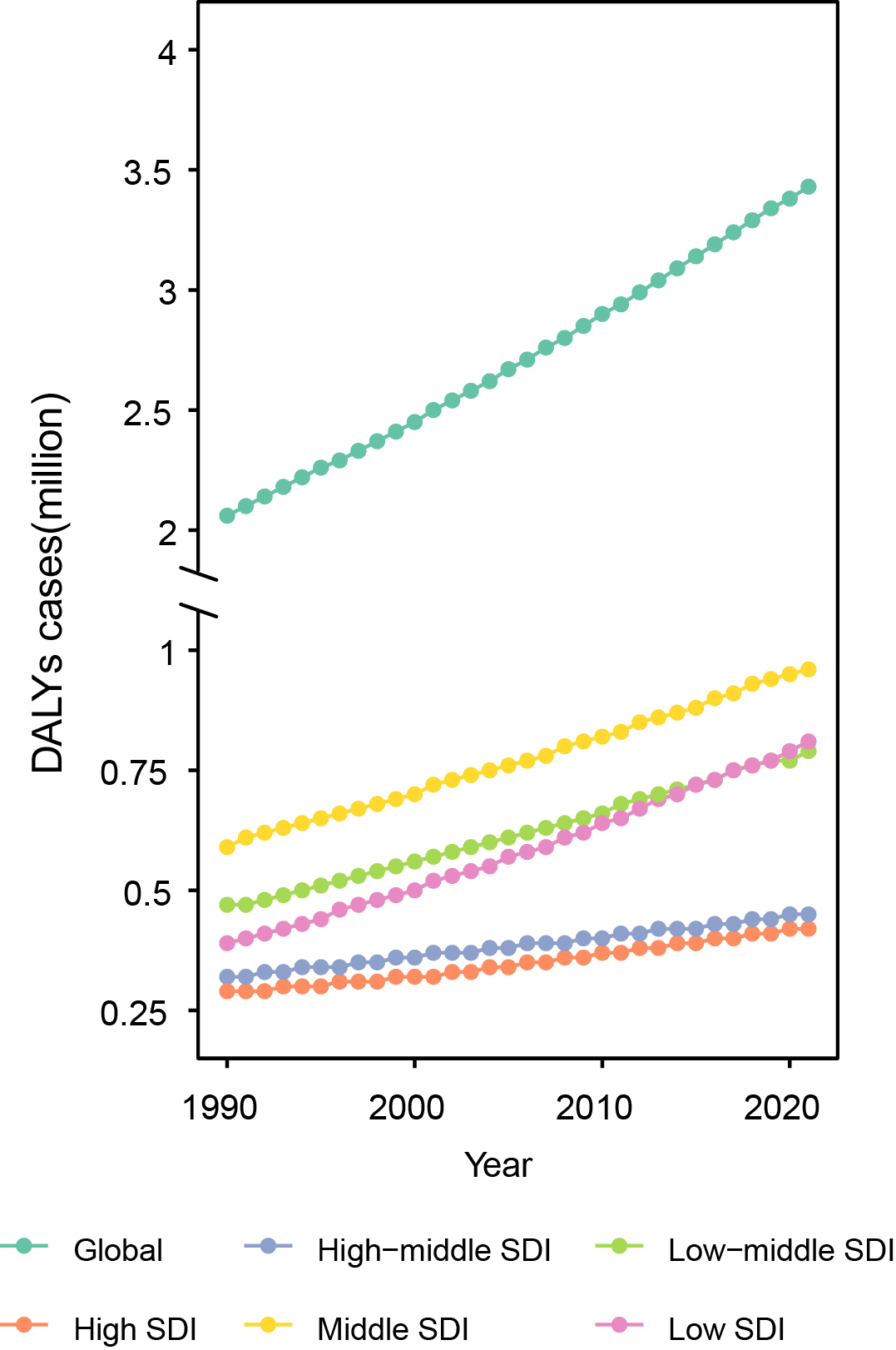

Supplement: Supplementary file 1 [file Datasheet1.zip › FSD supplementary material-20241205-v2/Figure S1-S33/Figure S5.jpg]

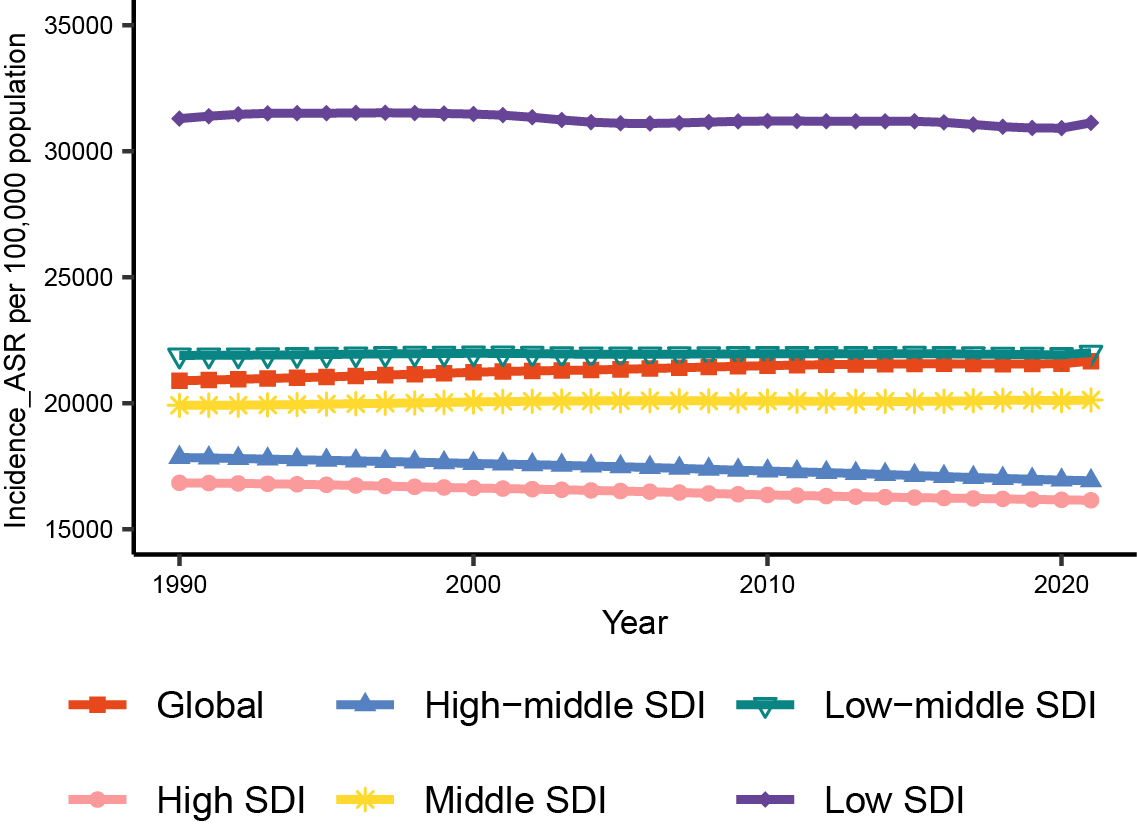

Supplement: Supplementary file 1 [file Datasheet1.zip › FSD supplementary material-20241205-v2/Figure S1-S33/Figure S6.jpg]

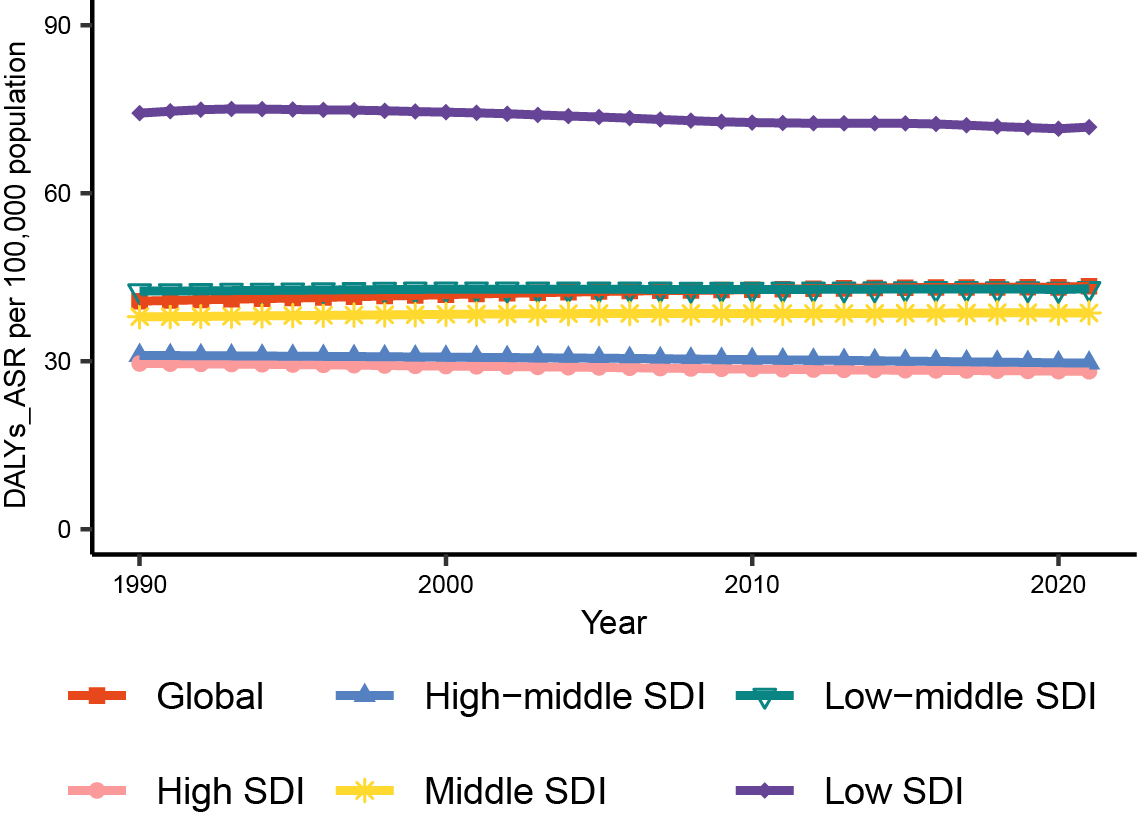

Supplement: Supplementary file 1 [file Datasheet1.zip › FSD supplementary material-20241205-v2/Figure S1-S33/Figure S7.jpg]

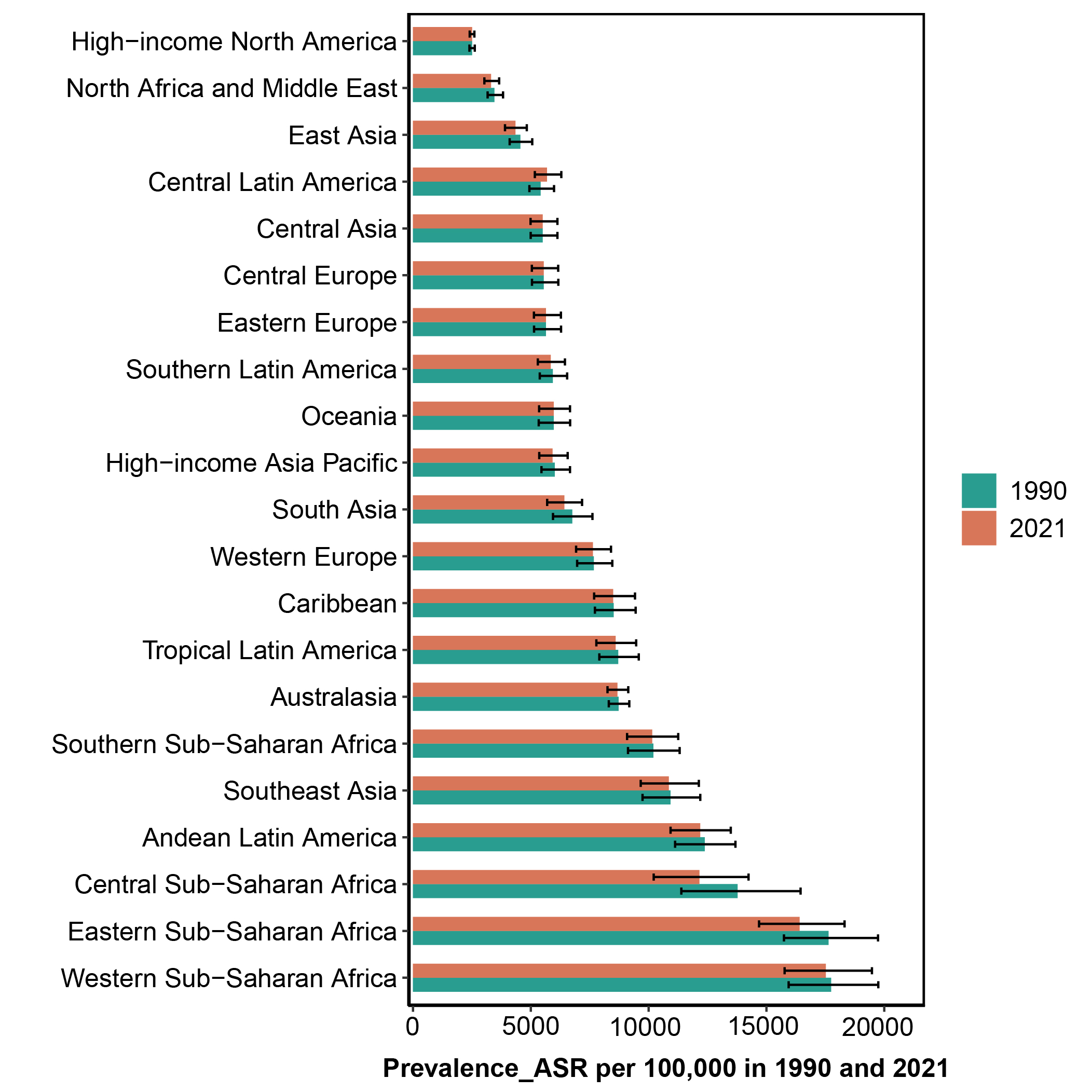

Supplement: Supplementary file 1 [file Datasheet1.zip › FSD supplementary material-20241205-v2/Figure S1-S33/Figure S8.jpg]

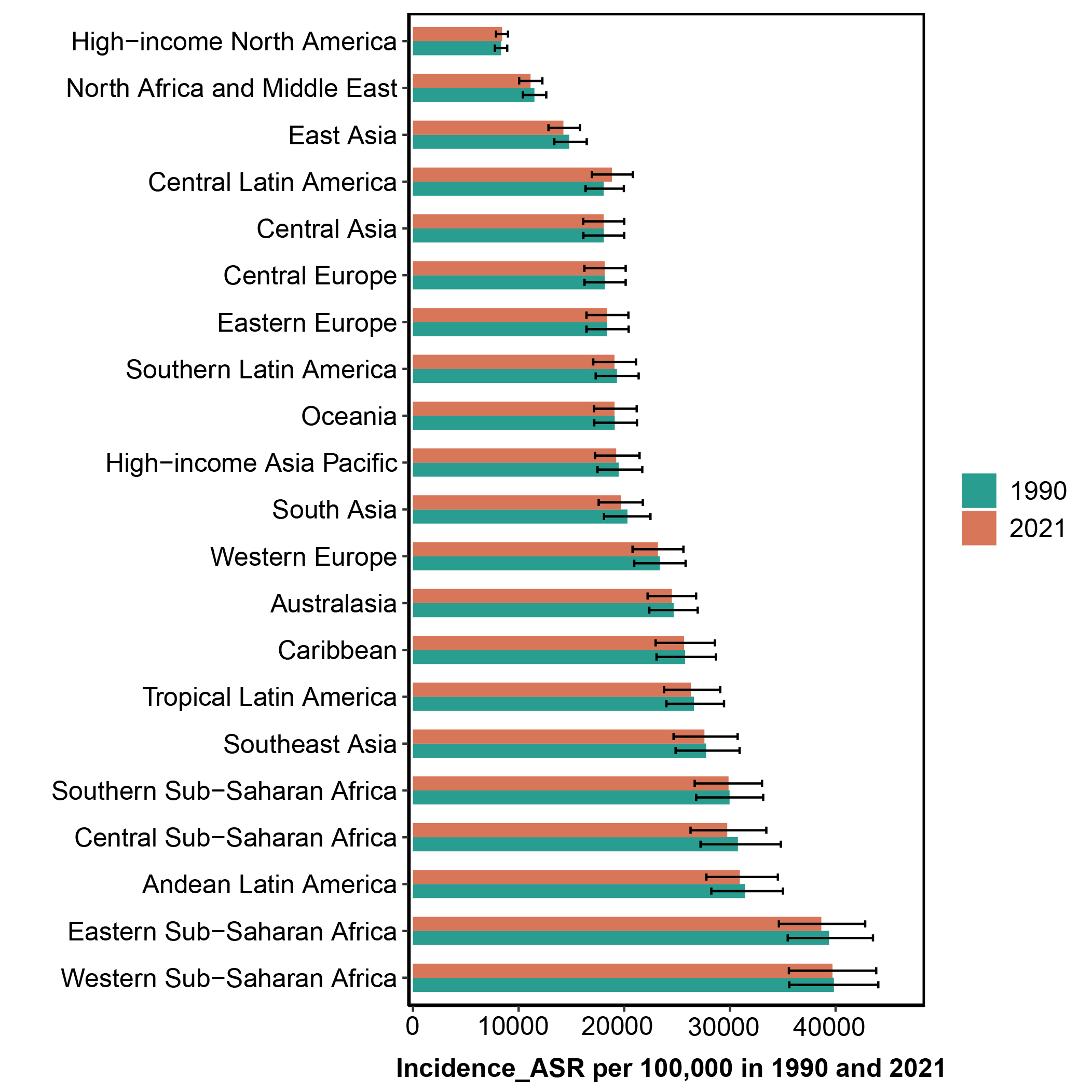

Supplement: Supplementary file 1 [file Datasheet1.zip › FSD supplementary material-20241205-v2/Figure S1-S33/Figure S9.jpg]
